# Supplementary figures and images for: Mitochondrial ETF insufficiency drives neoplastic growth by selectively optimizing cancer bioenergetics (part 1 of 2)
Source: eLife. 2026 May 5;14:RP106587. doi: 10.7554/eLife.106587 (PMC13143275; doi:10.7554/eLife.106587)

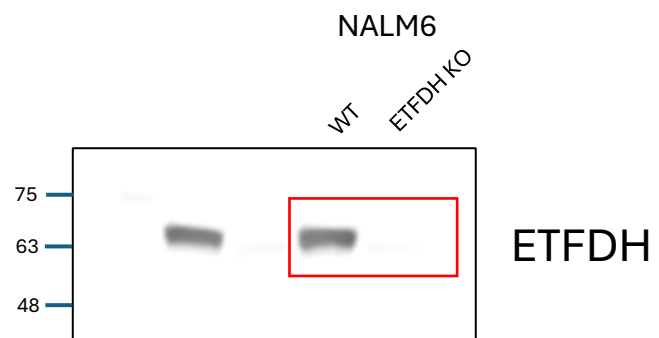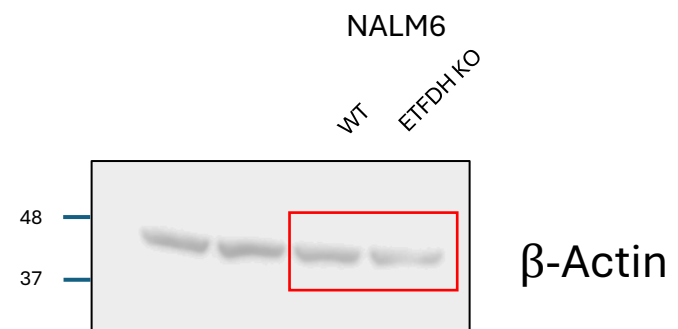

Supplement: Figure 1—figure supplement 1—source data 1. [file elife-106587-fig1-figsupp1-data1.zip › Figure 1-figure supplement 1 - source data 1/Figure 1-figure supplement 1H - source data 1/Figure 1-figure supplement 1H - source data 1.pdf]

## Slide 1
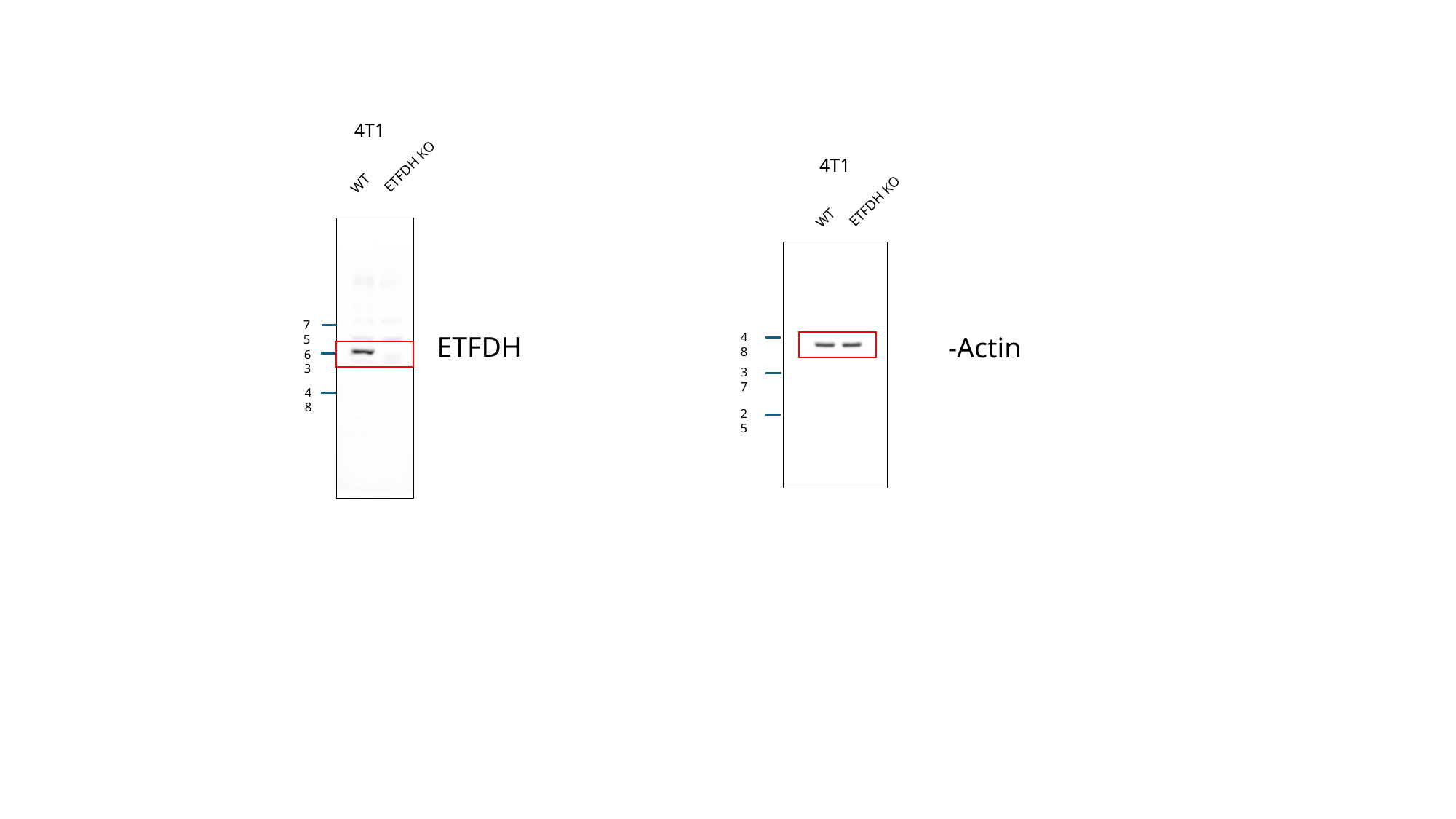

4T1
4T1
ETFDH KO
WT
ETFDH KO
WT
75
48
ETFDH
63
37
48
25

Supplement: Figure 1—figure supplement 1—source data 1. [file elife-106587-fig1-figsupp1-data1.zip › Figure 1-figure supplement 1 - source data 1/Figure 1-figure supplement 1G - source data 1/Figure 1-figure supplement 1G - source data 1.pptx]

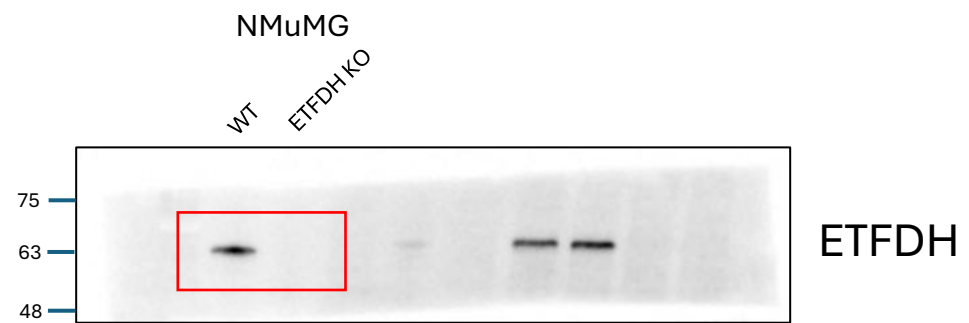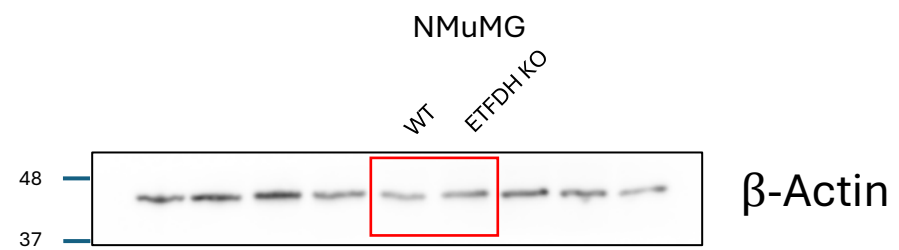

Supplement: Figure 1—figure supplement 1—source data 1. [file elife-106587-fig1-figsupp1-data1.zip › Figure 1-figure supplement 1 - source data 1/Figure 1-figure supplement 1I - source data 1/Figure 1-figure supplement 1I - source data 1.pdf]

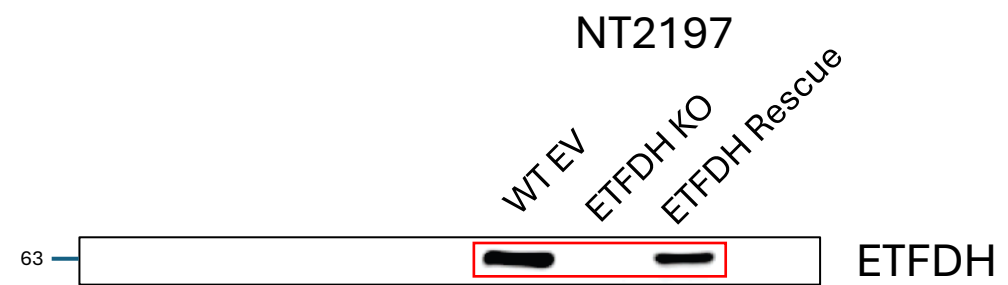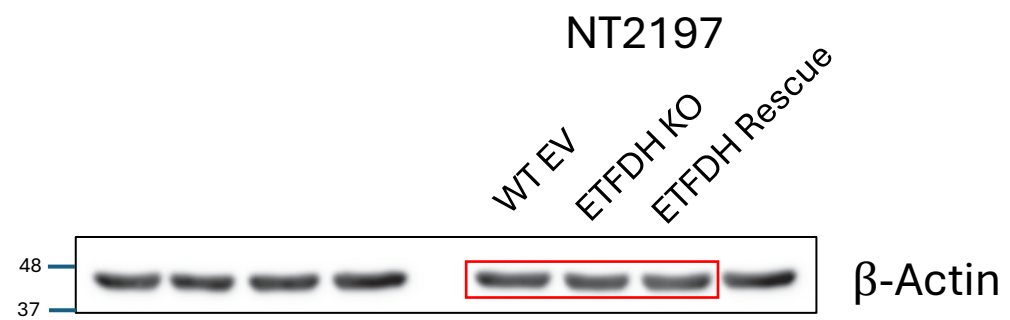

Supplement: Figure 1—figure supplement 1—source data 1. [file elife-106587-fig1-figsupp1-data1.zip › Figure 1-figure supplement 1 - source data 1/Figure 1-figure supplement 1F - source data 1/Figure 1-figure supplement 1F - source data 1.pdf]

HCT-116

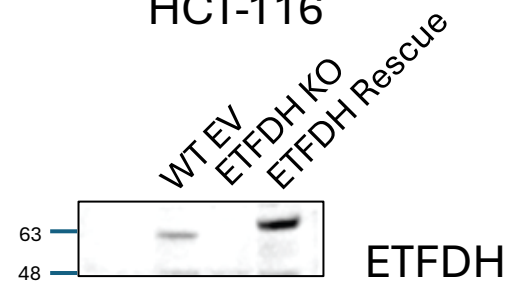

HCT-116

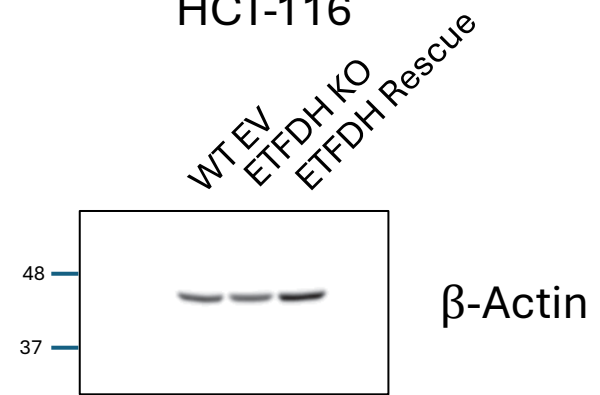

Supplement: Figure 1—figure supplement 1—source data 1. [file elife-106587-fig1-figsupp1-data1.zip › Figure 1-figure supplement 1 - source data 1/Figure 1-figure supplement 1E - source data 1/Figure 1-figure supplement 1E - source data 1.pdf]

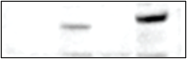

Supplement: Figure 1—figure supplement 1—source data 2. [file elife-106587-fig1-figsupp1-data2.zip › Figure 1-figure supplement 1 - source data 2/Figure 1-figure supplement 1E - source data 2/Figure 1-figure supplement 1E - ETFDH.tif]

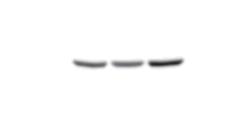

Supplement: Figure 1—figure supplement 1—source data 2. [file elife-106587-fig1-figsupp1-data2.zip › Figure 1-figure supplement 1 - source data 2/Figure 1-figure supplement 1E - source data 2/Figure 1-figure supplement 1E - B_Actin.tif]

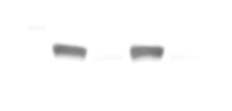

Supplement: Figure 1—figure supplement 1—source data 2. [file elife-106587-fig1-figsupp1-data2.zip › Figure 1-figure supplement 1 - source data 2/Figure 1-figure supplement 1H - source data 2/Figure 1-figure supplement 1H - ETFDH.tif]

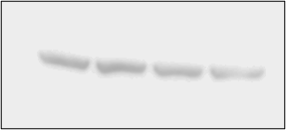

Supplement: Figure 1—figure supplement 1—source data 2. [file elife-106587-fig1-figsupp1-data2.zip › Figure 1-figure supplement 1 - source data 2/Figure 1-figure supplement 1H - source data 2/Figure 1-figure supplement 1H - B_Actin.tif]

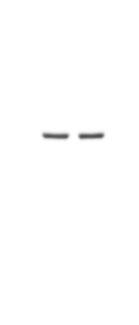

Supplement: Figure 1—figure supplement 1—source data 2. [file elife-106587-fig1-figsupp1-data2.zip › Figure 1-figure supplement 1 - source data 2/Figure 1-figure supplement 1G - source data 2/Figure 1-figure supplement 1G - B_Actin.tif]

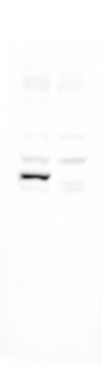

Supplement: Figure 1—figure supplement 1—source data 2. [file elife-106587-fig1-figsupp1-data2.zip › Figure 1-figure supplement 1 - source data 2/Figure 1-figure supplement 1G - source data 2/Figure 1-figure supplement 1G - ETFDH.tif]

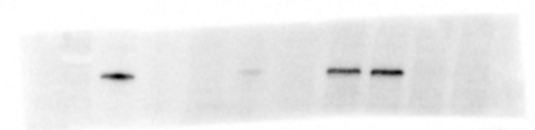

Supplement: Figure 1—figure supplement 1—source data 2. [file elife-106587-fig1-figsupp1-data2.zip › Figure 1-figure supplement 1 - source data 2/Figure 1-figure supplement 1I - source data 2/Figure 1-figure supplement 1I - ETFDH.tif]

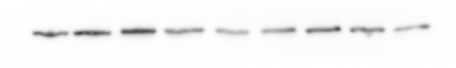

Supplement: Figure 1—figure supplement 1—source data 2. [file elife-106587-fig1-figsupp1-data2.zip › Figure 1-figure supplement 1 - source data 2/Figure 1-figure supplement 1I - source data 2/Figure 1-figure supplement 1I - B_Actin.tif]

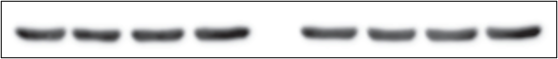

Supplement: Figure 1—figure supplement 1—source data 2. [file elife-106587-fig1-figsupp1-data2.zip › Figure 1-figure supplement 1 - source data 2/Figure 1-figure supplement 1F - source data 2/Figure 1-figure supplement1F - B_Actin.tif]

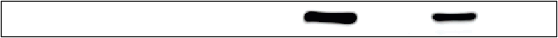

Supplement: Figure 1—figure supplement 1—source data 2. [file elife-106587-fig1-figsupp1-data2.zip › Figure 1-figure supplement 1 - source data 2/Figure 1-figure supplement 1F - source data 2/Figure 1-figure supplement 1F - ETFDH.tif]

HCT-116

WT ETFDH KO

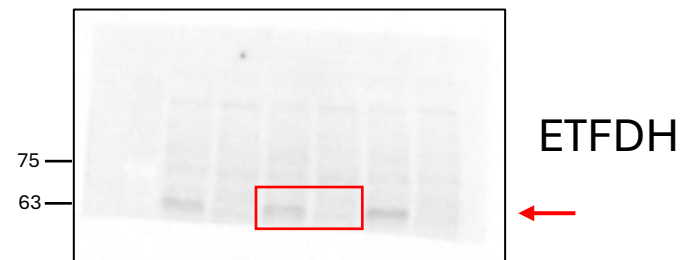

HCT-116

WT ETFDH KO

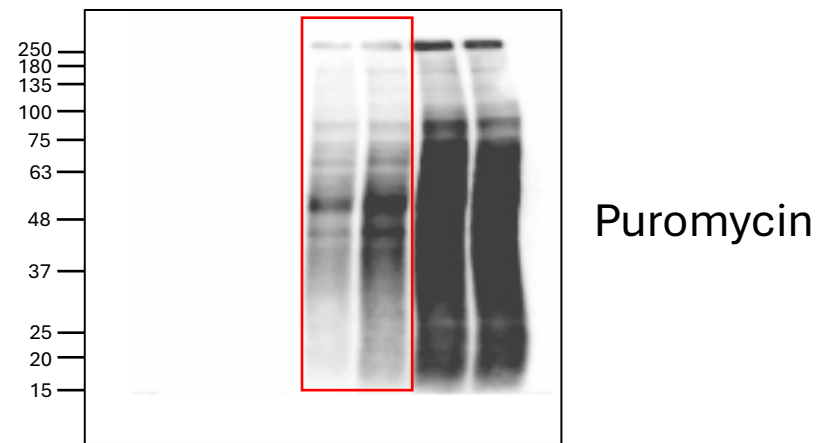

HCT-116

WT ETFDH KO

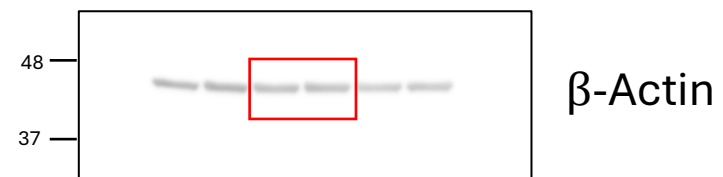

Supplement: Figure 3—source data 1. [file elife-106587-fig3-data1.zip › Figure 3 - source data 1/Figure 3B - source data 1/Figure 3B - source data 1.pdf]

## ETFDH

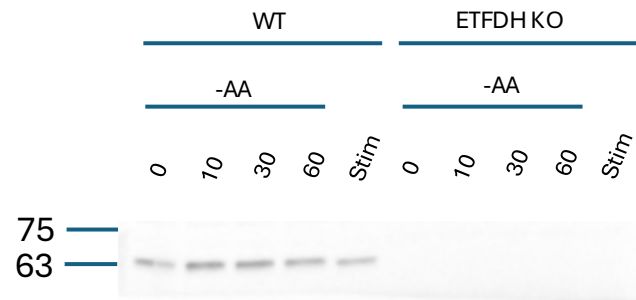

## pS6(S240/244)

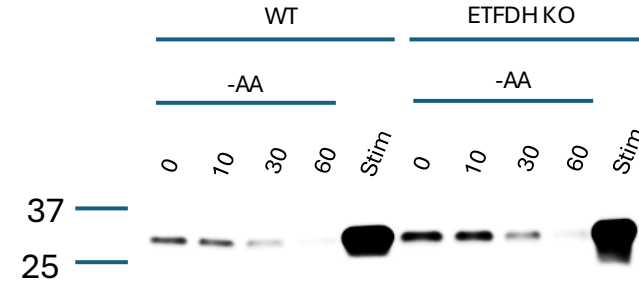

## S6

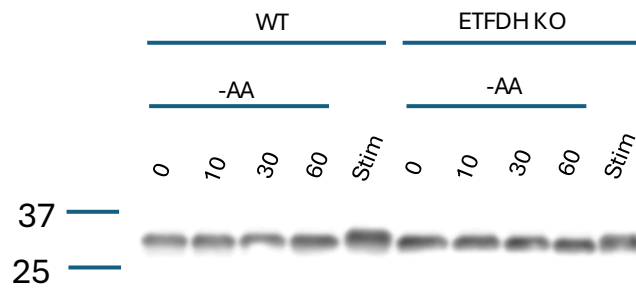

## p-4E-BP1 (S65)

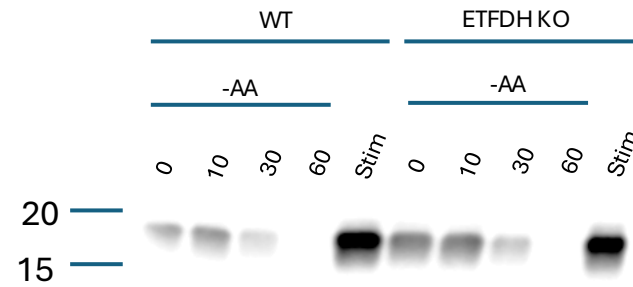

## 4E-BP1

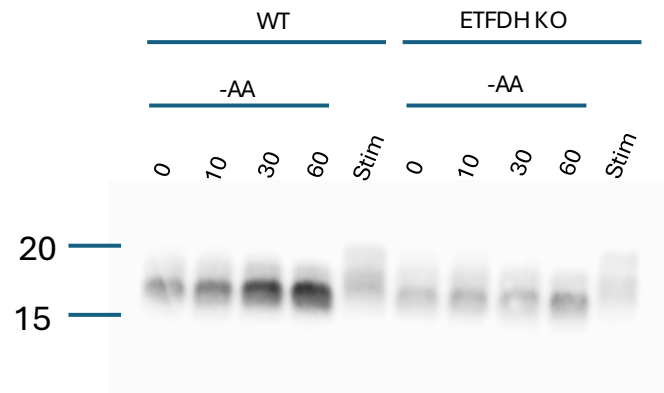

## 4E-BP2

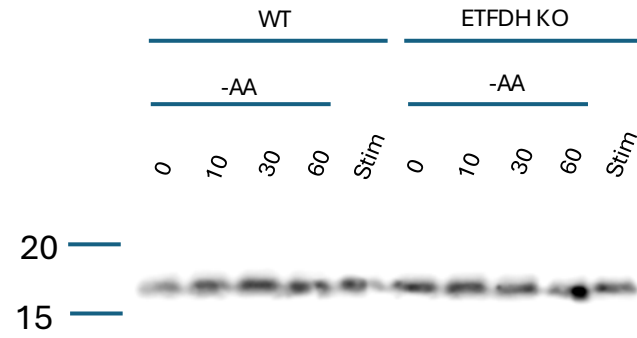

## $\beta$ -Actin

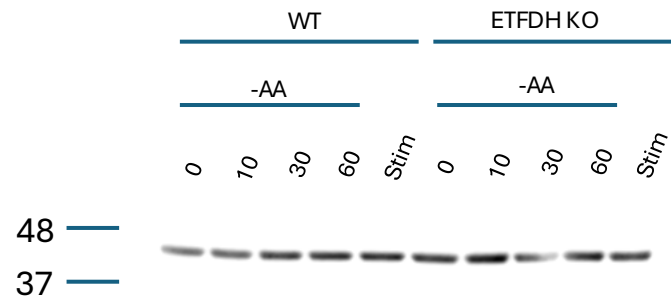

Supplement: Figure 3—source data 1. [file elife-106587-fig3-data1.zip › Figure 3 - source data 1/Figure 3E - source data 1/Figure 3E - source data 1.pdf]

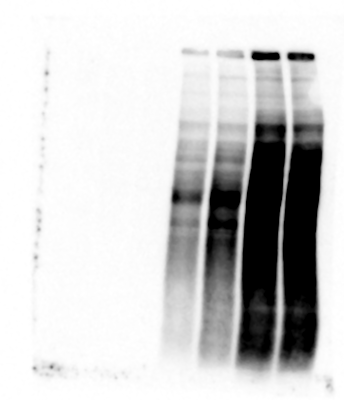

Supplement: Figure 3—source data 2. [file elife-106587-fig3-data2.zip › Figure 3 - source data 2/Figure 3B - source data 2/Figure 3B - Puromycin.tif]

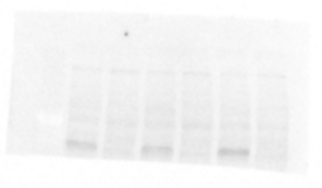

Supplement: Figure 3—source data 2. [file elife-106587-fig3-data2.zip › Figure 3 - source data 2/Figure 3B - source data 2/Figure 3B - ETFDH.tif]

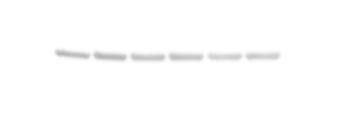

Supplement: Figure 3—source data 2. [file elife-106587-fig3-data2.zip › Figure 3 - source data 2/Figure 3B - source data 2/Figure 3B - B_Actin.tif]

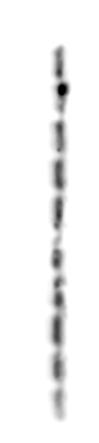

Supplement: Figure 3—source data 2. [file elife-106587-fig3-data2.zip › Figure 3 - source data 2/Figure 3E - source data 2/Figure 3E - 4E-BP2.tif]

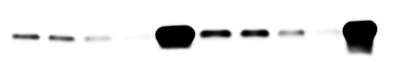

Supplement: Figure 3—source data 2. [file elife-106587-fig3-data2.zip › Figure 3 - source data 2/Figure 3E - source data 2/Figure 3E - pS6.tif]

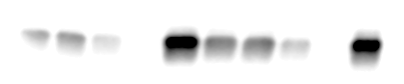

Supplement: Figure 3—source data 2. [file elife-106587-fig3-data2.zip › Figure 3 - source data 2/Figure 3E - source data 2/Figure 3E - p4E-BP1.tif]

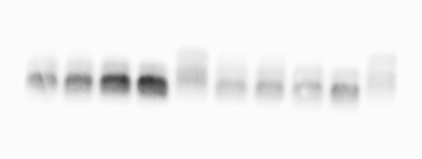

Supplement: Figure 3—source data 2. [file elife-106587-fig3-data2.zip › Figure 3 - source data 2/Figure 3E - source data 2/Figure 3E - 4E-BP1.tif]

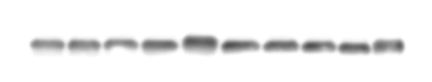

Supplement: Figure 3—source data 2. [file elife-106587-fig3-data2.zip › Figure 3 - source data 2/Figure 3E - source data 2/Figure 3E - S6.tif]

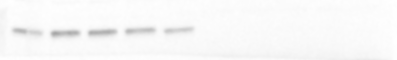

Supplement: Figure 3—source data 2. [file elife-106587-fig3-data2.zip › Figure 3 - source data 2/Figure 3E - source data 2/Figure 3E - ETFDH.tif]

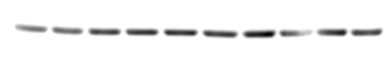

Supplement: Figure 3—source data 2. [file elife-106587-fig3-data2.zip › Figure 3 - source data 2/Figure 3E - source data 2/Figure 3E - B_Actin.tif]

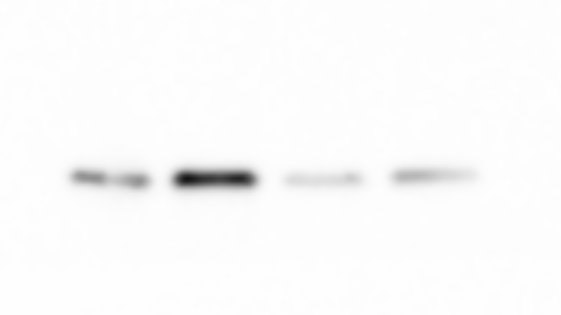

Supplement: Figure 3—source data 2. [file elife-106587-fig3-data2.zip › Figure 3 - source data 2/Figure 3D - source data 2/Figure 3D - NT2197 pS6.tif]

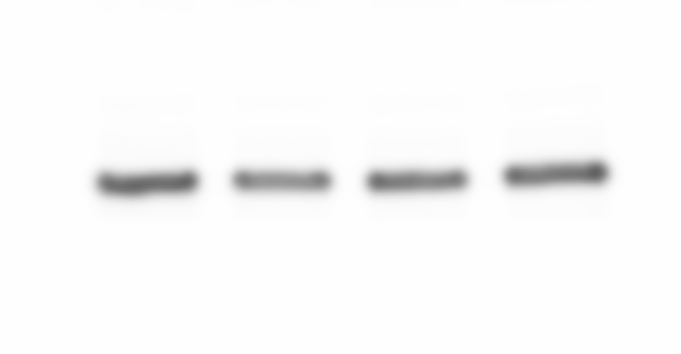

Supplement: Figure 3—source data 2. [file elife-106587-fig3-data2.zip › Figure 3 - source data 2/Figure 3D - source data 2/Figure 3D - NT2197 S6.tif]

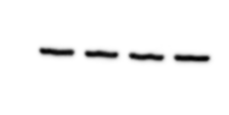

Supplement: Figure 3—source data 2. [file elife-106587-fig3-data2.zip › Figure 3 - source data 2/Figure 3D - source data 2/Figure 3D - NT2197 B_Actin.tif]

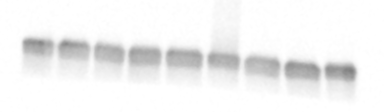

Supplement: Figure 3—source data 2. [file elife-106587-fig3-data2.zip › Figure 3 - source data 2/Figure 3D - source data 2/Figure 3D - HCT-116 S6.tif]

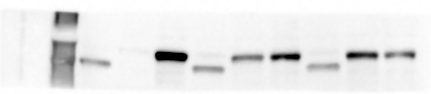

Supplement: Figure 3—source data 2. [file elife-106587-fig3-data2.zip › Figure 3 - source data 2/Figure 3D - source data 2/Figure 3D - HCT-116 ETFDH.tif]

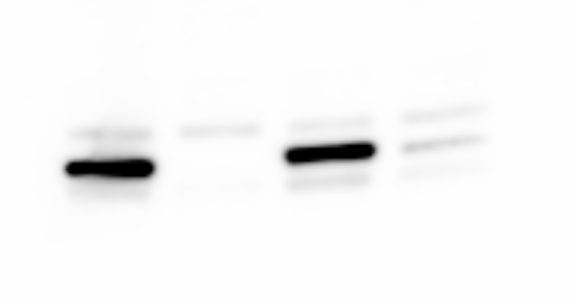

Supplement: Figure 3—source data 2. [file elife-106587-fig3-data2.zip › Figure 3 - source data 2/Figure 3D - source data 2/Figure 3D - NT2197 ETFDH.tif]

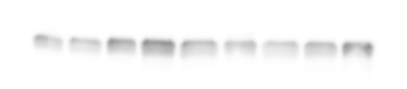

Supplement: Figure 3—source data 2. [file elife-106587-fig3-data2.zip › Figure 3 - source data 2/Figure 3D - source data 2/Figure 3D - HCT-116 pS6.tif]

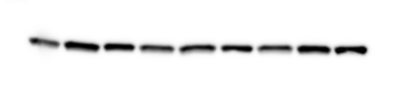

Supplement: Figure 3—source data 2. [file elife-106587-fig3-data2.zip › Figure 3 - source data 2/Figure 3D - source data 2/Figure 3D - HCT-116 B_Actin.tif]

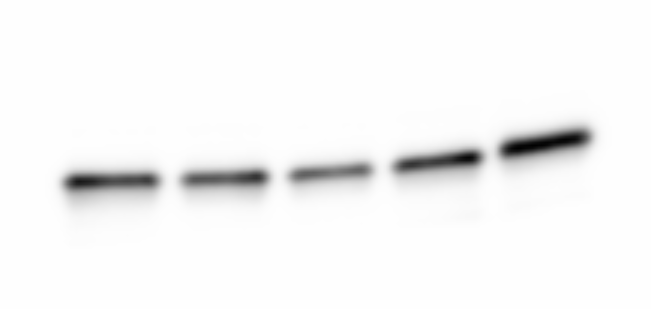

Supplement: Figure 3—source data 2. [file elife-106587-fig3-data2.zip › Figure 3 - source data 2/Figure 3C - source data 2/Figure 3C - NT2197 S6.tif]

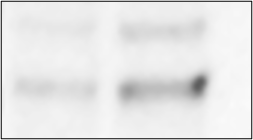

Supplement: Figure 3—source data 2. [file elife-106587-fig3-data2.zip › Figure 3 - source data 2/Figure 3C - source data 2/Figure 3C - NT2197 S6K.tif]

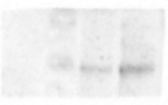

Supplement: Figure 3—source data 2. [file elife-106587-fig3-data2.zip › Figure 3 - source data 2/Figure 3C - source data 2/Figure 3C - HCT-116 pS6K.tif]

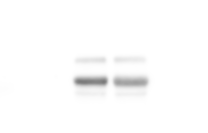

Supplement: Figure 3—source data 2. [file elife-106587-fig3-data2.zip › Figure 3 - source data 2/Figure 3C - source data 2/Figure 3C - HCT-116 S6K.tif]

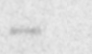

Supplement: Figure 3—source data 2. [file elife-106587-fig3-data2.zip › Figure 3 - source data 2/Figure 3C - source data 2/Figure 3C - HCT-116 ETFDH.tif]

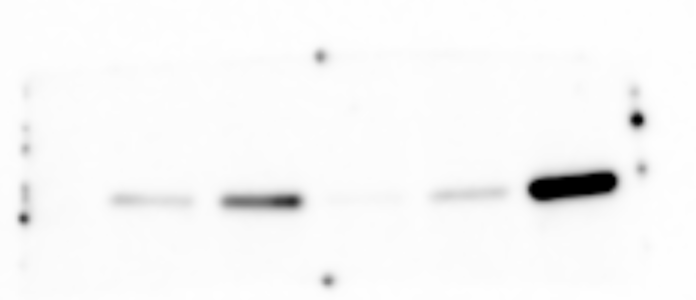

Supplement: Figure 3—source data 2. [file elife-106587-fig3-data2.zip › Figure 3 - source data 2/Figure 3C - source data 2/Figure 3C - NT2197 pS6K.tif]

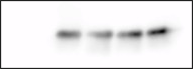

Supplement: Figure 3—source data 2. [file elife-106587-fig3-data2.zip › Figure 3 - source data 2/Figure 3C - source data 2/Figure 3C - HCT-116 S6.tif]

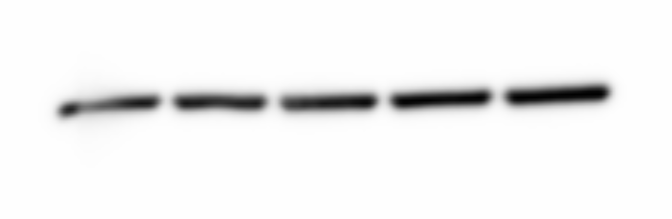

Supplement: Figure 3—source data 2. [file elife-106587-fig3-data2.zip › Figure 3 - source data 2/Figure 3C - source data 2/Figure 3C - NT2197 B_Actin.tif]

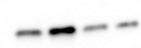

Supplement: Figure 3—source data 2. [file elife-106587-fig3-data2.zip › Figure 3 - source data 2/Figure 3C - source data 2/Figure 3C - HCT-116 pS6.tif]

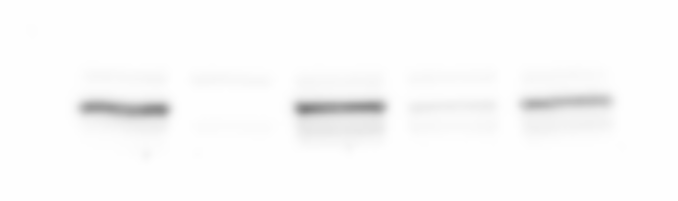

Supplement: Figure 3—source data 2. [file elife-106587-fig3-data2.zip › Figure 3 - source data 2/Figure 3C - source data 2/Figure 3C - NT2197 ETFDH.tif]

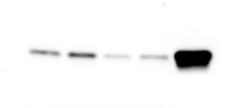

Supplement: Figure 3—source data 2. [file elife-106587-fig3-data2.zip › Figure 3 - source data 2/Figure 3C - source data 2/Figure 3C - NT2197 pS6.tif]

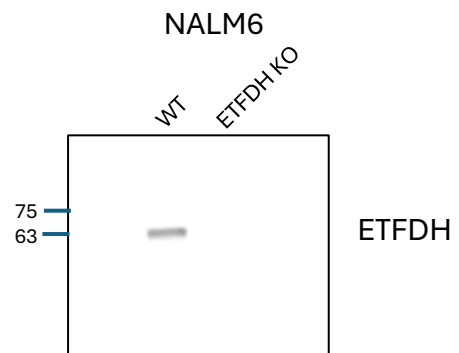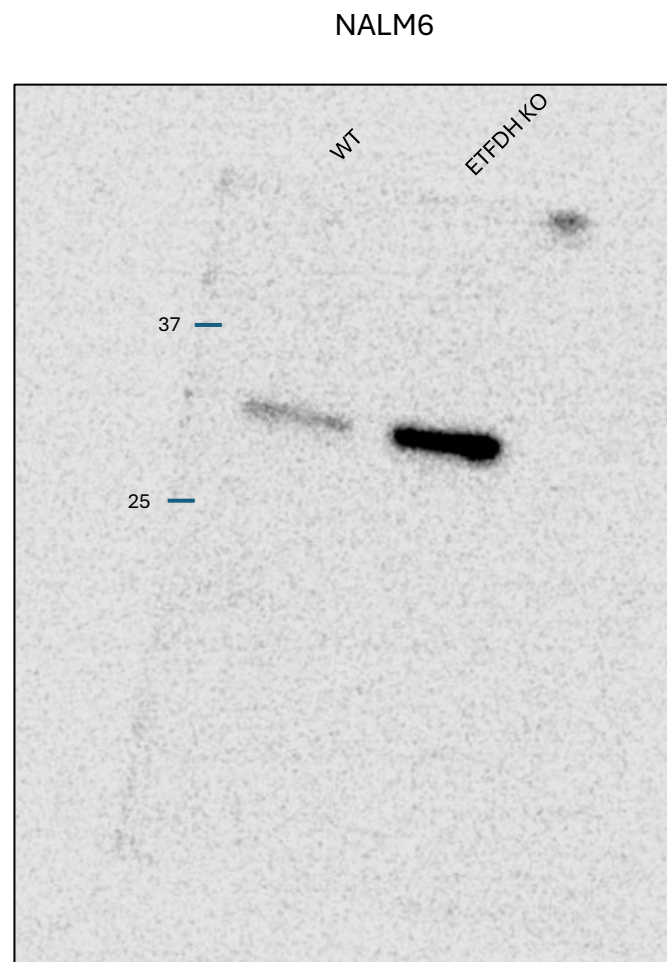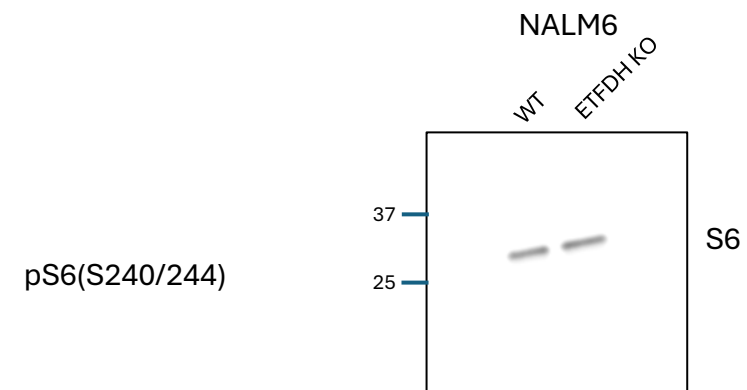

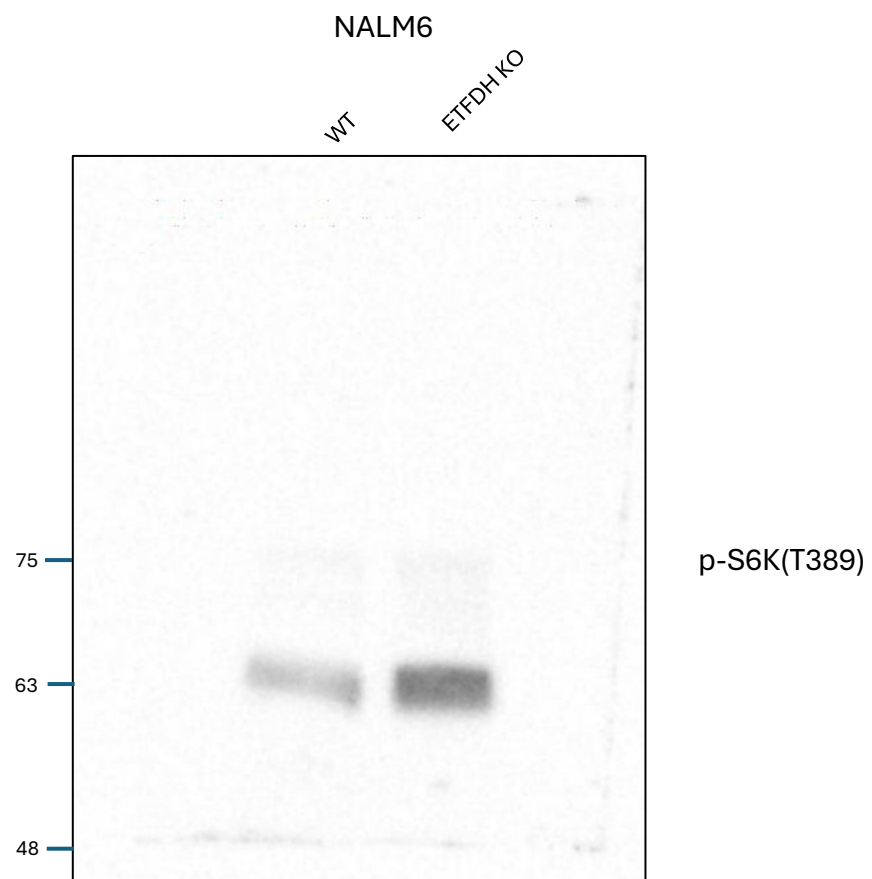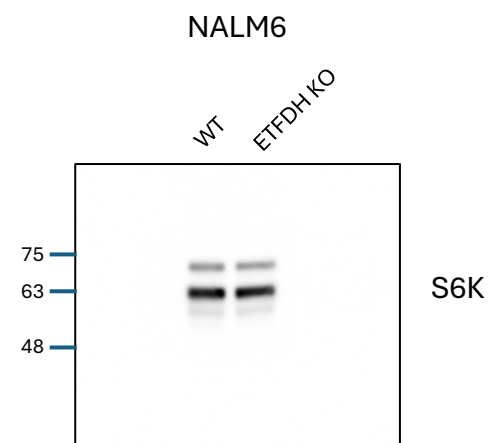

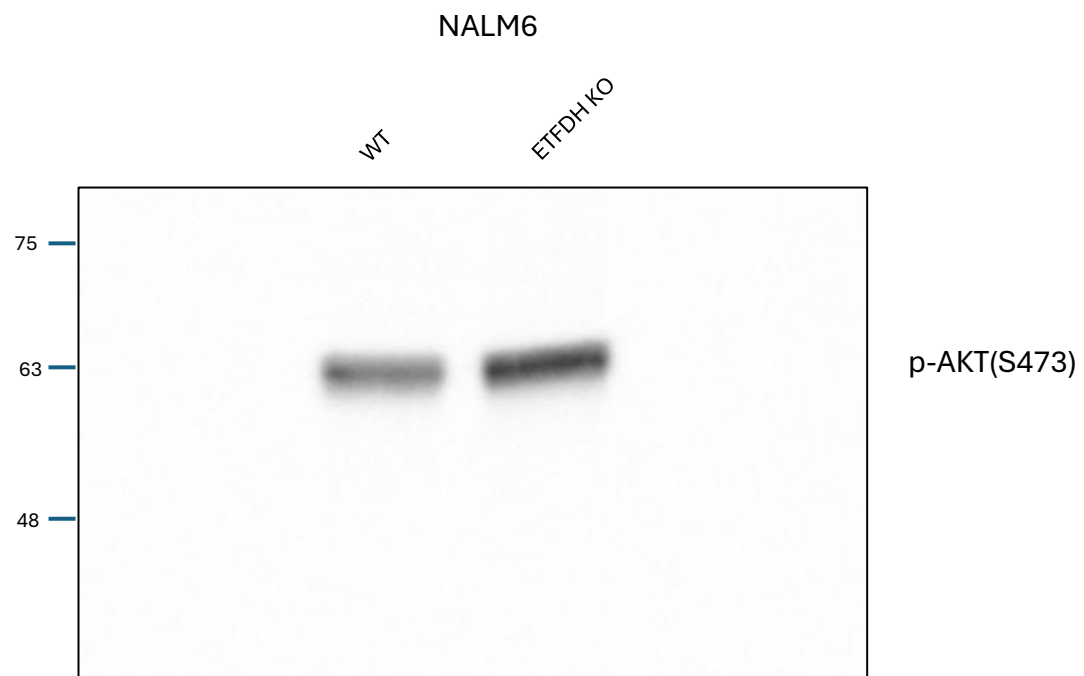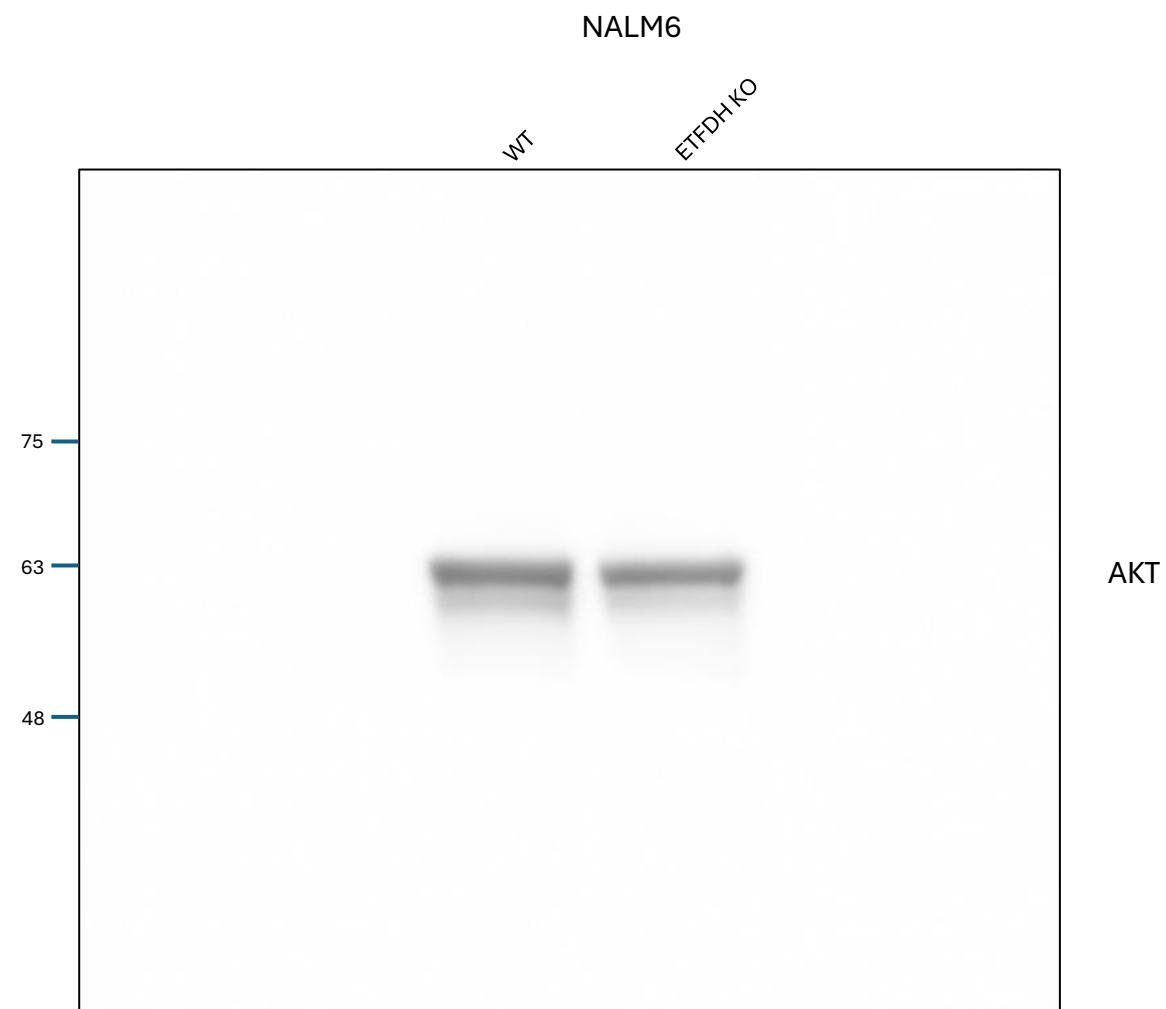

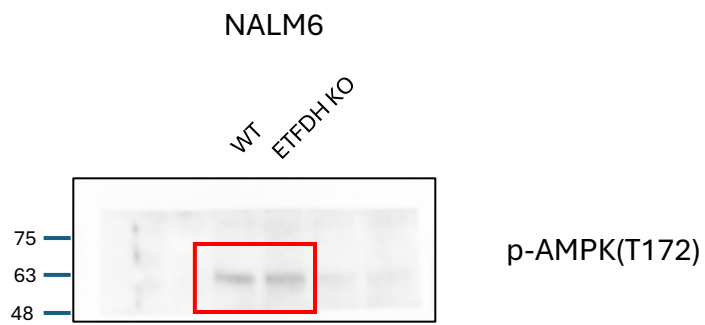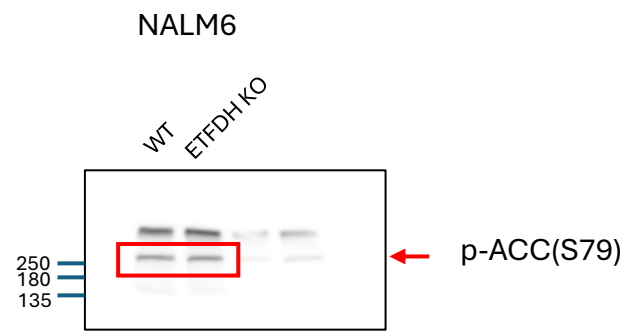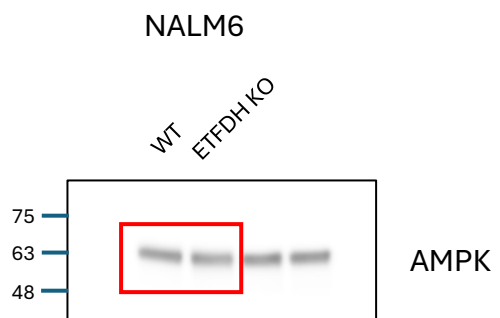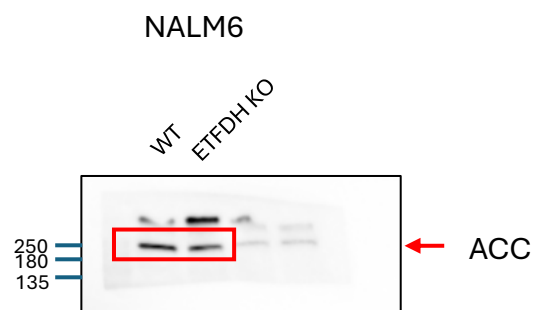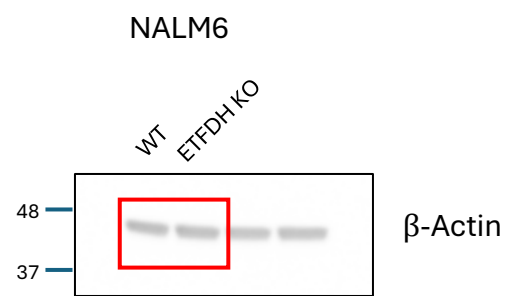

Supplement: Figure 3—figure supplement 1—source data 1. [file elife-106587-fig3-figsupp1-data1.zip › Figure 3-figure supplement 1 - source data 1/Figure 3-figure supplement 1B - source data 1/Figure 3-figure supplement 1B - source data 1.pdf]

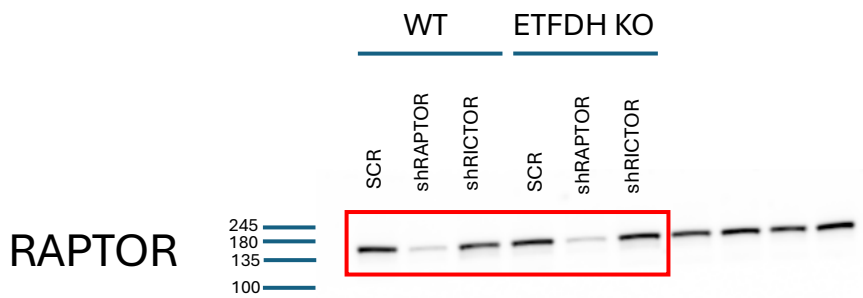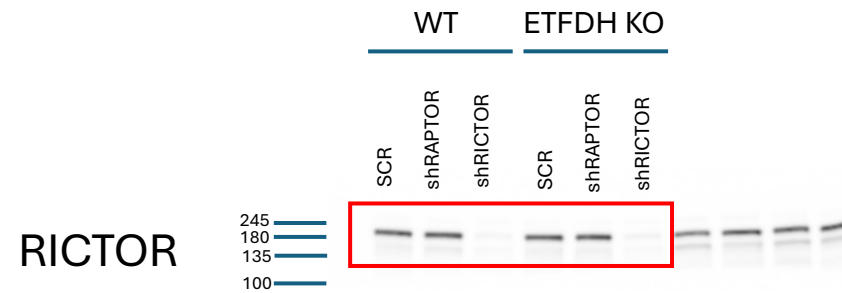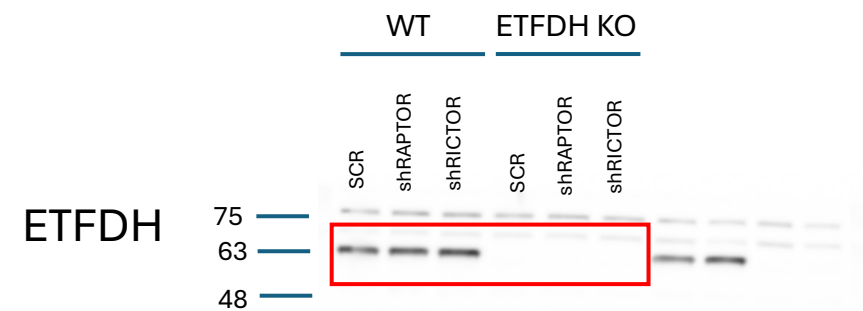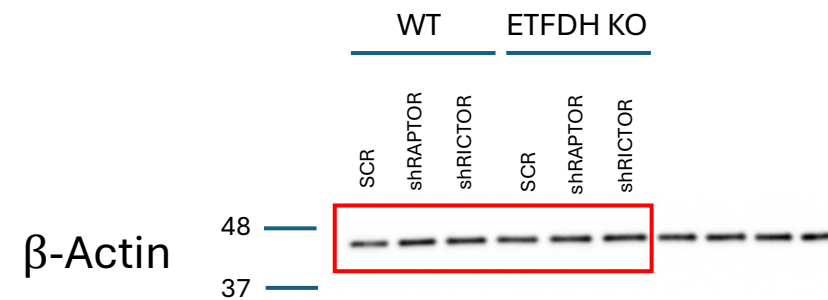

Supplement: Figure 3—figure supplement 1—source data 1. [file elife-106587-fig3-figsupp1-data1.zip › Figure 3-figure supplement 1 - source data 1/Figure 3-figure supplement 1D - source data 1/Figure 3-figure supplement 1D - source data.pdf]

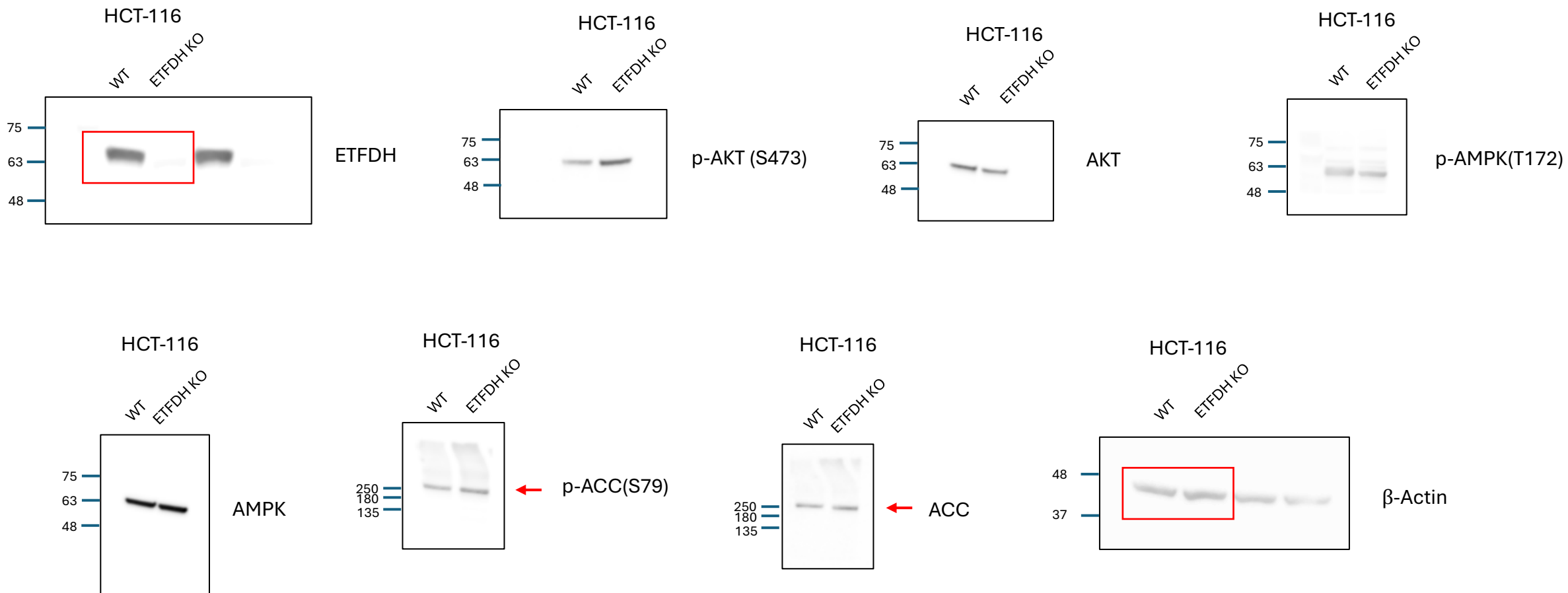

Supplement: Figure 3—figure supplement 1—source data 1. [file elife-106587-fig3-figsupp1-data1.zip › Figure 3-figure supplement 1 - source data 1/Figure 3-figure supplement 1A - source data 1/Figure 3-figure supplement 1A - source data 1.pdf]

## ETFDH

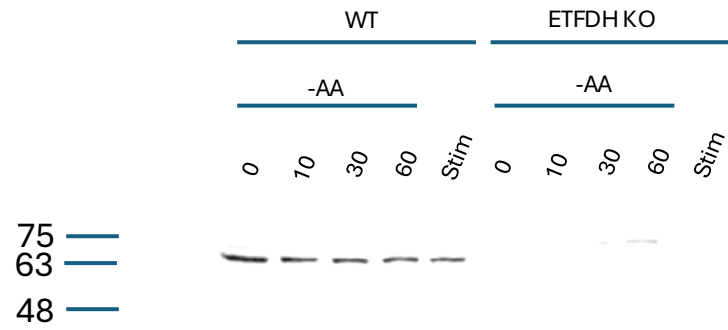

## p-eIF2 $\alpha$ (S51)

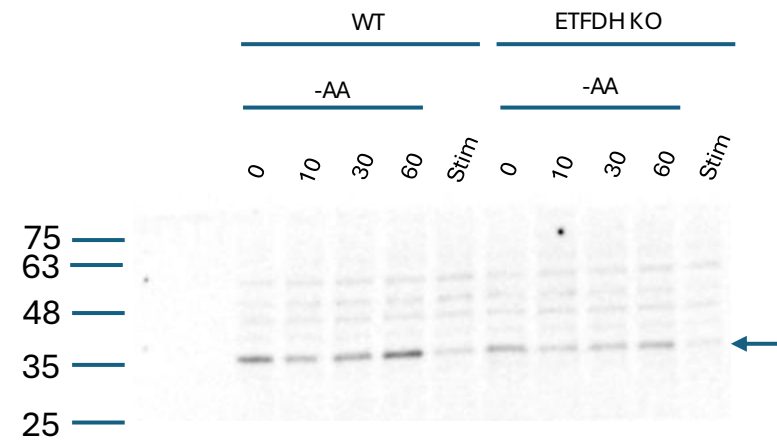

## eIF2 $\alpha$

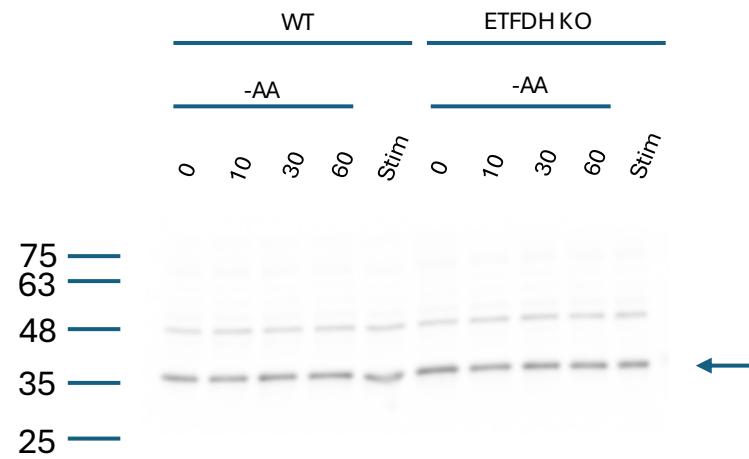

## ATF4

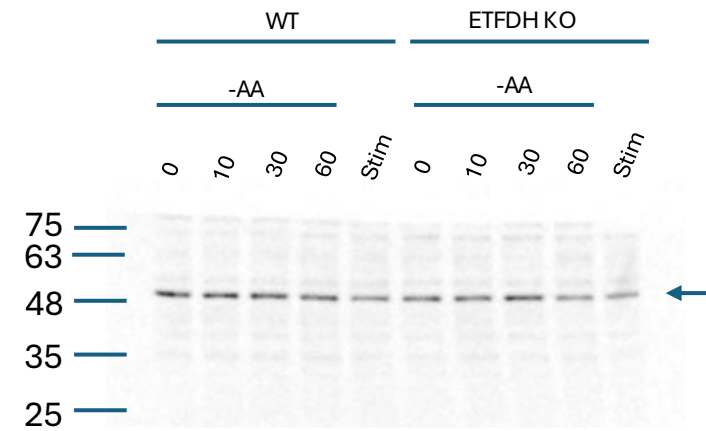

# $\beta$ -Actin

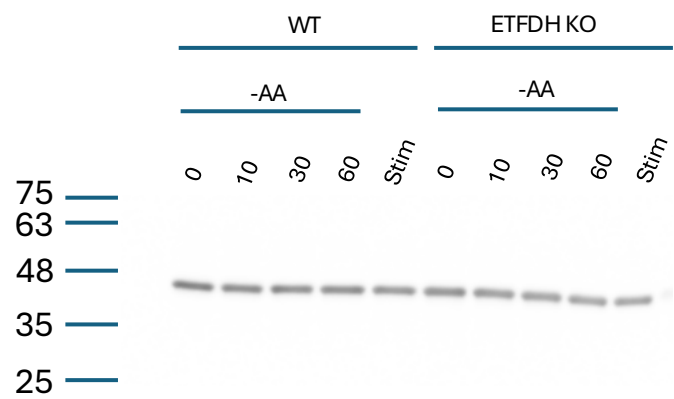

Supplement: Figure 3—figure supplement 1—source data 1. [file elife-106587-fig3-figsupp1-data1.zip › Figure 3-figure supplement 1 - source data 1/Figure 3-figure supplement 1H - source data 1/Figure 3-figure supplement 1H - source data 1.pdf]

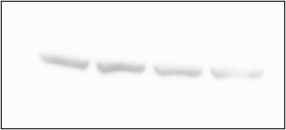

Supplement: Figure 3—figure supplement 1—source data 2. [file elife-106587-fig3-figsupp1-data2.zip › Figure 3-figure supplement 1 - source data 2/Figure 3-figure supplement 1A - source data 2/Figure 3-figure supplement 1A - B_Actin.tif]

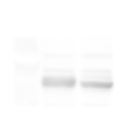

Supplement: Figure 3—figure supplement 1—source data 2. [file elife-106587-fig3-figsupp1-data2.zip › Figure 3-figure supplement 1 - source data 2/Figure 3-figure supplement 1A - source data 2/Figure 3-figure supplement 1A - pAMPK.tif]

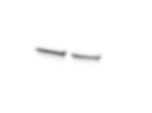

Supplement: Figure 3—figure supplement 1—source data 2. [file elife-106587-fig3-figsupp1-data2.zip › Figure 3-figure supplement 1 - source data 2/Figure 3-figure supplement 1A - source data 2/Figure 3-figure supplement 1A - AKT.tif]

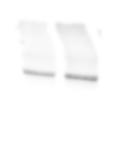

Supplement: Figure 3—figure supplement 1—source data 2. [file elife-106587-fig3-figsupp1-data2.zip › Figure 3-figure supplement 1 - source data 2/Figure 3-figure supplement 1A - source data 2/Figure 3-figure supplement 1A - pACC.tif]

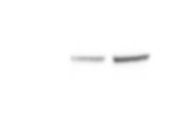

Supplement: Figure 3—figure supplement 1—source data 2. [file elife-106587-fig3-figsupp1-data2.zip › Figure 3-figure supplement 1 - source data 2/Figure 3-figure supplement 1A - source data 2/Figure 3-figure supplement 1A - pAKT.tif]

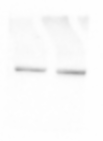

Supplement: Figure 3—figure supplement 1—source data 2. [file elife-106587-fig3-figsupp1-data2.zip › Figure 3-figure supplement 1 - source data 2/Figure 3-figure supplement 1A - source data 2/Figure 3-figure supplement 1A - ACC.tif]

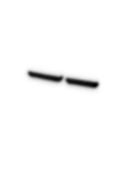

Supplement: Figure 3—figure supplement 1—source data 2. [file elife-106587-fig3-figsupp1-data2.zip › Figure 3-figure supplement 1 - source data 2/Figure 3-figure supplement 1A - source data 2/Figure 3-figure supplement 1A - AMPK.tif]

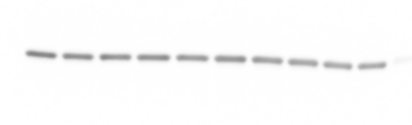

Supplement: Figure 3—figure supplement 1—source data 2. [file elife-106587-fig3-figsupp1-data2.zip › Figure 3-figure supplement 1 - source data 2/Figure 3-figure supplement 1H - source data 2/Figure 3-figure supplement 1H - B_Actin.tif]

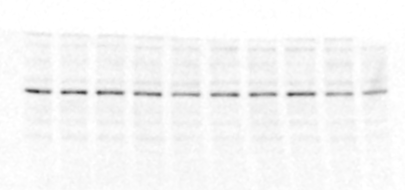

Supplement: Figure 3—figure supplement 1—source data 2. [file elife-106587-fig3-figsupp1-data2.zip › Figure 3-figure supplement 1 - source data 2/Figure 3-figure supplement 1H - source data 2/Figure 3-figure supplement 1H - ATF4.tif]

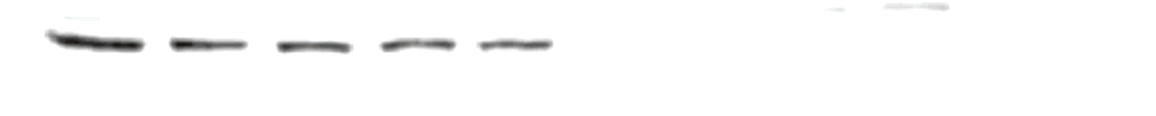

Supplement: Figure 3—figure supplement 1—source data 2. [file elife-106587-fig3-figsupp1-data2.zip › Figure 3-figure supplement 1 - source data 2/Figure 3-figure supplement 1H - source data 2/Figure 3-figure supplement 1H - ETFDH.tif]

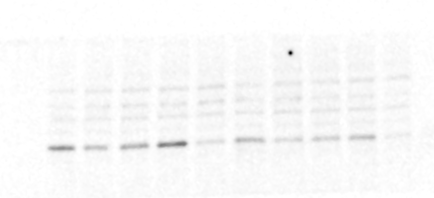

Supplement: Figure 3—figure supplement 1—source data 2. [file elife-106587-fig3-figsupp1-data2.zip › Figure 3-figure supplement 1 - source data 2/Figure 3-figure supplement 1H - source data 2/Figure 3-figure supplement 1H - peIF2alpha.tif]

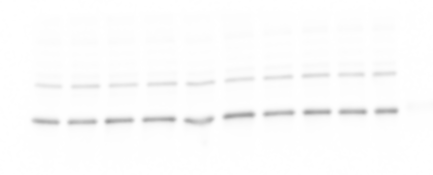

Supplement: Figure 3—figure supplement 1—source data 2. [file elife-106587-fig3-figsupp1-data2.zip › Figure 3-figure supplement 1 - source data 2/Figure 3-figure supplement 1H - source data 2/Figure 3-figure supplement 1H - eIF2alpha.tif]

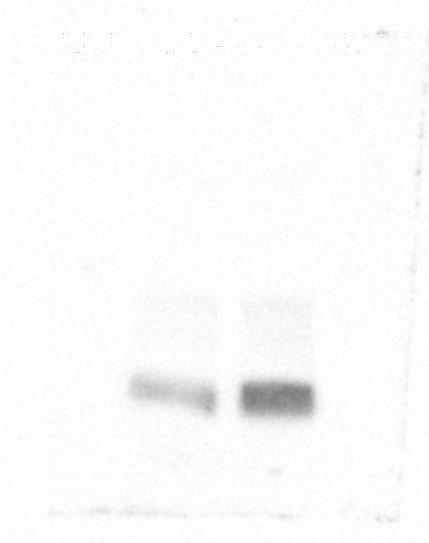

Supplement: Figure 3—figure supplement 1—source data 2. [file elife-106587-fig3-figsupp1-data2.zip › Figure 3-figure supplement 1 - source data 2/Figure 3-figure supplement 1B - source data 2/Figure 3-figure supplement 1B - pS6K.tif]

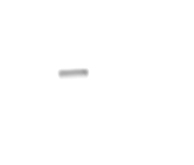

Supplement: Figure 3—figure supplement 1—source data 2. [file elife-106587-fig3-figsupp1-data2.zip › Figure 3-figure supplement 1 - source data 2/Figure 3-figure supplement 1B - source data 2/Figure 3-figure supplement 1B - ETFDH.tif]

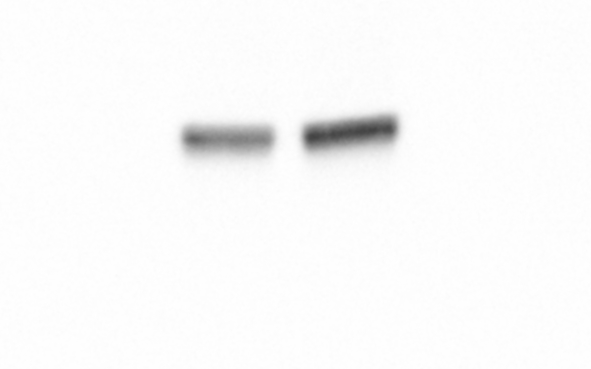

Supplement: Figure 3—figure supplement 1—source data 2. [file elife-106587-fig3-figsupp1-data2.zip › Figure 3-figure supplement 1 - source data 2/Figure 3-figure supplement 1B - source data 2/Figure 3-figure supplement 1B - pAKT.tif]

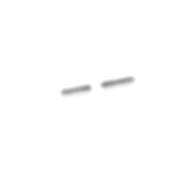

Supplement: Figure 3—figure supplement 1—source data 2. [file elife-106587-fig3-figsupp1-data2.zip › Figure 3-figure supplement 1 - source data 2/Figure 3-figure supplement 1B - source data 2/Figure 3-figure supplement 1B - S6.tif]

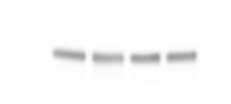

Supplement: Figure 3—figure supplement 1—source data 2. [file elife-106587-fig3-figsupp1-data2.zip › Figure 3-figure supplement 1 - source data 2/Figure 3-figure supplement 1B - source data 2/Figure 3-figure supplement 1B - AMPK.tif]

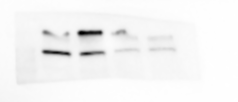

Supplement: Figure 3—figure supplement 1—source data 2. [file elife-106587-fig3-figsupp1-data2.zip › Figure 3-figure supplement 1 - source data 2/Figure 3-figure supplement 1B - source data 2/Figure 3-figure supplement 1B - ACC.tif]

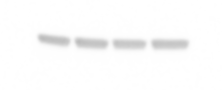

Supplement: Figure 3—figure supplement 1—source data 2. [file elife-106587-fig3-figsupp1-data2.zip › Figure 3-figure supplement 1 - source data 2/Figure 3-figure supplement 1B - source data 2/Figure 3-figure supplement 1B - B_Actin.tif]

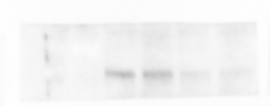

Supplement: Figure 3—figure supplement 1—source data 2. [file elife-106587-fig3-figsupp1-data2.zip › Figure 3-figure supplement 1 - source data 2/Figure 3-figure supplement 1B - source data 2/Figure 3-figure supplement 1B - pAMPK.tif]

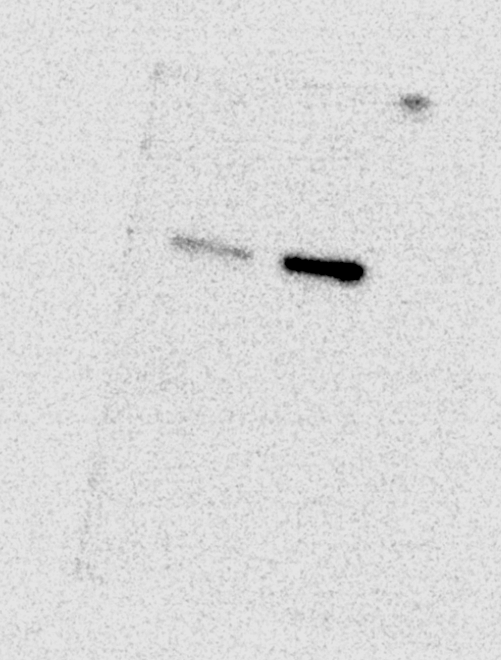

Supplement: Figure 3—figure supplement 1—source data 2. [file elife-106587-fig3-figsupp1-data2.zip › Figure 3-figure supplement 1 - source data 2/Figure 3-figure supplement 1B - source data 2/Figure 3-figure supplement 1B - pS6.tif]

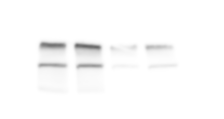

Supplement: Figure 3—figure supplement 1—source data 2. [file elife-106587-fig3-figsupp1-data2.zip › Figure 3-figure supplement 1 - source data 2/Figure 3-figure supplement 1B - source data 2/Figure 3-figure supplement 1B - pACC.tif]

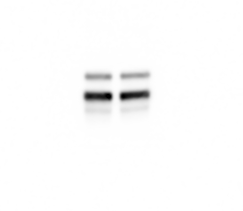

Supplement: Figure 3—figure supplement 1—source data 2. [file elife-106587-fig3-figsupp1-data2.zip › Figure 3-figure supplement 1 - source data 2/Figure 3-figure supplement 1B - source data 2/Figure 3-figure supplement 1B - S6K.tif]

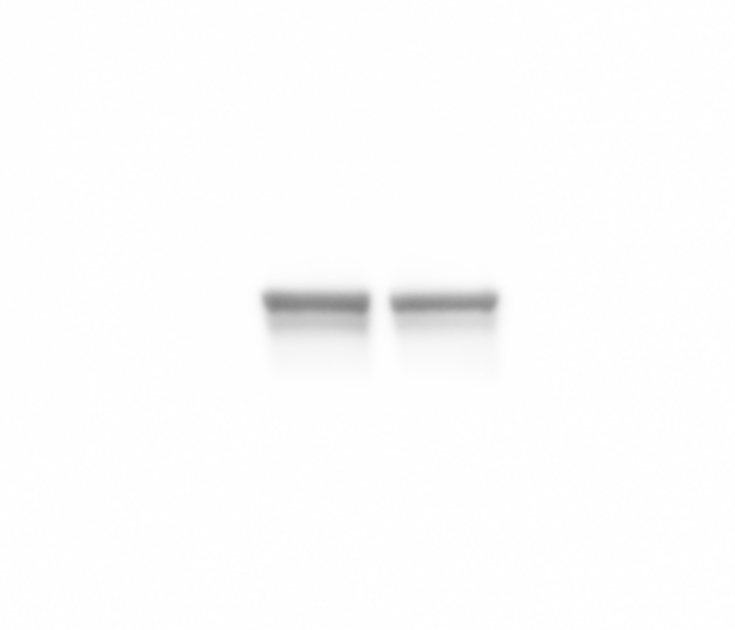

Supplement: Figure 3—figure supplement 1—source data 2. [file elife-106587-fig3-figsupp1-data2.zip › Figure 3-figure supplement 1 - source data 2/Figure 3-figure supplement 1B - source data 2/Figure 3-figure supplement 1B - AKT.tif]

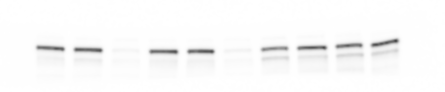

Supplement: Figure 3—figure supplement 1—source data 2. [file elife-106587-fig3-figsupp1-data2.zip › Figure 3-figure supplement 1 - source data 2/Figure 3-figure supplement 1D - source data 2/Figure 3-figure supplement 1D - RICTOR.tif]

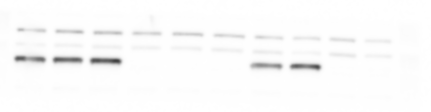

Supplement: Figure 3—figure supplement 1—source data 2. [file elife-106587-fig3-figsupp1-data2.zip › Figure 3-figure supplement 1 - source data 2/Figure 3-figure supplement 1D - source data 2/Figure 3-figure supplement 1D - ETFDH.tif]

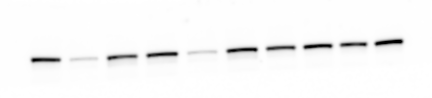

Supplement: Figure 3—figure supplement 1—source data 2. [file elife-106587-fig3-figsupp1-data2.zip › Figure 3-figure supplement 1 - source data 2/Figure 3-figure supplement 1D - source data 2/Figure 3-figure supplement 1D - RAPTOR.tif]

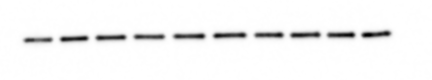

Supplement: Figure 3—figure supplement 1—source data 2. [file elife-106587-fig3-figsupp1-data2.zip › Figure 3-figure supplement 1 - source data 2/Figure 3-figure supplement 1D - source data 2/Figure 3-figure supplement 1D - B_actin.tif]

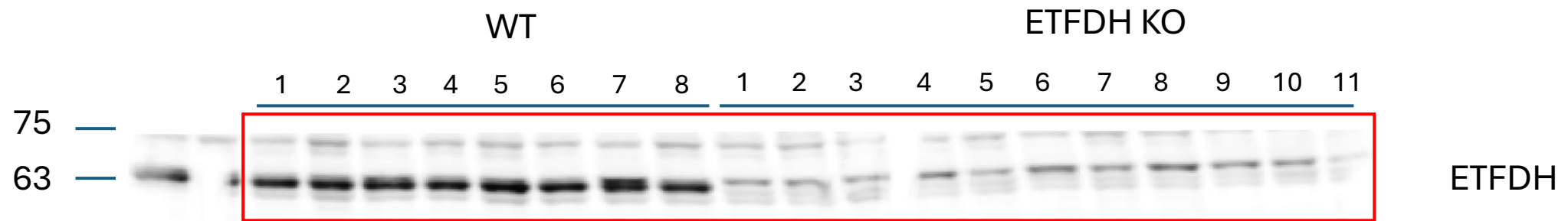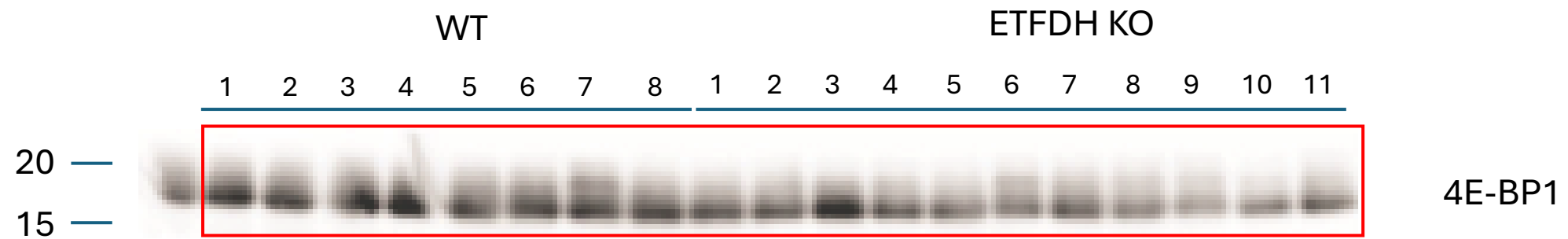

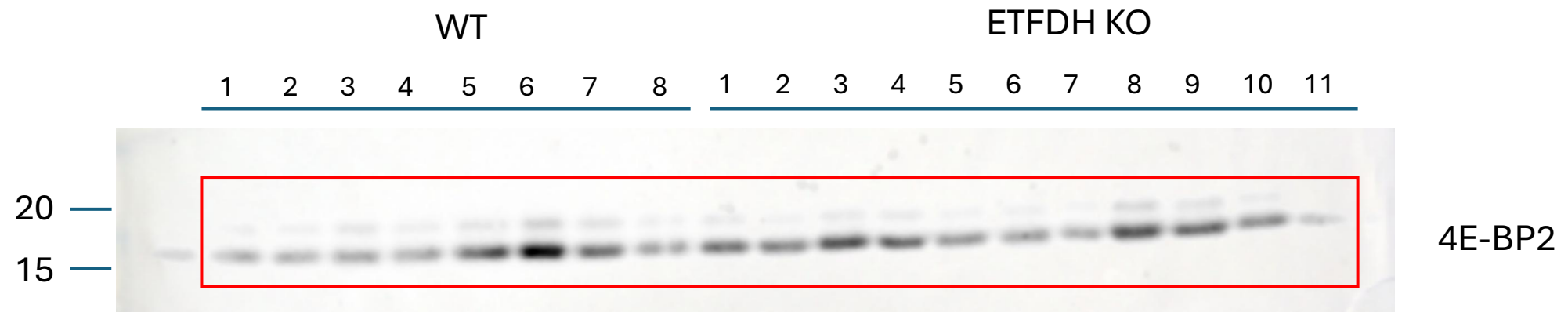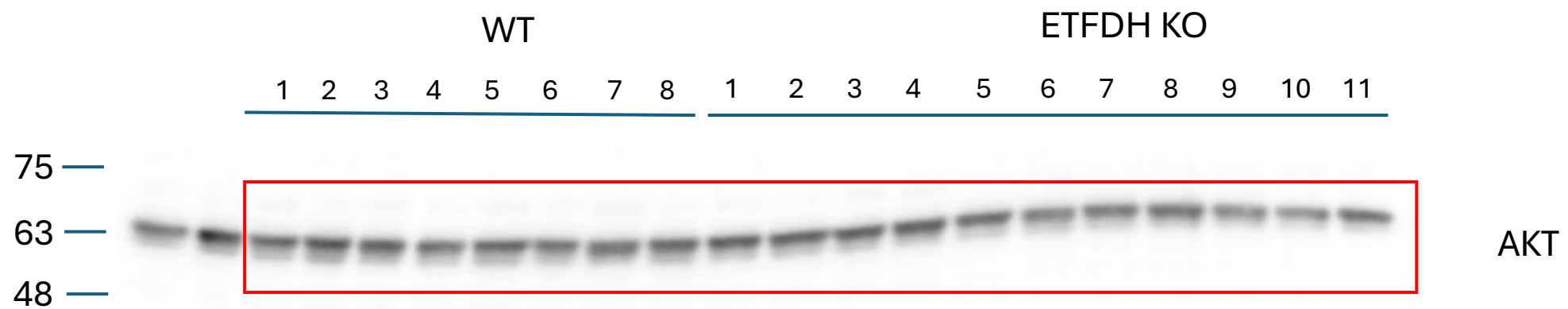

Supplement: Figure 4—source data 1. [file elife-106587-fig4-data1.zip › Figure 4 - source data 1/Figure 4C - source data 1/Figure 4C - source data 1.pdf]

HCT-116

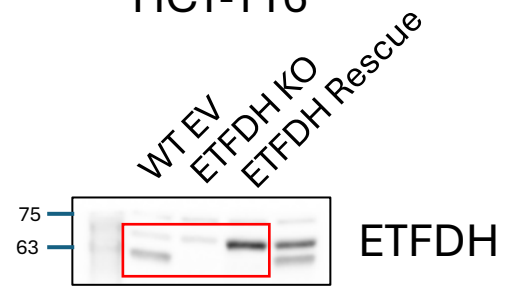

HCT-116

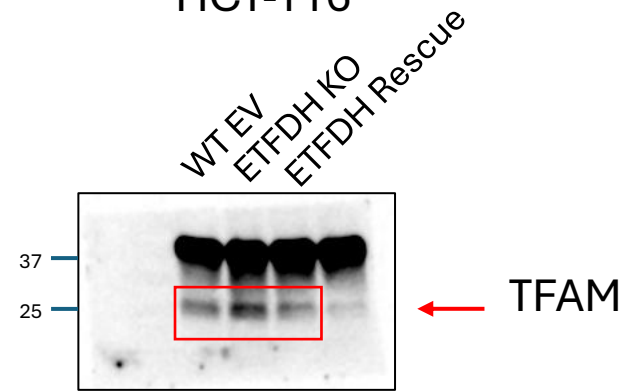

HCT-116

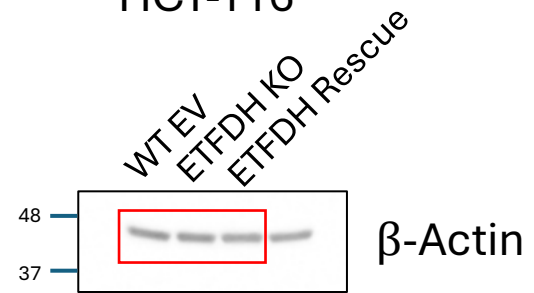

Supplement: Figure 4—source data 1. [file elife-106587-fig4-data1.zip › Figure 4 - source data 1/Figure 4K - source data 1/Figure 4K - source data 1.pdf]

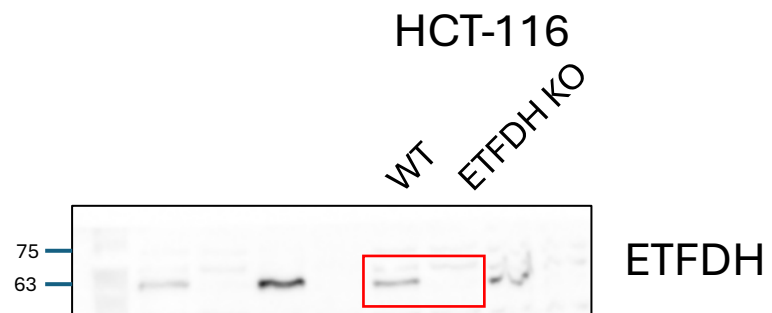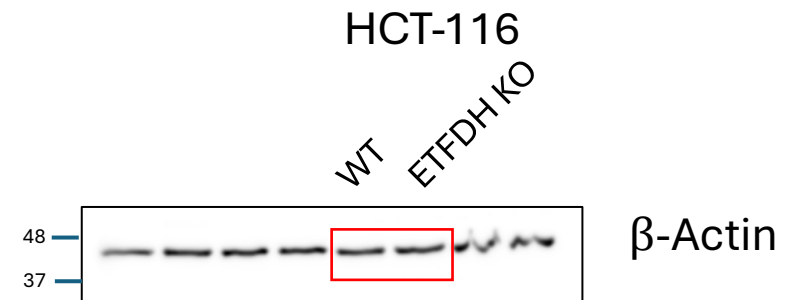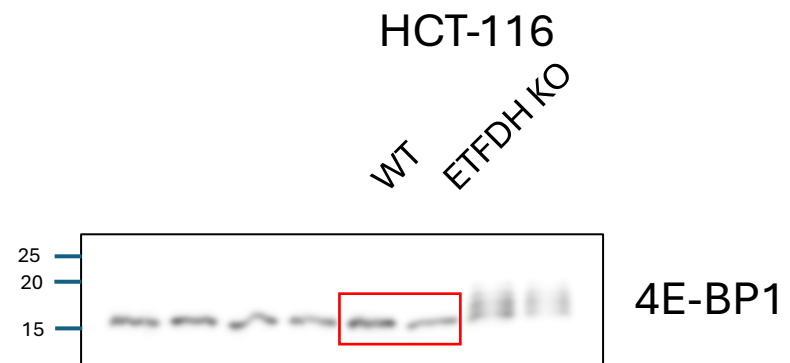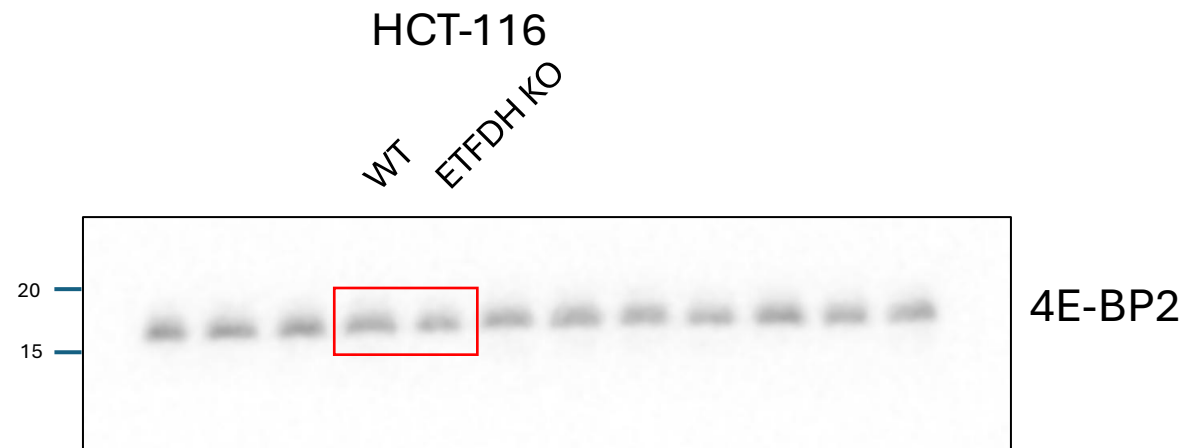

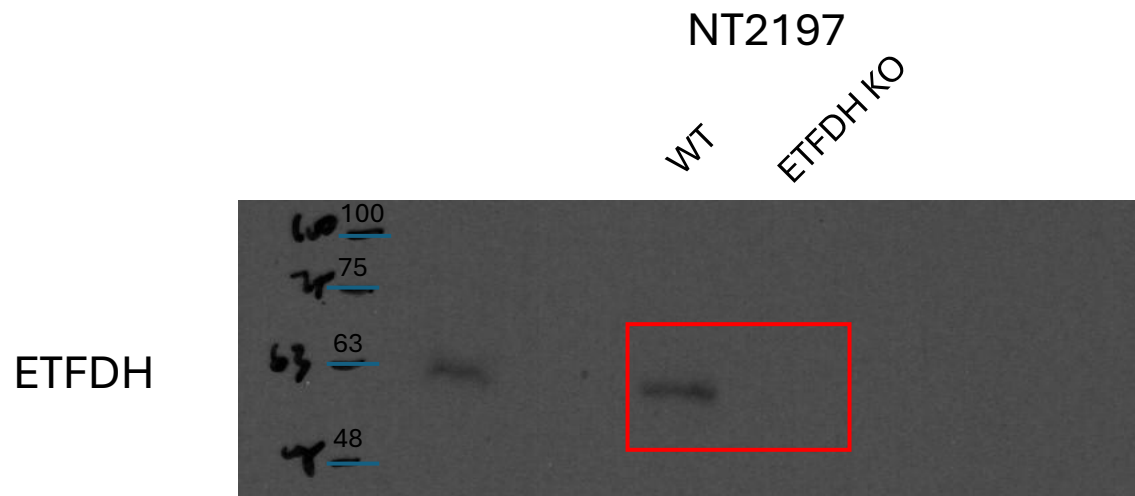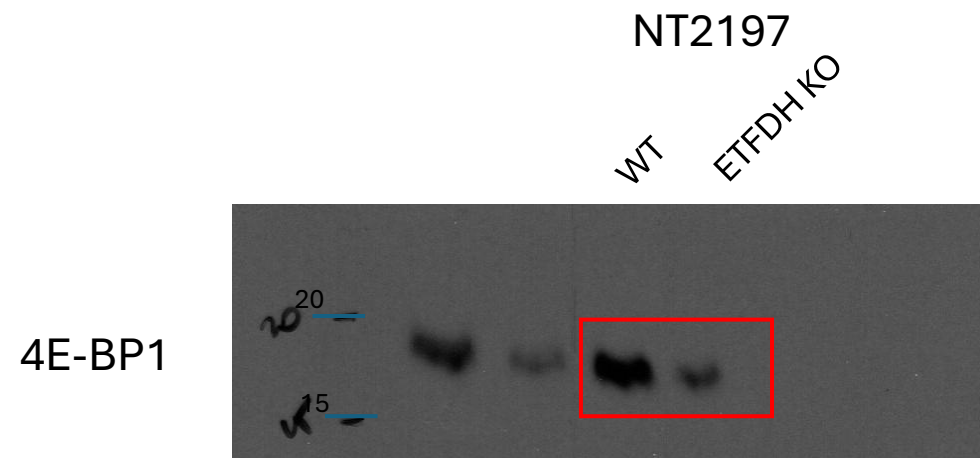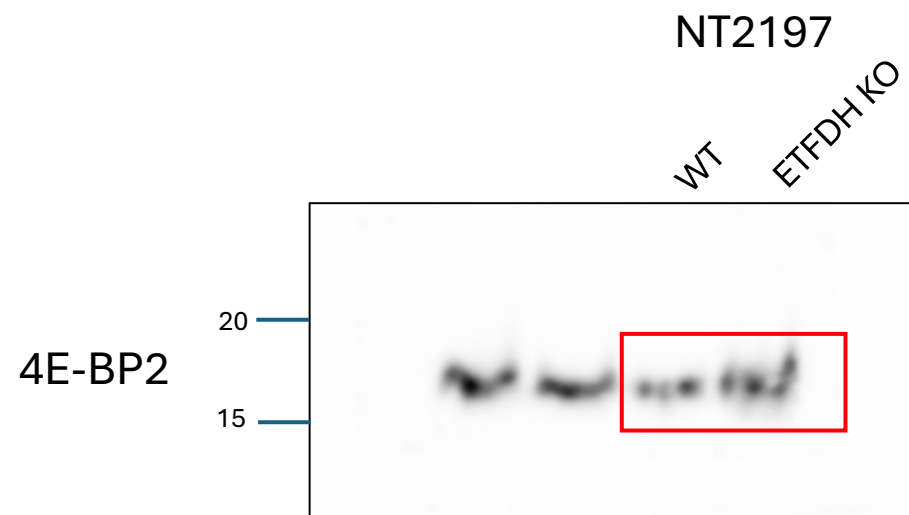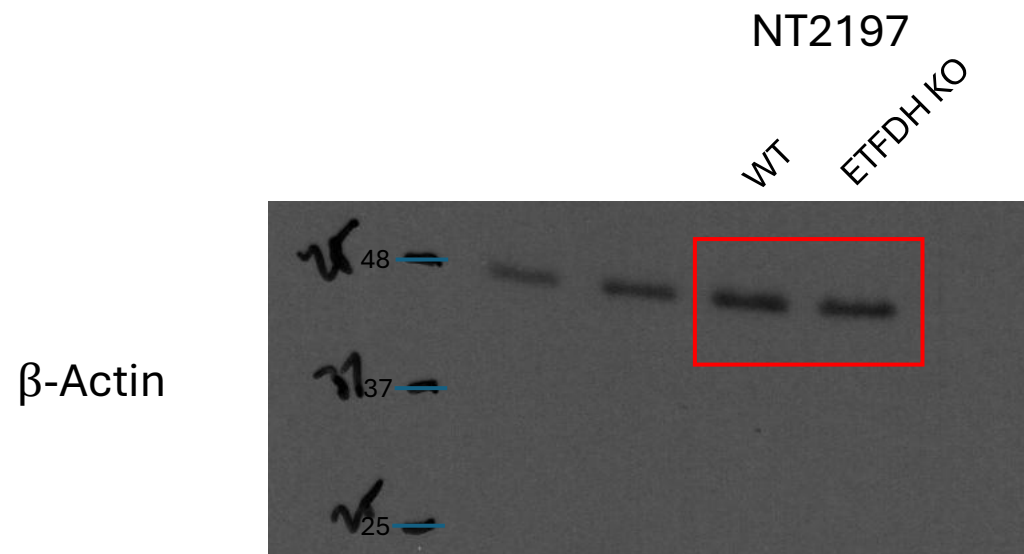

Supplement: Figure 4—source data 1. [file elife-106587-fig4-data1.zip › Figure 4 - source data 1/Figure 4B - source data 1/Figure 4B - source data 1.pdf]

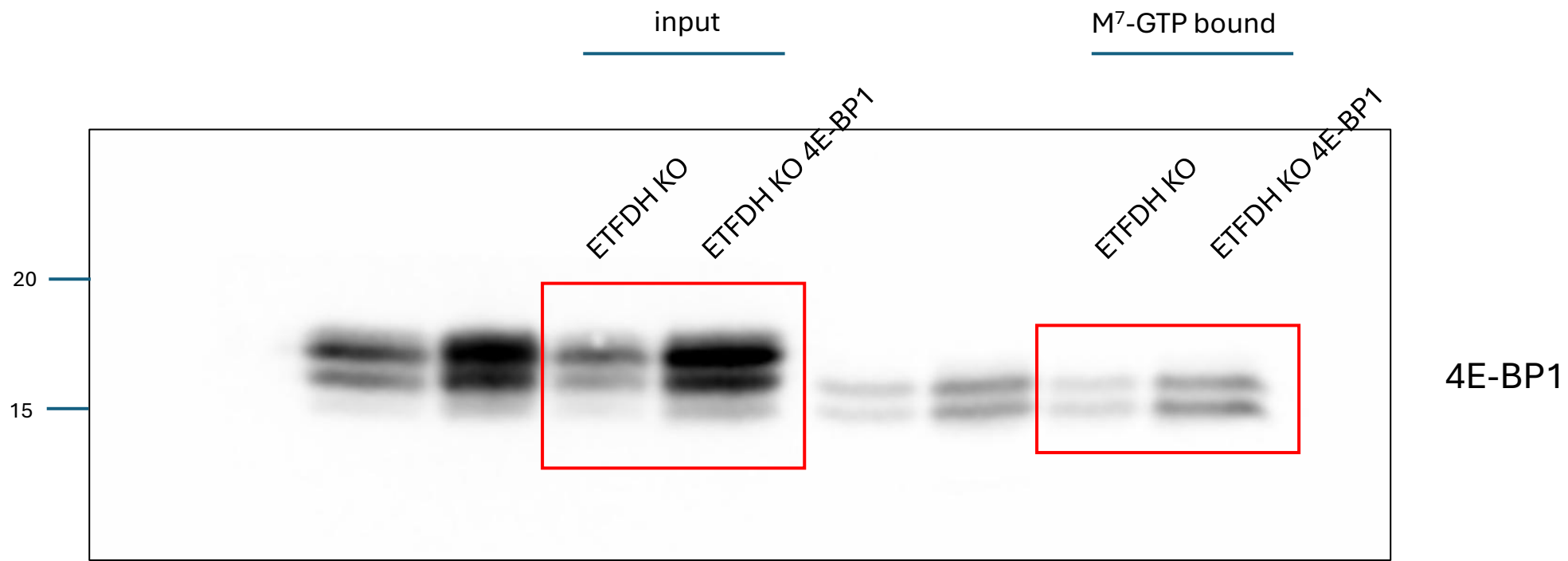

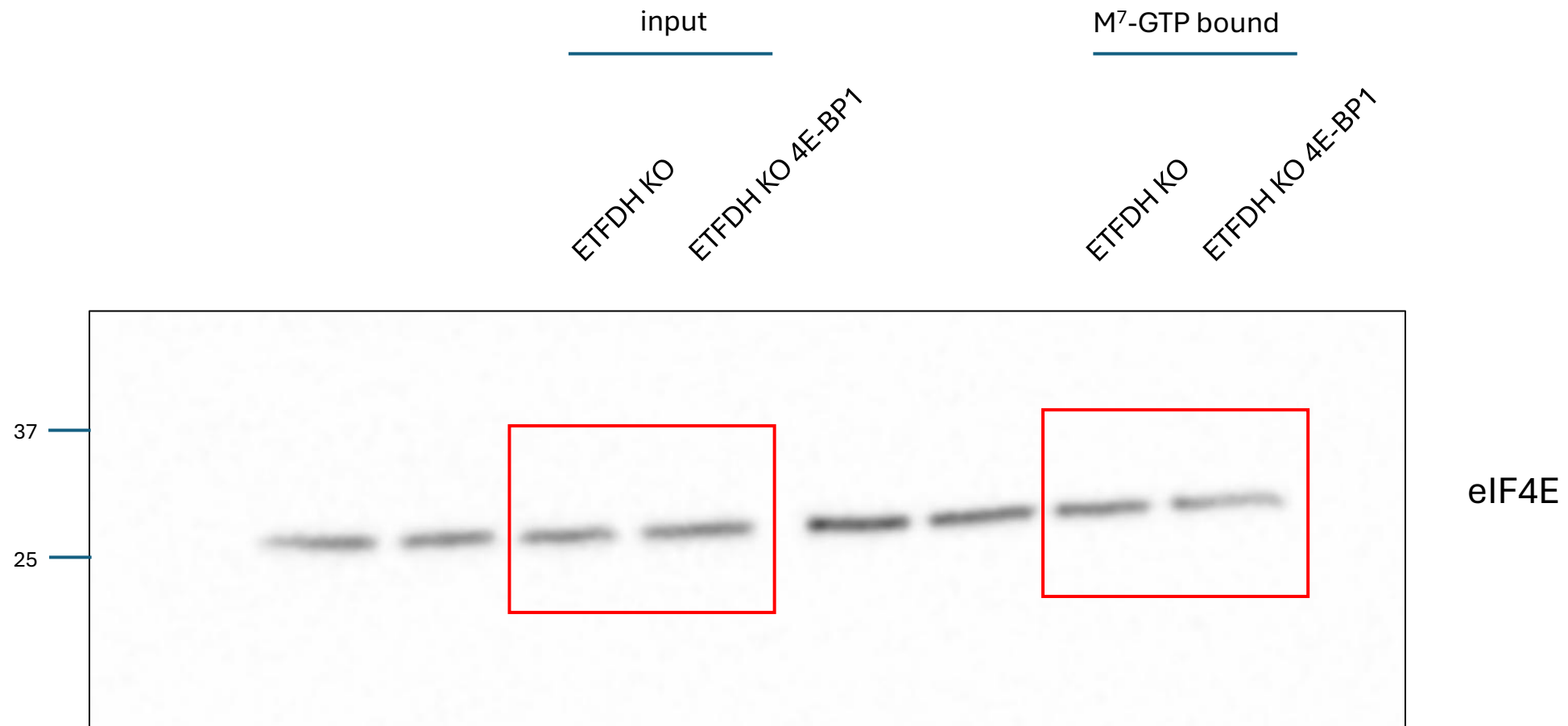

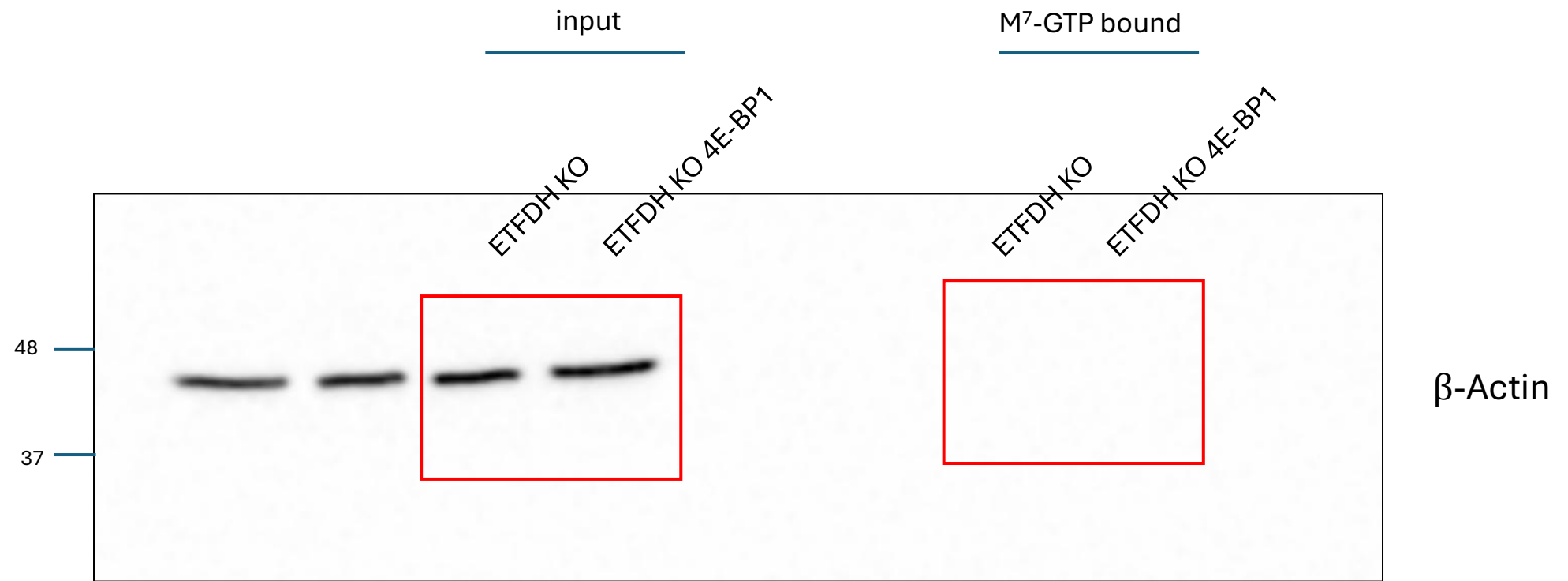

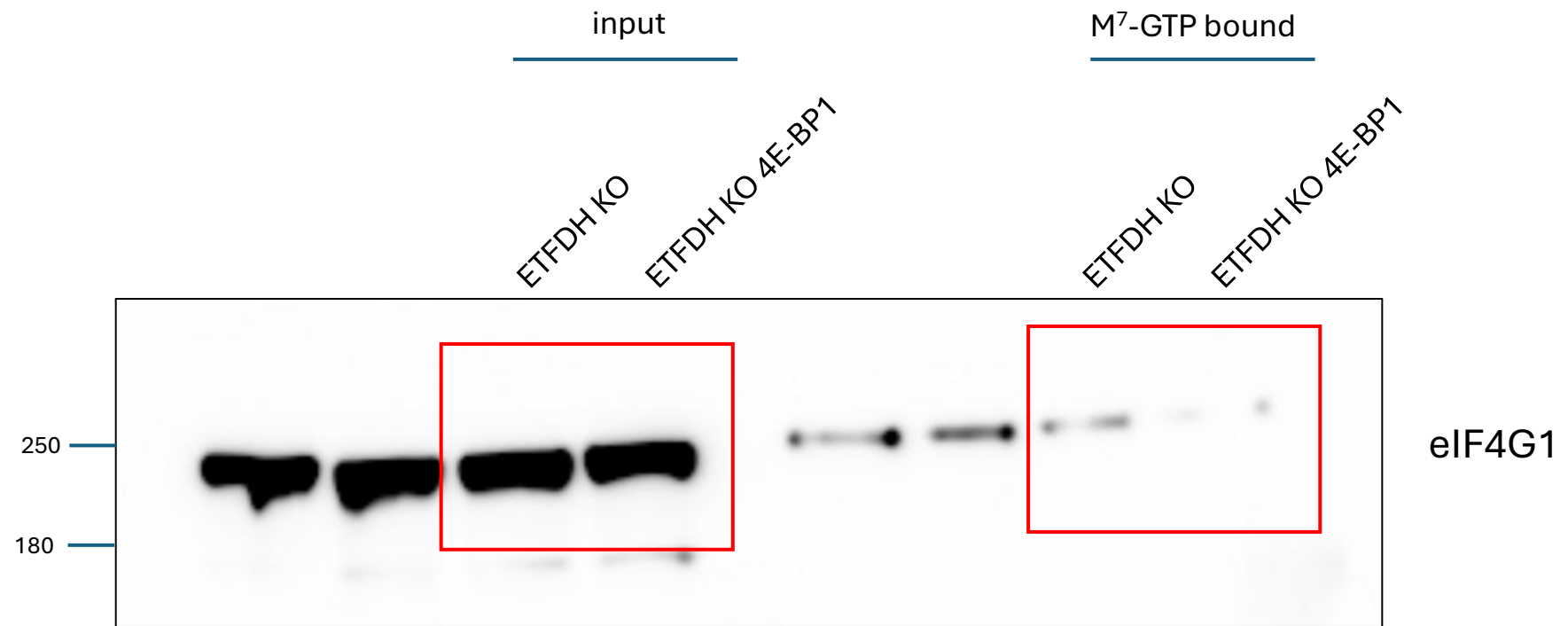

Supplement: Figure 4—source data 1. [file elife-106587-fig4-data1.zip › Figure 4 - source data 1/Figure 4F - source data 1/Figure 4F - source data 1.pdf]

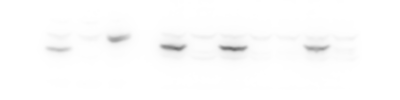

Supplement: Figure 4—source data 2. [file elife-106587-fig4-data2.zip › Figure 4 - source data 2/Figure 4A - source data 2/Figure 4A - NT2197 ETFDH.tif]

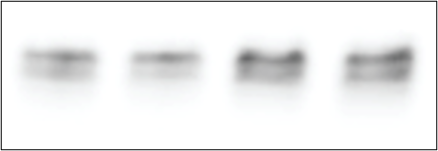

Supplement: Figure 4—source data 2. [file elife-106587-fig4-data2.zip › Figure 4 - source data 2/Figure 4A - source data 2/Figure 4A - NT2197 p4E-BP1.tif]

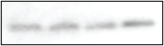

Supplement: Figure 4—source data 2. [file elife-106587-fig4-data2.zip › Figure 4 - source data 2/Figure 4A - source data 2/Figure 4A - HCT-116 4E-BP2.tif]

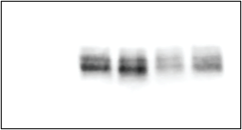

Supplement: Figure 4—source data 2. [file elife-106587-fig4-data2.zip › Figure 4 - source data 2/Figure 4A - source data 2/Figure 4A - HCT-116 4E-BP1.tif]

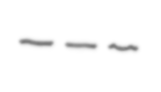

Supplement: Figure 4—source data 2. [file elife-106587-fig4-data2.zip › Figure 4 - source data 2/Figure 4A - source data 2/Figure 4A - NT2197 B_Actin.tif]

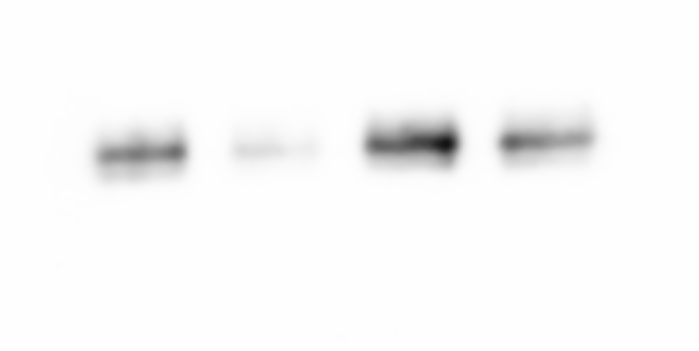

Supplement: Figure 4—source data 2. [file elife-106587-fig4-data2.zip › Figure 4 - source data 2/Figure 4A - source data 2/Figure 4A - NT2197 4E-BP1.tif]

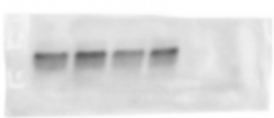

Supplement: Figure 4—source data 2. [file elife-106587-fig4-data2.zip › Figure 4 - source data 2/Figure 4A - source data 2/Figure 4A - HCT-116 p4E-BP1.tif]

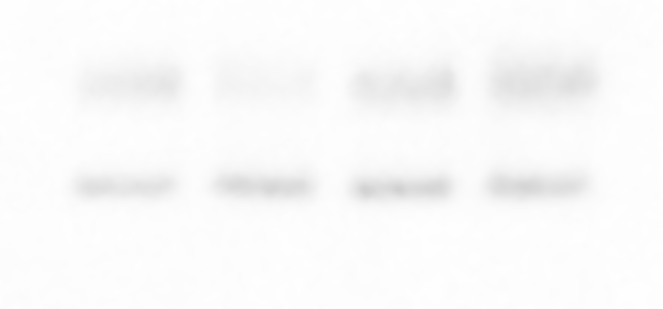

Supplement: Figure 4—source data 2. [file elife-106587-fig4-data2.zip › Figure 4 - source data 2/Figure 4A - source data 2/Figure 4A - NT2197 4E-BP2.tif]

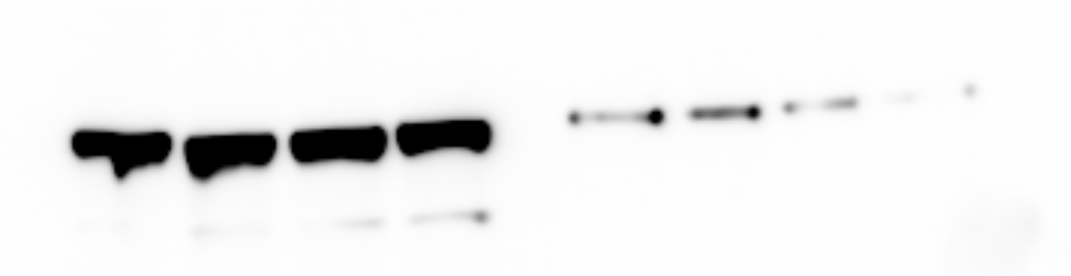

Supplement: Figure 4—source data 2. [file elife-106587-fig4-data2.zip › Figure 4 - source data 2/Figure 4F - source data 2/Figure 4F - eIF4G1.tif]

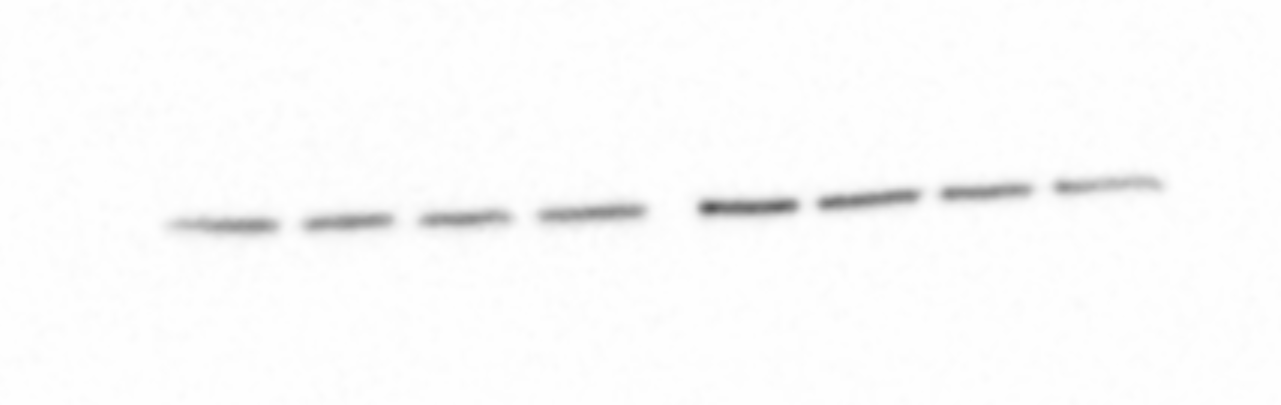

Supplement: Figure 4—source data 2. [file elife-106587-fig4-data2.zip › Figure 4 - source data 2/Figure 4F - source data 2/Figure 4F - eIF4E.tif]

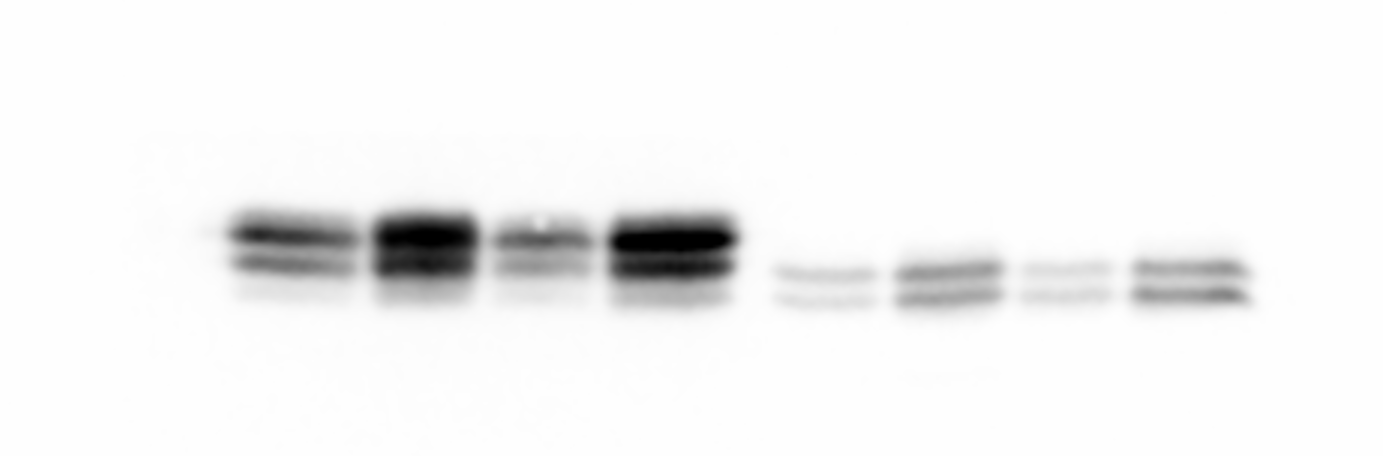

Supplement: Figure 4—source data 2. [file elife-106587-fig4-data2.zip › Figure 4 - source data 2/Figure 4F - source data 2/Figure 4F - 4E-BP1.tif]

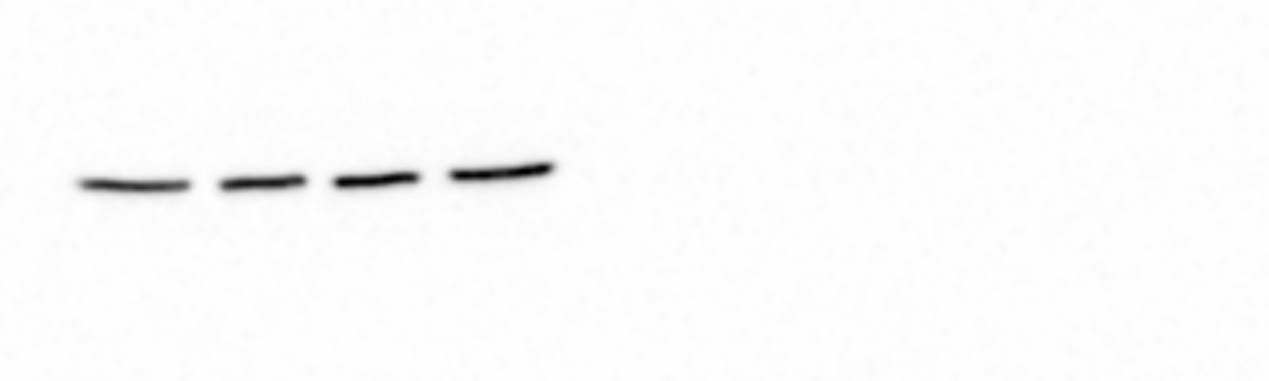

Supplement: Figure 4—source data 2. [file elife-106587-fig4-data2.zip › Figure 4 - source data 2/Figure 4F - source data 2/Figure 4F - B_Actin.tif]

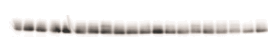

Supplement: Figure 4—source data 2. [file elife-106587-fig4-data2.zip › Figure 4 - source data 2/Figure 4C - source data 2/Figure 4 - 4E-BP1.tiff]

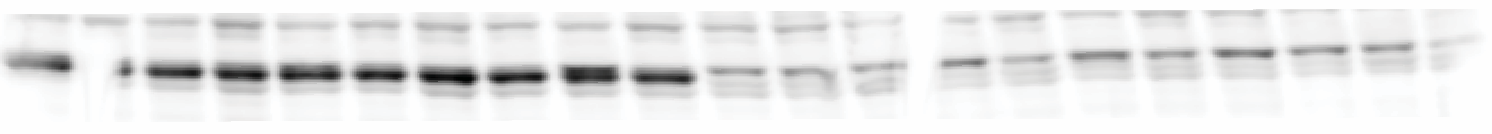

Supplement: Figure 4—source data 2. [file elife-106587-fig4-data2.zip › Figure 4 - source data 2/Figure 4C - source data 2/Figure 4C - ETFDH.tif]

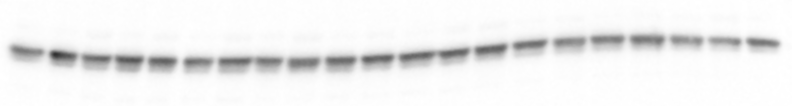

Supplement: Figure 4—source data 2. [file elife-106587-fig4-data2.zip › Figure 4 - source data 2/Figure 4C - source data 2/Figure 4 - AKT.tif]

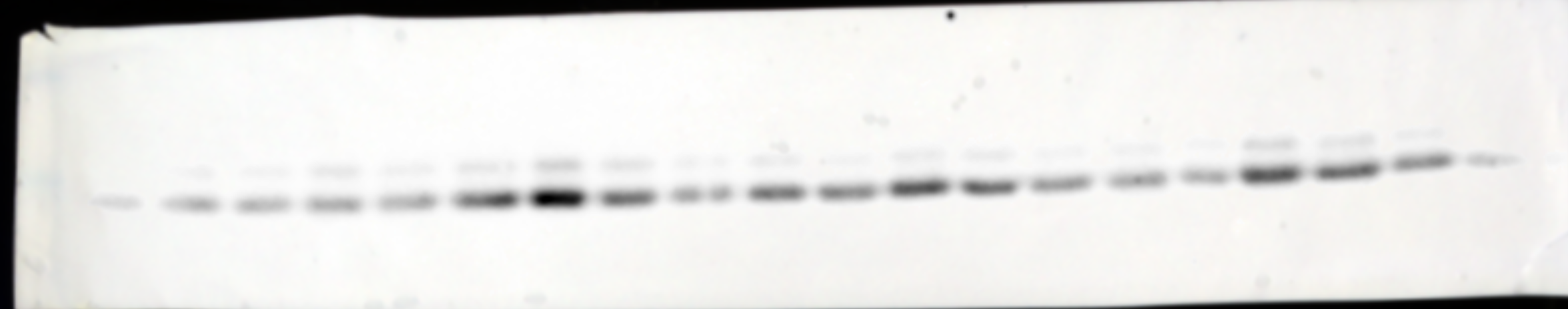

Supplement: Figure 4—source data 2. [file elife-106587-fig4-data2.zip › Figure 4 - source data 2/Figure 4C - source data 2/Figure 4 - 4E-BP2.tif]

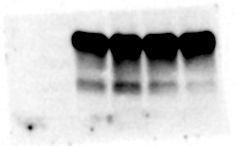

Supplement: Figure 4—source data 2. [file elife-106587-fig4-data2.zip › Figure 4 - source data 2/Figure 4K - source data 2/Figure 4K - TFAM.tif]

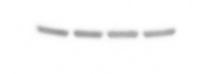

Supplement: Figure 4—source data 2. [file elife-106587-fig4-data2.zip › Figure 4 - source data 2/Figure 4K - source data 2/Figure 4K - B_Actin.tif]
