# Supplementary material for: Mitochondrial ETF insufficiency drives neoplastic growth by selectively optimizing cancer bioenergetics
Source: eLife. 2026 May 5;14:RP106587. doi: 10.7554/eLife.106587 (PMC13143275; doi:10.7554/eLife.106587)
Supplement: Figure 3—source data 1. [file elife-106587-fig3-data1.zip › Figure 3 - source data 1/Figure 3C - source data 1/Figure 3C - source data 1.pdf]

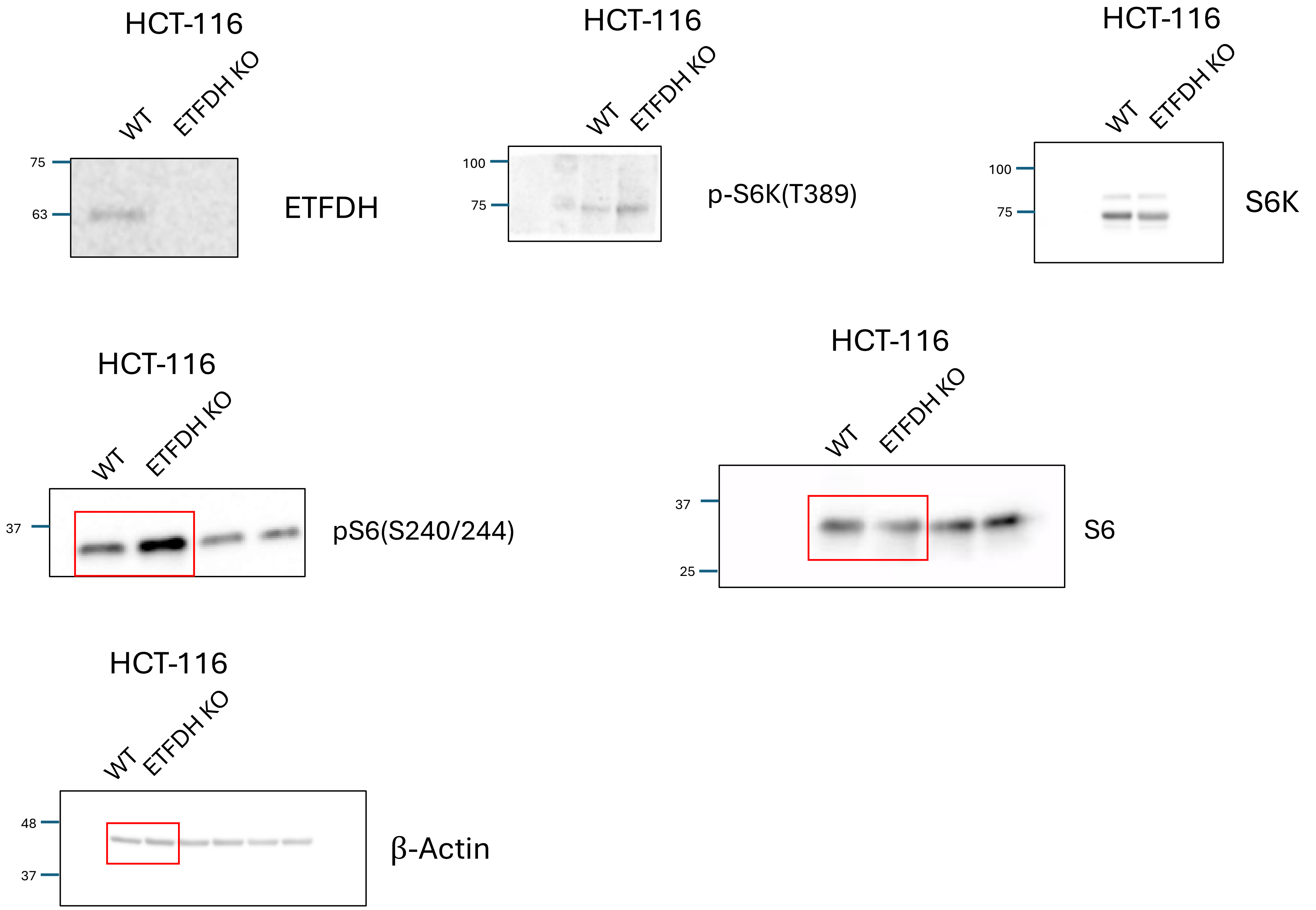
HCT-116
WT
ETFDH KO
75
63
ETFDH
HCT-116
WT
ETFDH KO
100
75
p-S6K(T389)
HCT-116
WT
ETFDH KO
100
75
S6K
HCT-116
WT
ETFDH KO
37
pS6(S240/244)
HCT-116
WT
ETFDH KO
37
25
S6
HCT-116
WT
ETFDH KO
48
37
β-Actin

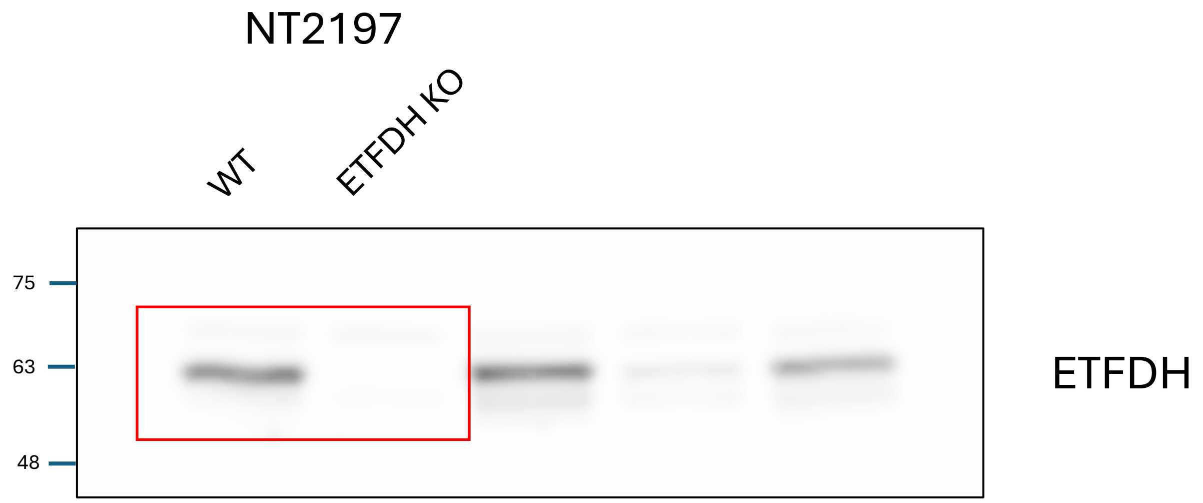
NT2197
WT
ETFDH KO
75
63
48
ETFDH

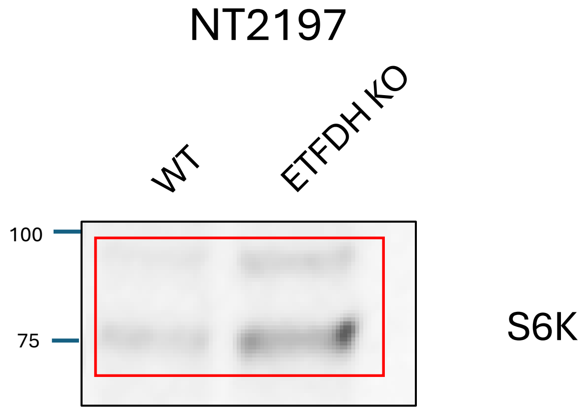
NT2197
WT
ETFDH KO
100
75
S6K

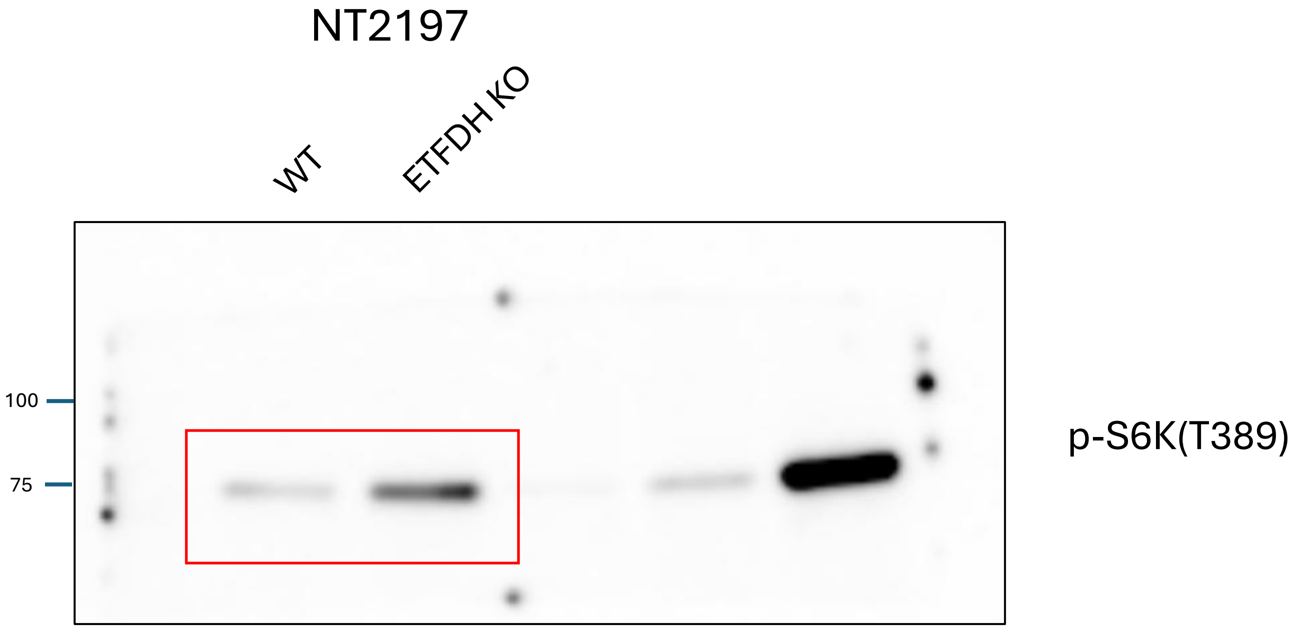
NT2197
WT
ETFDH KO
100
75
p-S6K(T389)

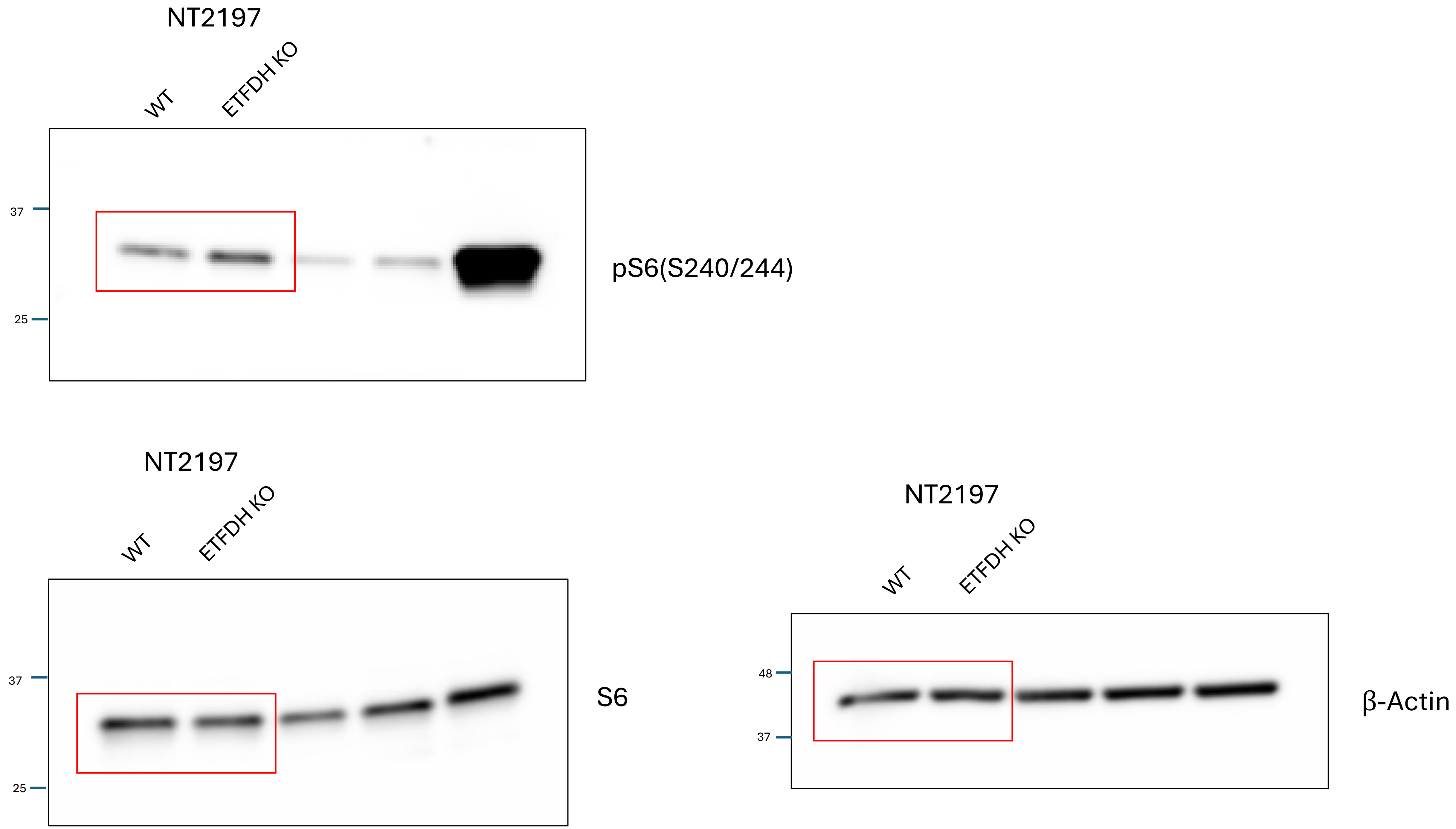
NT2197
WT
ETFDH KO
37
25
pS6(S240/244)
NT2197
WT
ETFDH KO
37
25
S6
NT2197
WT
ETFDH KO
48
37
β-Actin
