# Supplementary material for: Mitochondrial ETF insufficiency drives neoplastic growth by selectively optimizing cancer bioenergetics
Source: eLife. 2026 May 5;14:RP106587. doi: 10.7554/eLife.106587 (PMC13143275; doi:10.7554/eLife.106587)
Supplement: Figure 4—source data 1. [file elife-106587-fig4-data1.zip › Figure 4 - source data 1/Figure 4A - source data 1/Figure 4A - source data 1.pdf]

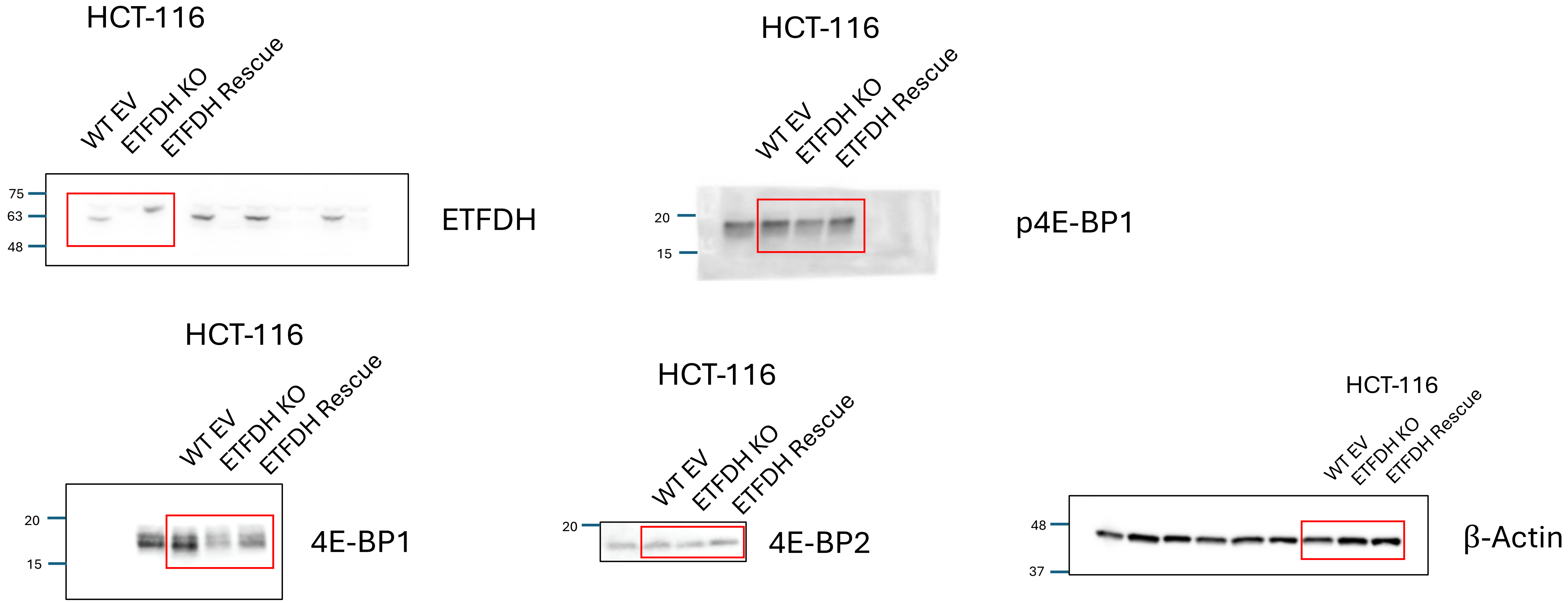
HCT-116
WT EV
ETFDH KO
ETFDH Rescue
75
63
48
ETFDH
HCT-116
WT EV
ETFDH KO
ETFDH Rescue
20
15
p4E-BP1
HCT-116
WT EV
ETFDH KO
ETFDH Rescue
20
15
4E-BP1
HCT-116
WT EV
ETFDH KO
ETFDH Rescue
20
4E-BP2
HCT-116
WT EV
ETFDH KO
ETFDH Rescue
48
37
β-Actin

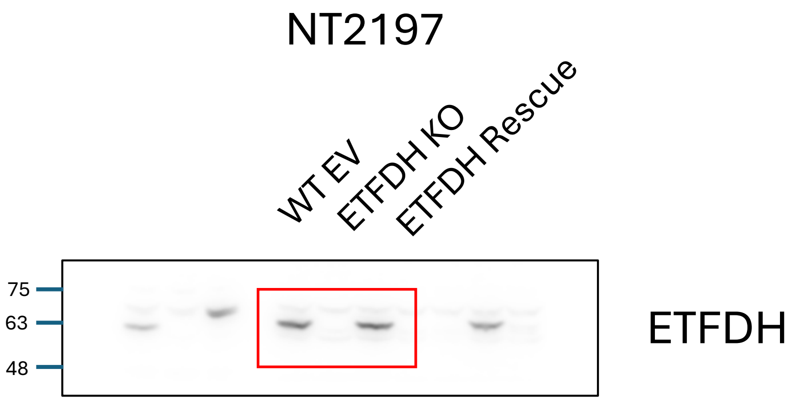
NT2197
WT EV
ETFDH KO
ETFDH Rescue
75
63
48
ETFDH

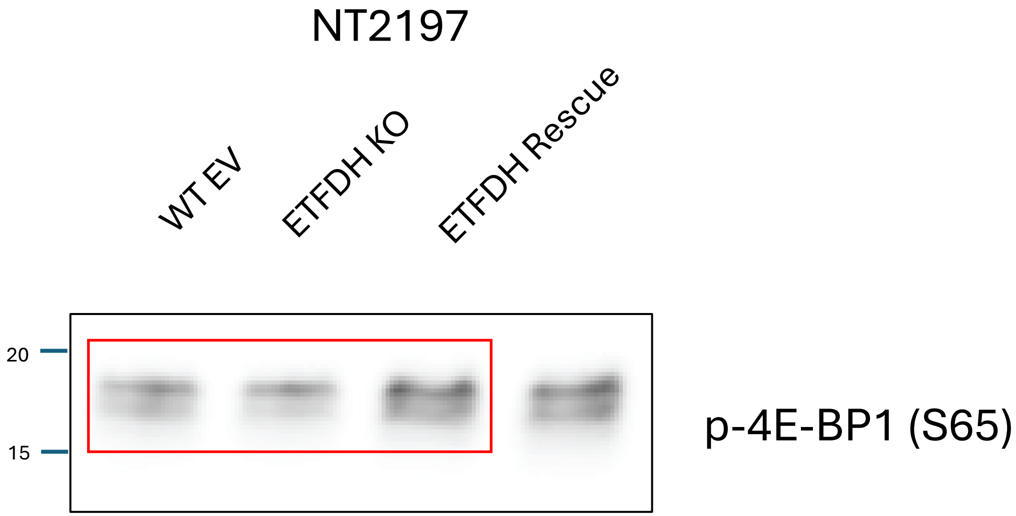
NT2197
WT EV
ETFDH KO
ETFDH Rescue
20
15
p-4E-BP1 (S65)

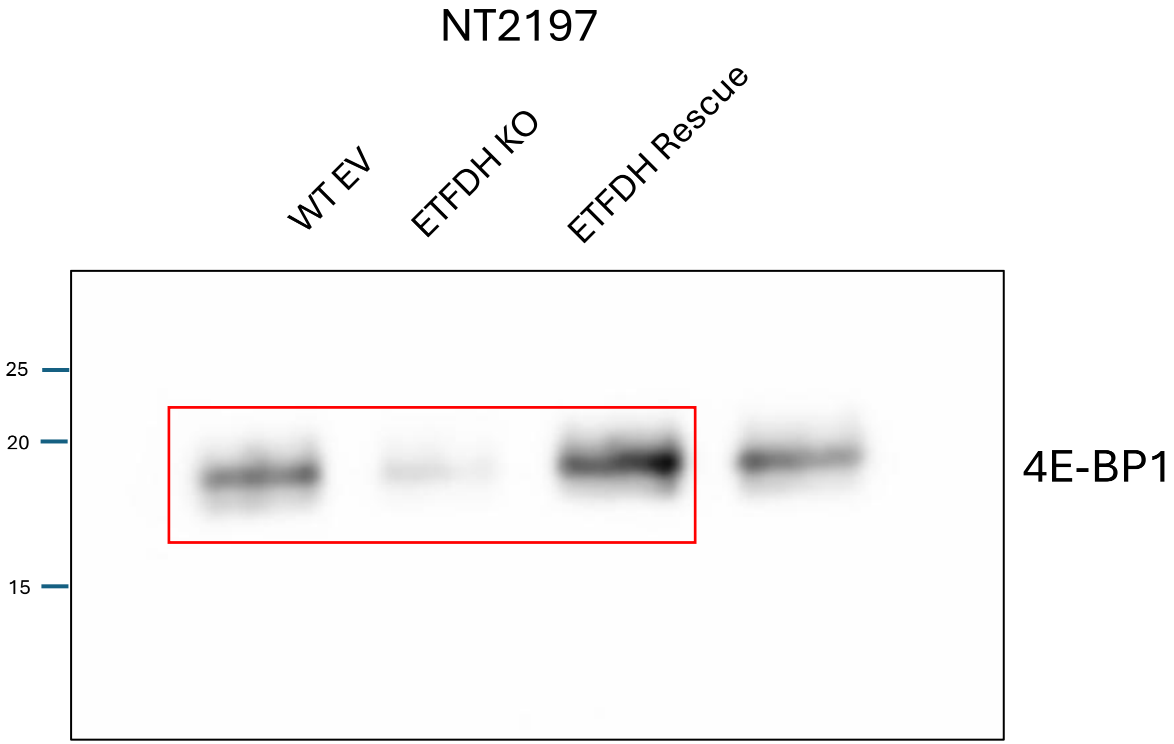
NT2197
WT EV
ETFDH KO
ETFDH Rescue
25
20
15
4E-BP1

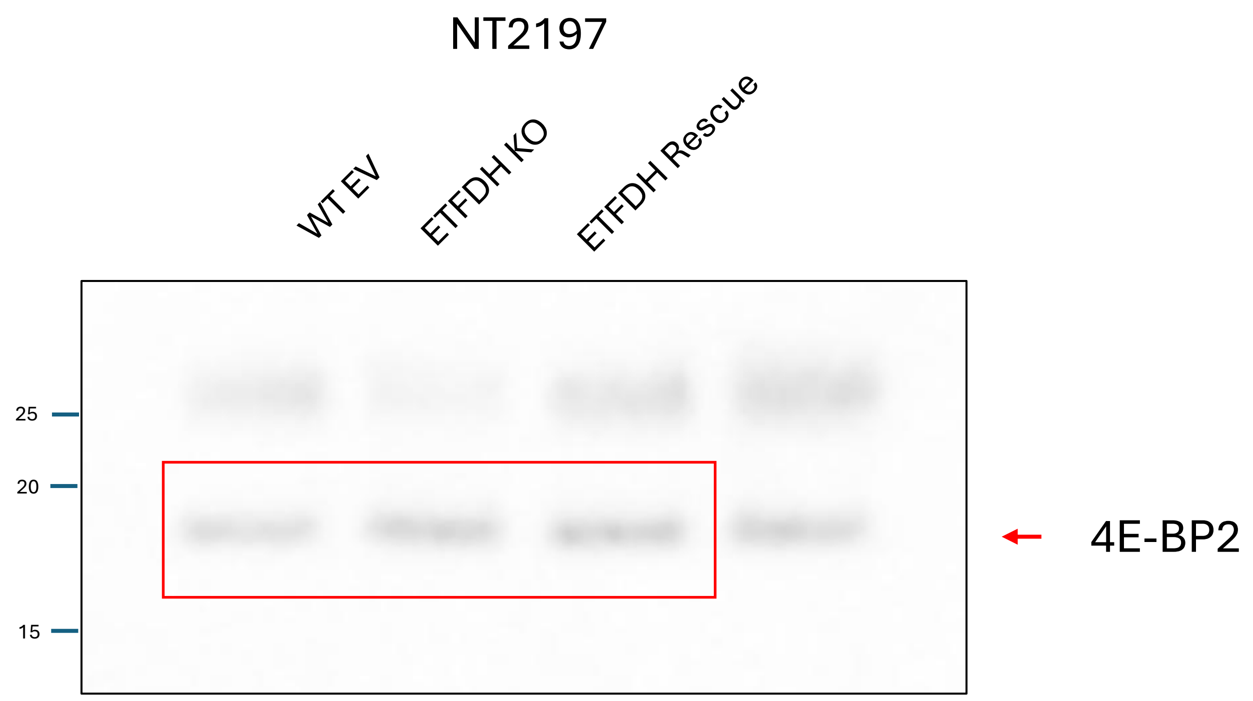
NT2197
WT EV
ETFDH KO
ETFDH Rescue
25
20
15
4E-BP2

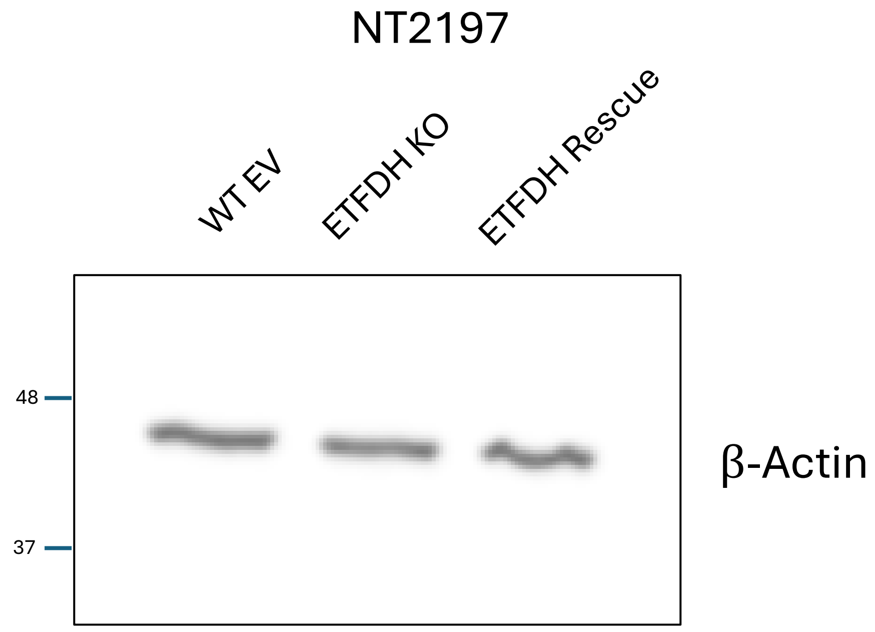
NT2197
WT EV
ETFDH KO
ETFDH Rescue
48
37
β-Actin
