# Supplementary figures and images for: Mitochondrial ETF insufficiency drives neoplastic growth by selectively optimizing cancer bioenergetics (part 2 of 2)
Source: eLife. 2026 May 5;14:RP106587. doi: 10.7554/eLife.106587 (PMC13143275; doi:10.7554/eLife.106587)

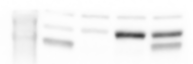

Supplement: Figure 4—source data 2. [file elife-106587-fig4-data2.zip › Figure 4 - source data 2/Figure 4K - source data 2/Figure 4K - ETFDH.tif]

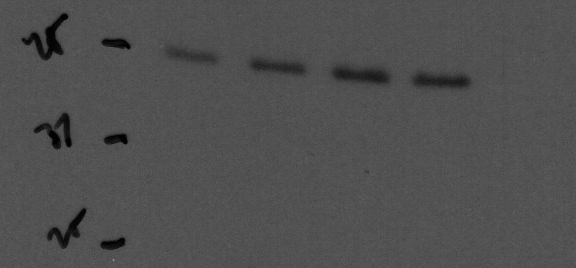

Supplement: Figure 4—source data 2. [file elife-106587-fig4-data2.zip › Figure 4 - source data 2/Figure 4B - source data 2/Figure 4B - NT2197 B_Actin.tif]

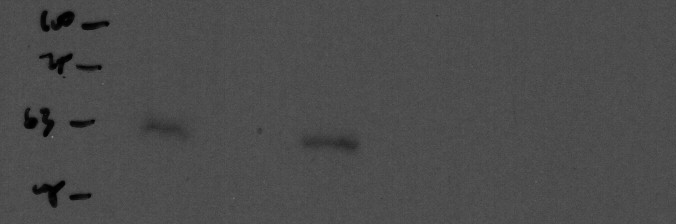

Supplement: Figure 4—source data 2. [file elife-106587-fig4-data2.zip › Figure 4 - source data 2/Figure 4B - source data 2/Figure 4B - NT2197 ETFDH.tif]

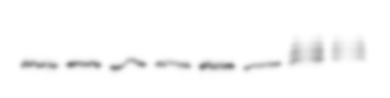

Supplement: Figure 4—source data 2. [file elife-106587-fig4-data2.zip › Figure 4 - source data 2/Figure 4B - source data 2/Figure 4B - HCT-116 4E-BP1.tif]

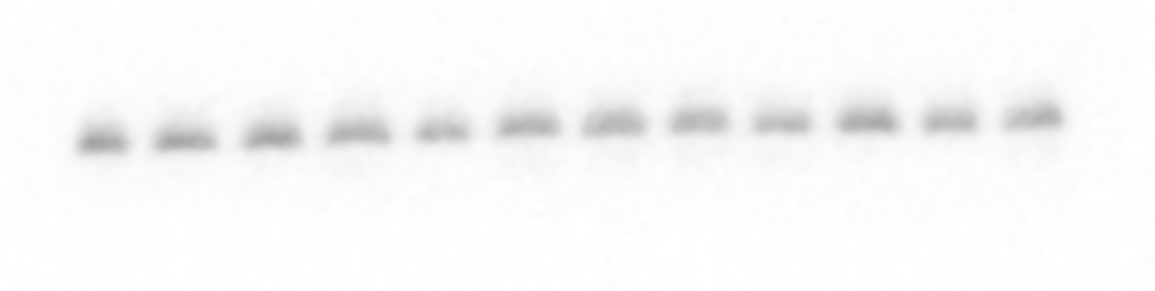

Supplement: Figure 4—source data 2. [file elife-106587-fig4-data2.zip › Figure 4 - source data 2/Figure 4B - source data 2/Figure 4B - HCT-116 4E-BP2.tif]

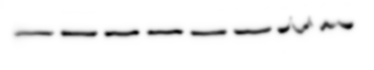

Supplement: Figure 4—source data 2. [file elife-106587-fig4-data2.zip › Figure 4 - source data 2/Figure 4B - source data 2/Figure 4B - HCT-116 B_Actin.tif]

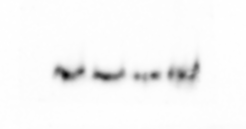

Supplement: Figure 4—source data 2. [file elife-106587-fig4-data2.zip › Figure 4 - source data 2/Figure 4B - source data 2/Figure 4B - NT2197 4E-BP2.tif]

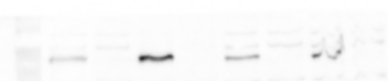

Supplement: Figure 4—source data 2. [file elife-106587-fig4-data2.zip › Figure 4 - source data 2/Figure 4B - source data 2/Figure 4B - HCT-116 ETFDH.tif]

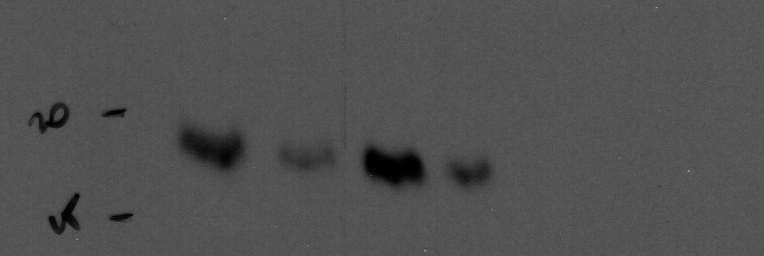

Supplement: Figure 4—source data 2. [file elife-106587-fig4-data2.zip › Figure 4 - source data 2/Figure 4B - source data 2/Figure 4B - NT2197 4E-BP1.tif]

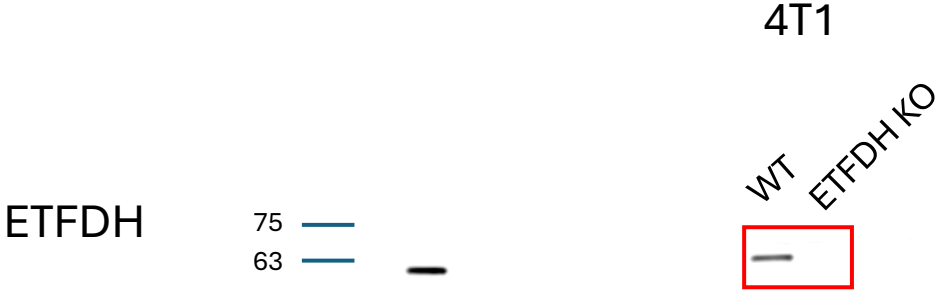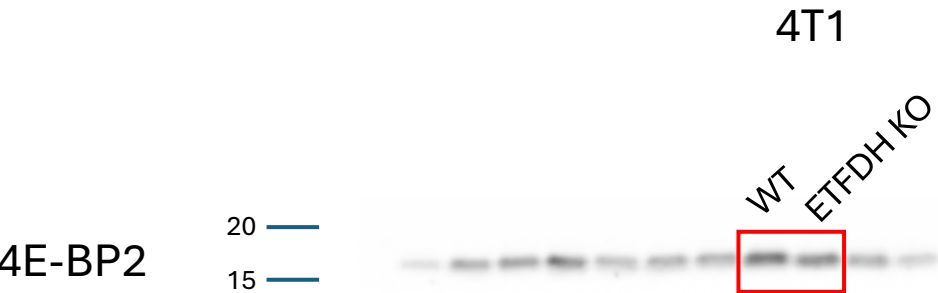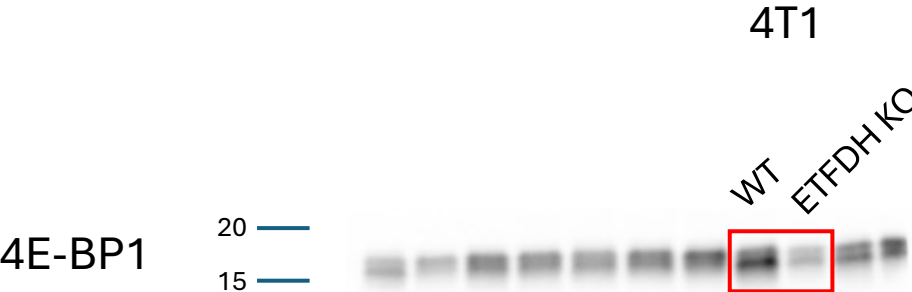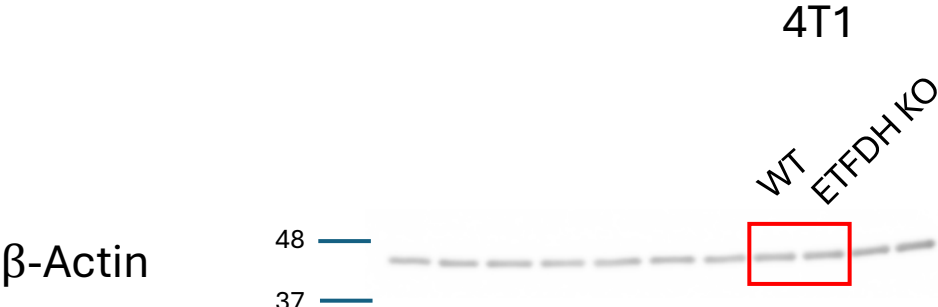

Supplement: Figure 4—figure supplement 1—source data 1. [file elife-106587-fig4-figsupp1-data1.zip › Figure 4-figure supplement 1 - source data 1/Figure 4-figure supplement 1B - source data 1/Figure 4-figure supplement 1B - source data 1.pdf]

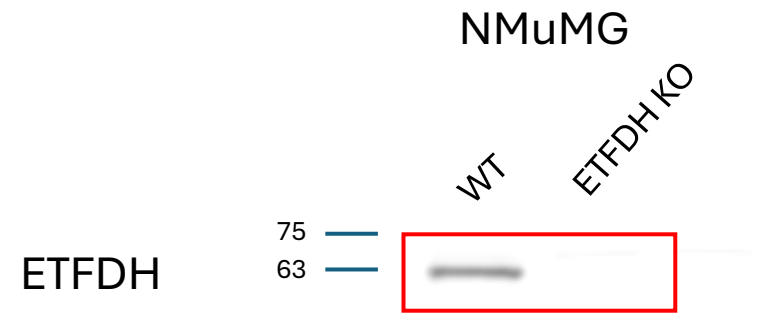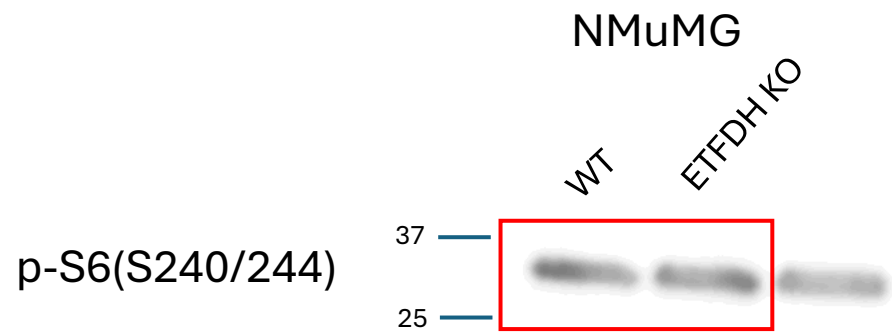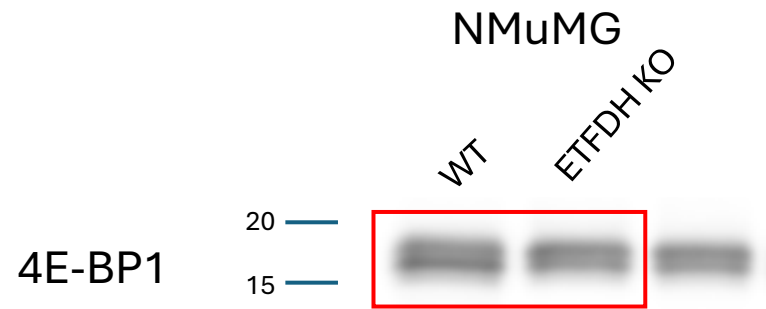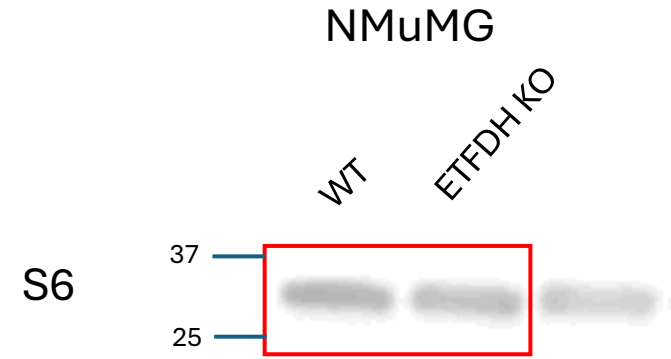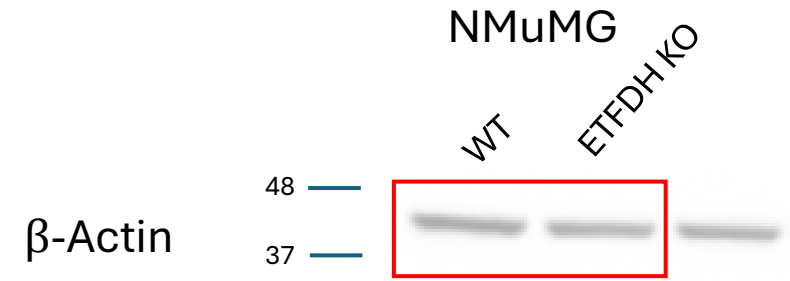

Supplement: Figure 4—figure supplement 1—source data 1. [file elife-106587-fig4-figsupp1-data1.zip › Figure 4-figure supplement 1 - source data 1/Figure 4-figure supplement 1C - source data 1/Figure 4-figure supplement 1C - source data 1.pdf]

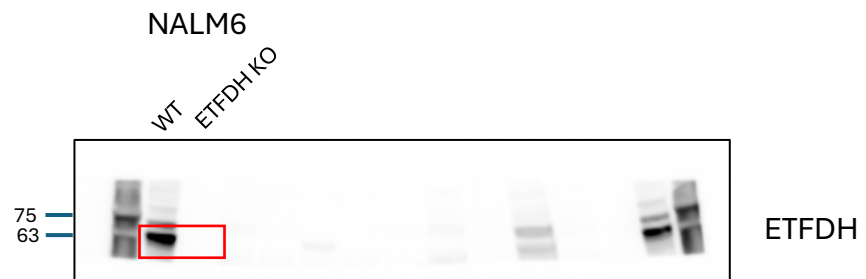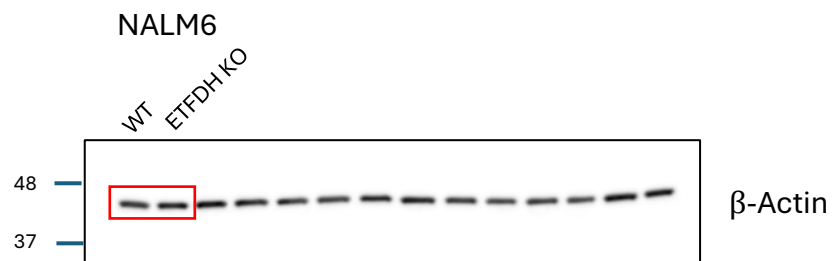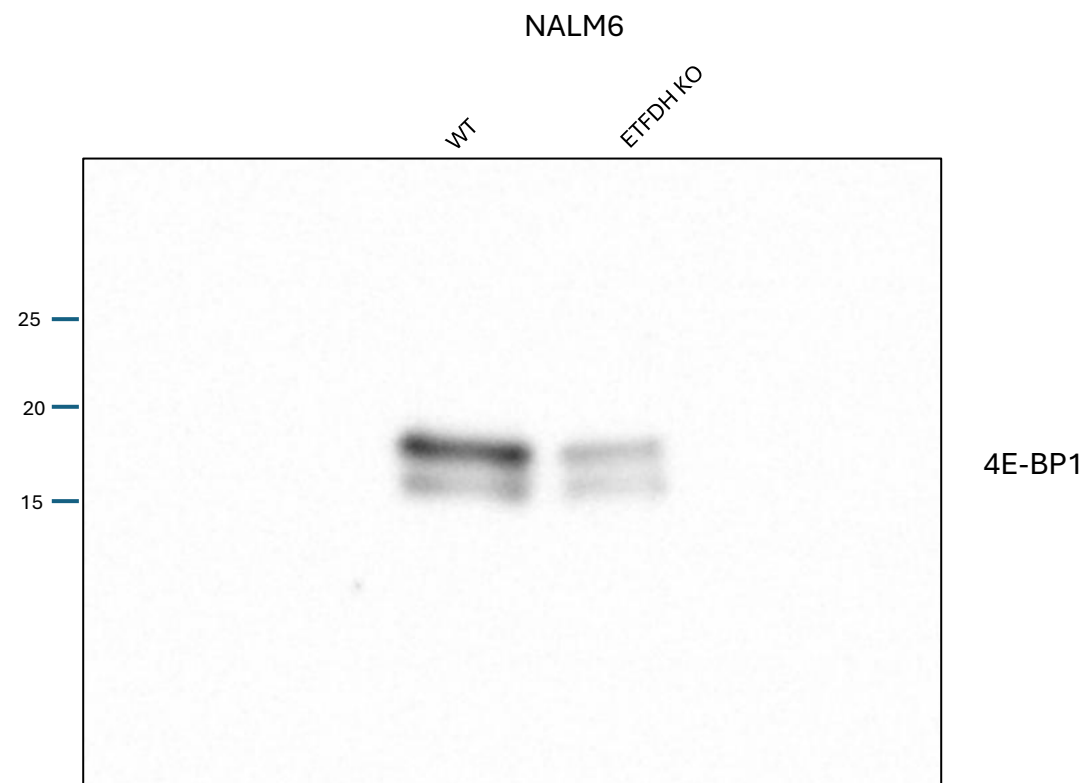

Supplement: Figure 4—figure supplement 1—source data 1. [file elife-106587-fig4-figsupp1-data1.zip › Figure 4-figure supplement 1 - source data 1/Figure 4-figure supplement 1A - source data 1/Figure 4-figure supplement 1A - source data 1.pdf]

## HCT-116

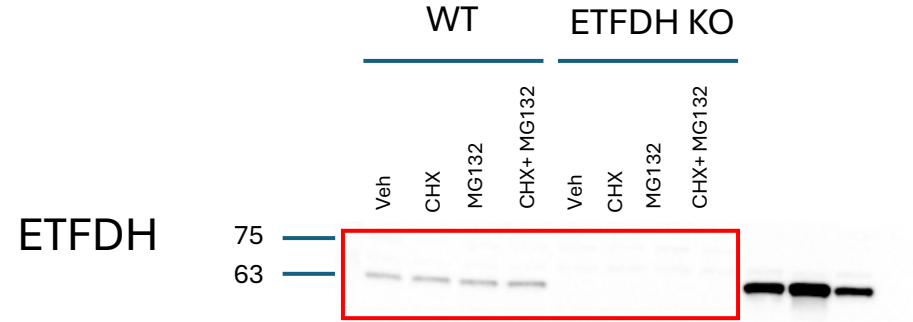

## HCT-116

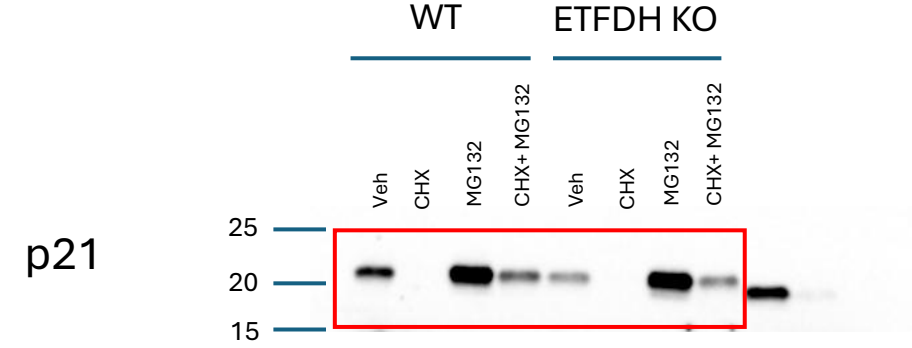

## HCT-116

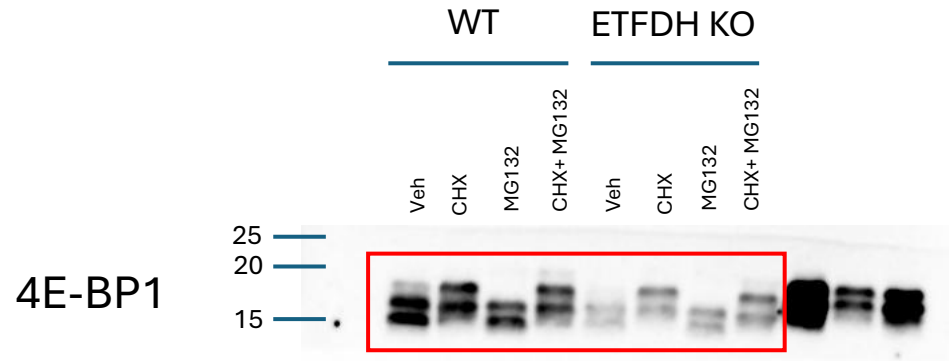

## HCT-116

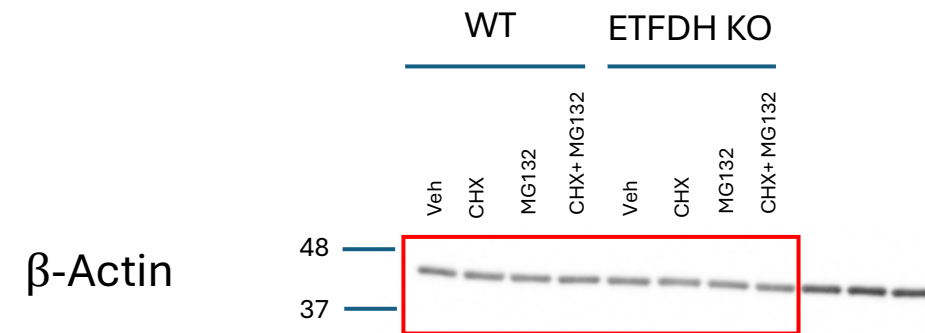

Supplement: Figure 4—figure supplement 1—source data 1. [file elife-106587-fig4-figsupp1-data1.zip › Figure 4-figure supplement 1 - source data 1/Figure 4-figure supplement 1G - source data 1/Figure 4-figure supplement 1G - source data 1.pdf]

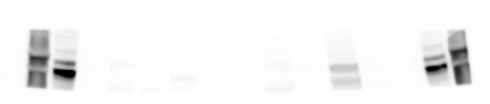

Supplement: Figure 4—figure supplement 1—source data 2. [file elife-106587-fig4-figsupp1-data2.zip › Figure 4-figure supplement 1 - source data 2/Figure 4-figure supplement 1A - source data 2/Figure 4-figure supplement 1A - ETFDH.tif]

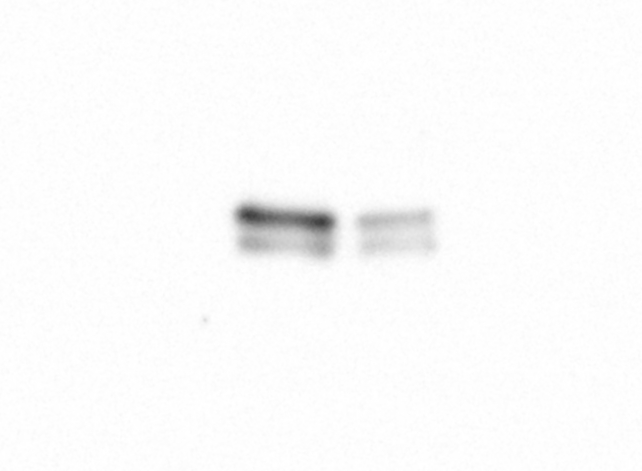

Supplement: Figure 4—figure supplement 1—source data 2. [file elife-106587-fig4-figsupp1-data2.zip › Figure 4-figure supplement 1 - source data 2/Figure 4-figure supplement 1A - source data 2/Figure 4-figure supplement 1A - 4E-BP1.tif]

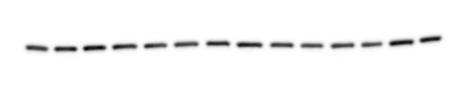

Supplement: Figure 4—figure supplement 1—source data 2. [file elife-106587-fig4-figsupp1-data2.zip › Figure 4-figure supplement 1 - source data 2/Figure 4-figure supplement 1A - source data 2/Figure 4-figure supplement 1A - B_Actin.tif]

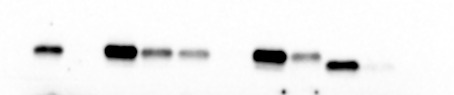

Supplement: Figure 4—figure supplement 1—source data 2. [file elife-106587-fig4-figsupp1-data2.zip › Figure 4-figure supplement 1 - source data 2/Figure 4-figure supplement 1G - source data 2/Figure 4-figure supplement 1G - p21.tif]

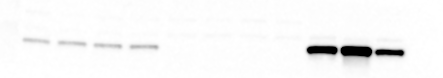

Supplement: Figure 4—figure supplement 1—source data 2. [file elife-106587-fig4-figsupp1-data2.zip › Figure 4-figure supplement 1 - source data 2/Figure 4-figure supplement 1G - source data 2/Figure 4-figure supplement 1G - ETFDH.tif]

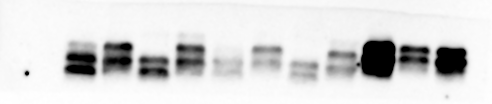

Supplement: Figure 4—figure supplement 1—source data 2. [file elife-106587-fig4-figsupp1-data2.zip › Figure 4-figure supplement 1 - source data 2/Figure 4-figure supplement 1G - source data 2/Figure 4-figure supplement 1G - 4E-BP1.tif]

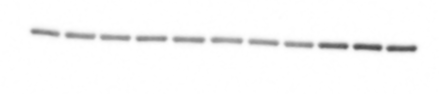

Supplement: Figure 4—figure supplement 1—source data 2. [file elife-106587-fig4-figsupp1-data2.zip › Figure 4-figure supplement 1 - source data 2/Figure 4-figure supplement 1G - source data 2/Figure 4-figure supplement 1G - B_Actin.tif]

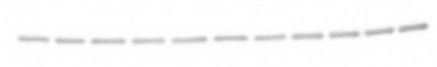

Supplement: Figure 4—figure supplement 1—source data 2. [file elife-106587-fig4-figsupp1-data2.zip › Figure 4-figure supplement 1 - source data 2/Figure 4-figure supplement 1B - source data 2/Figure 4-figure supplement 1B - B_Actin.tif]

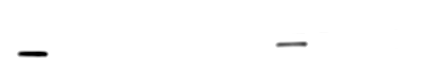

Supplement: Figure 4—figure supplement 1—source data 2. [file elife-106587-fig4-figsupp1-data2.zip › Figure 4-figure supplement 1 - source data 2/Figure 4-figure supplement 1B - source data 2/Figure 4-figure supplement 1B - ETFDH.tif]

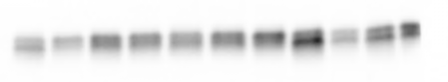

Supplement: Figure 4—figure supplement 1—source data 2. [file elife-106587-fig4-figsupp1-data2.zip › Figure 4-figure supplement 1 - source data 2/Figure 4-figure supplement 1B - source data 2/Figure 4-figure supplement 1B - 4E-BP1.tif]

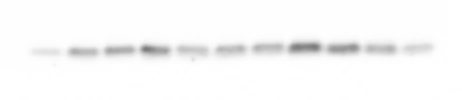

Supplement: Figure 4—figure supplement 1—source data 2. [file elife-106587-fig4-figsupp1-data2.zip › Figure 4-figure supplement 1 - source data 2/Figure 4-figure supplement 1B - source data 2/Figure 4-figure supplement 1B - 4E-BP2.tif]

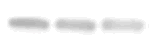

Supplement: Figure 4—figure supplement 1—source data 2. [file elife-106587-fig4-figsupp1-data2.zip › Figure 4-figure supplement 1 - source data 2/Figure 4-figure supplement 1C - source data 2/Figure 4-figure supplement 1C - S6.tif]

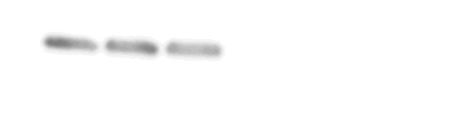

Supplement: Figure 4—figure supplement 1—source data 2. [file elife-106587-fig4-figsupp1-data2.zip › Figure 4-figure supplement 1 - source data 2/Figure 4-figure supplement 1C - source data 2/Figure 4-figure supplement 1C - pS6.tif]

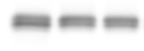

Supplement: Figure 4—figure supplement 1—source data 2. [file elife-106587-fig4-figsupp1-data2.zip › Figure 4-figure supplement 1 - source data 2/Figure 4-figure supplement 1C - source data 2/Figure 4-figure supplement 1C - 4E-BP1.tif]

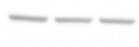

Supplement: Figure 4—figure supplement 1—source data 2. [file elife-106587-fig4-figsupp1-data2.zip › Figure 4-figure supplement 1 - source data 2/Figure 4-figure supplement 1C - source data 2/Figure 4-figure supplement 1C - B_Actin.tif]

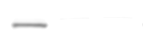

Supplement: Figure 4—figure supplement 1—source data 2. [file elife-106587-fig4-figsupp1-data2.zip › Figure 4-figure supplement 1 - source data 2/Figure 4-figure supplement 1C - source data 2/Figure 4-figure supplement 1C - ETFDH.tif]

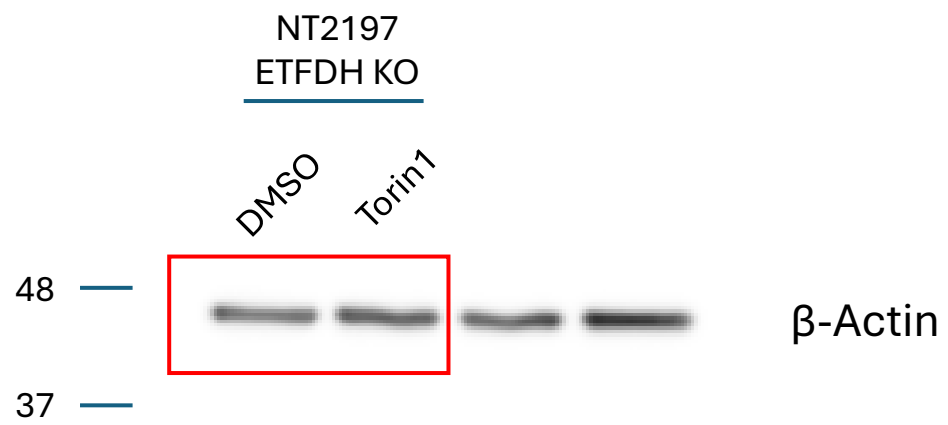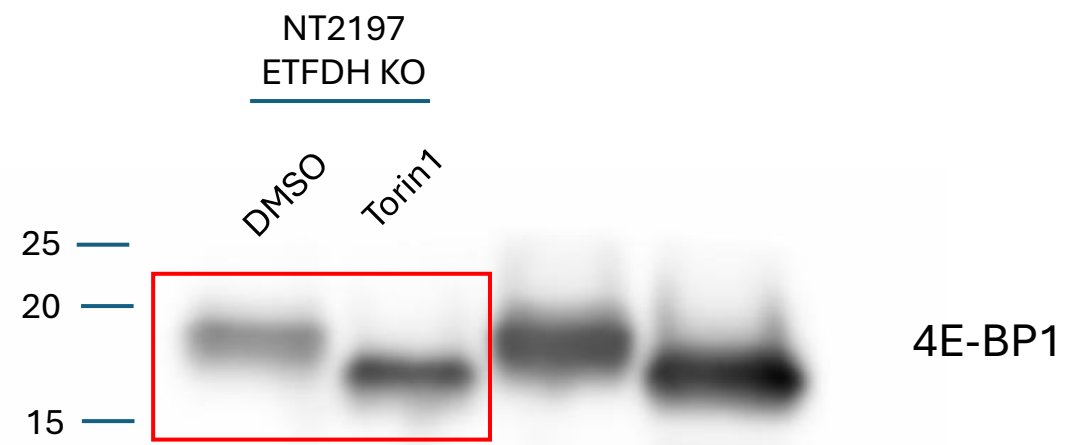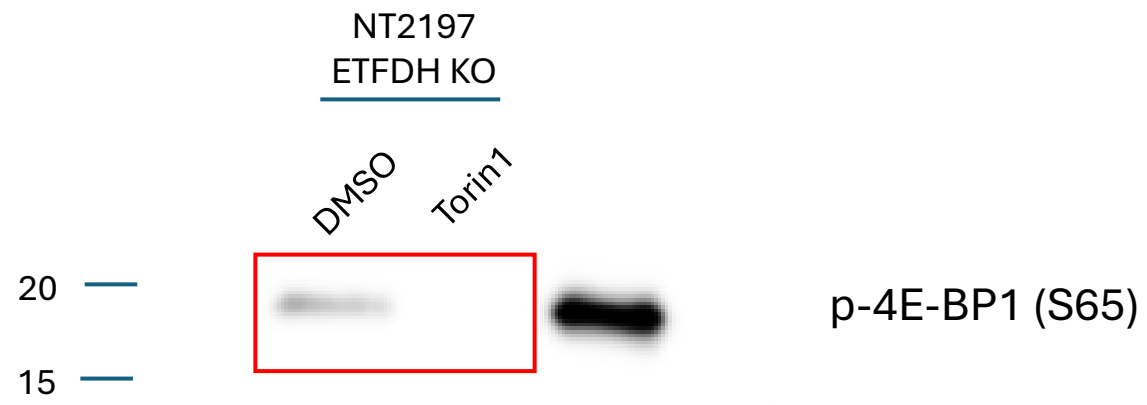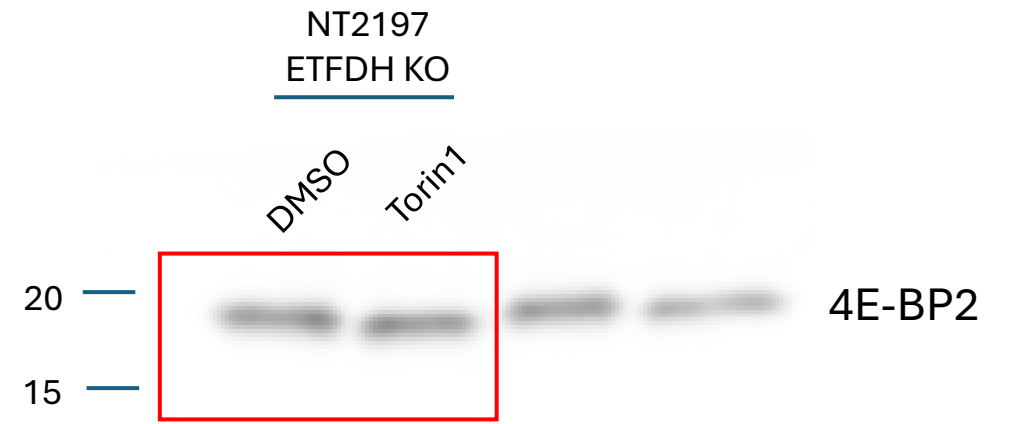

NT2197  
ETFDH KO

DMSO      Torin1

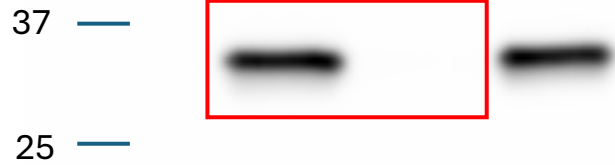

p-S6 (S240/244)

NT2197  
ETFDH KO

DMSO      Torin1

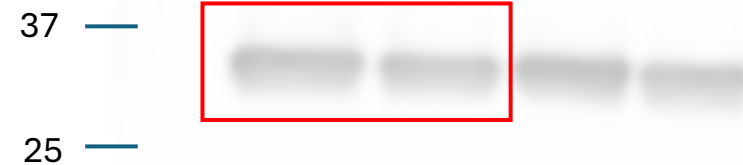

S6

NT2197  
ETFDH KO

DMSO      Torin1

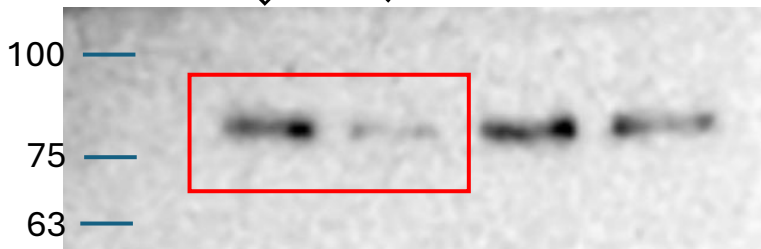

BCL-6

Supplement: Figure 5—source data 1. [file elife-106587-fig5-data1.zip › Figure 5 - source data 1/Figure 5B - source data 1/Figure 5B - source data 1.pdf]

HCT-116

WT      ETFDH KO

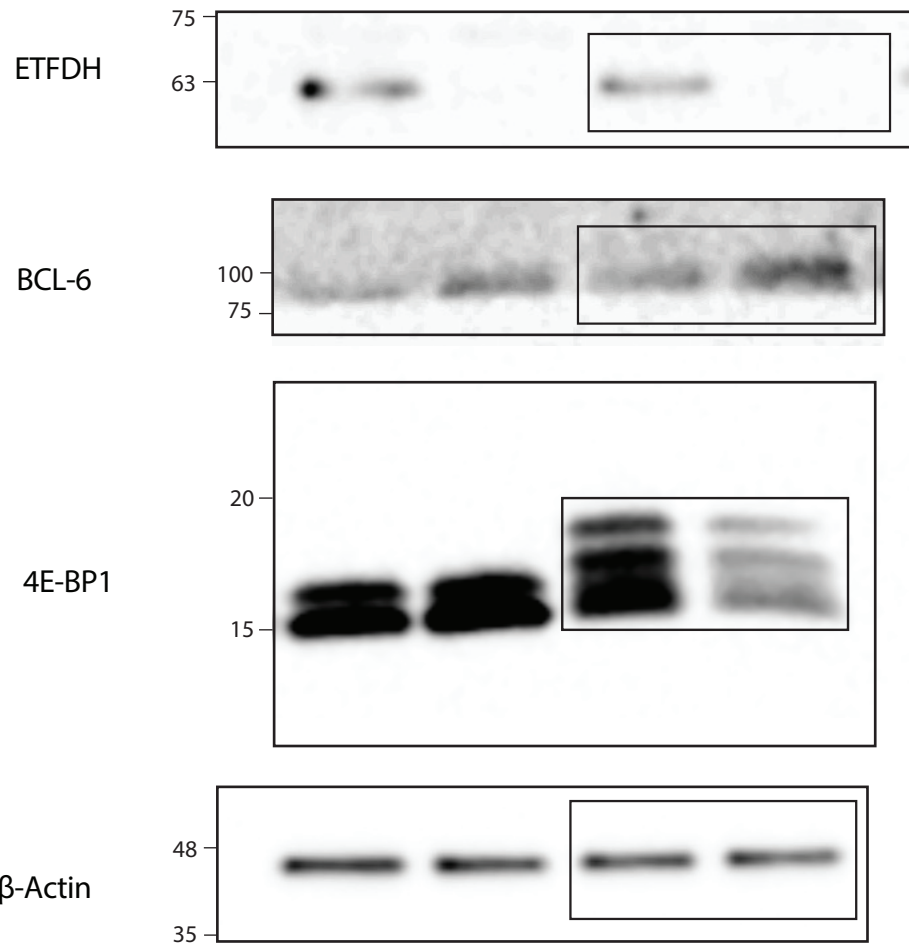

NT2197

WT      ETFDH KO

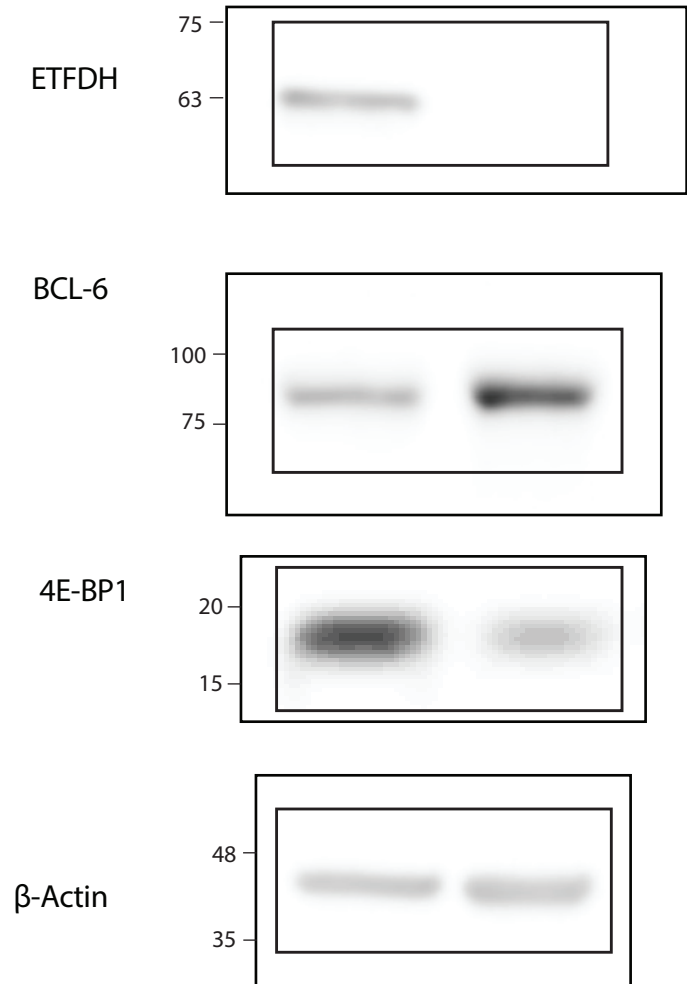

Supplement: Figure 5—source data 1. [file elife-106587-fig5-data1.zip › Figure 5 - source data 1/Figure 5A - source data 1/Figure 5A - source data 1.pdf]

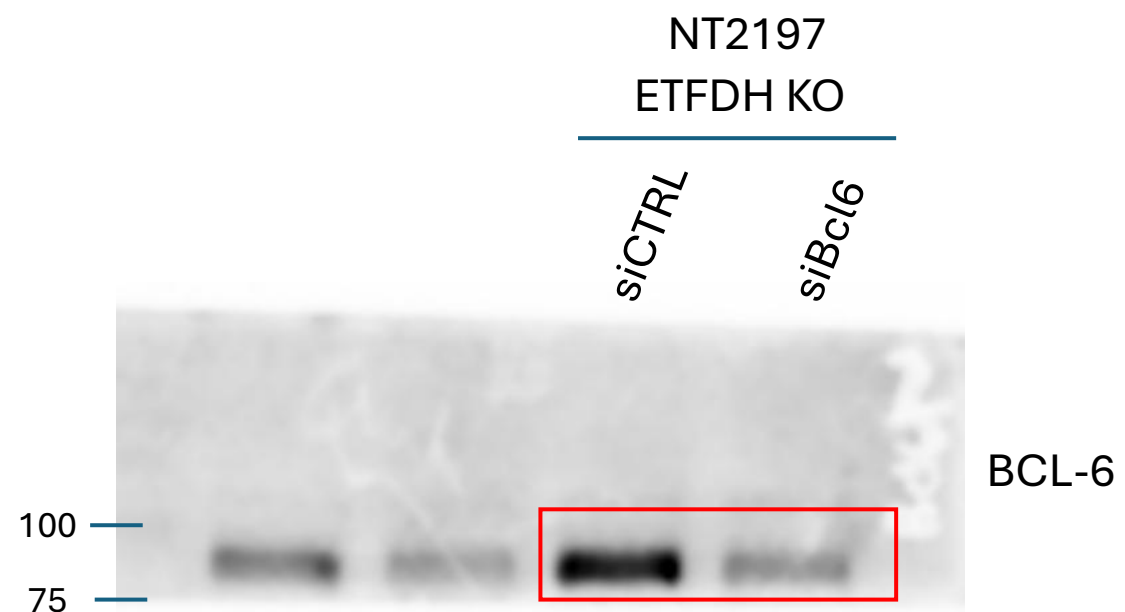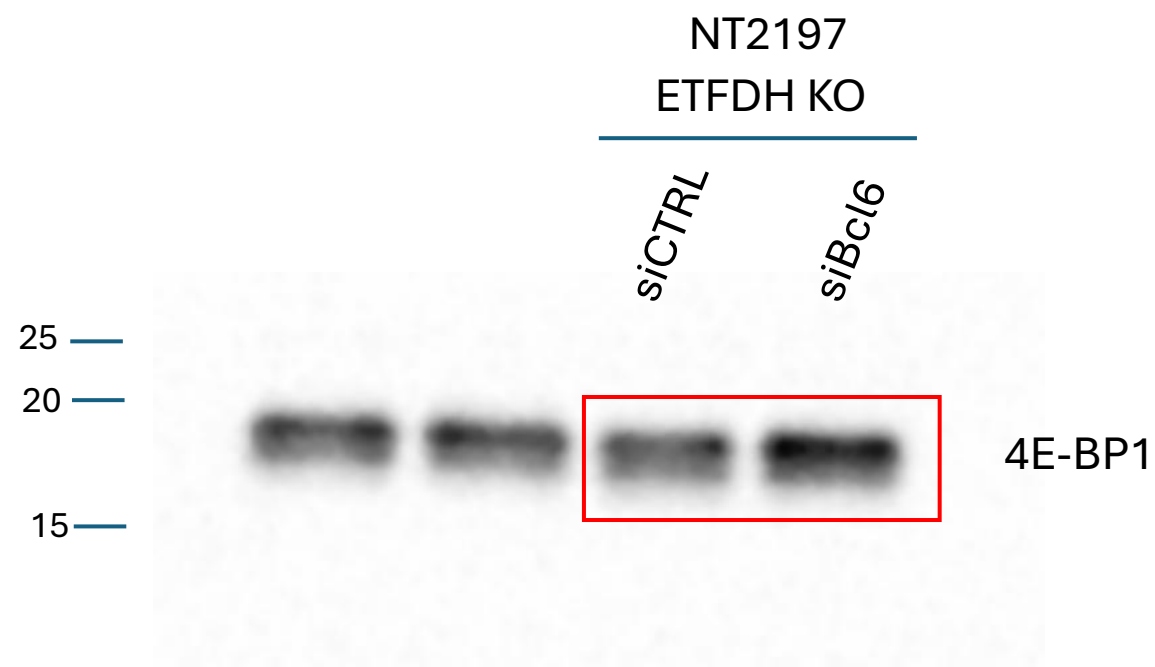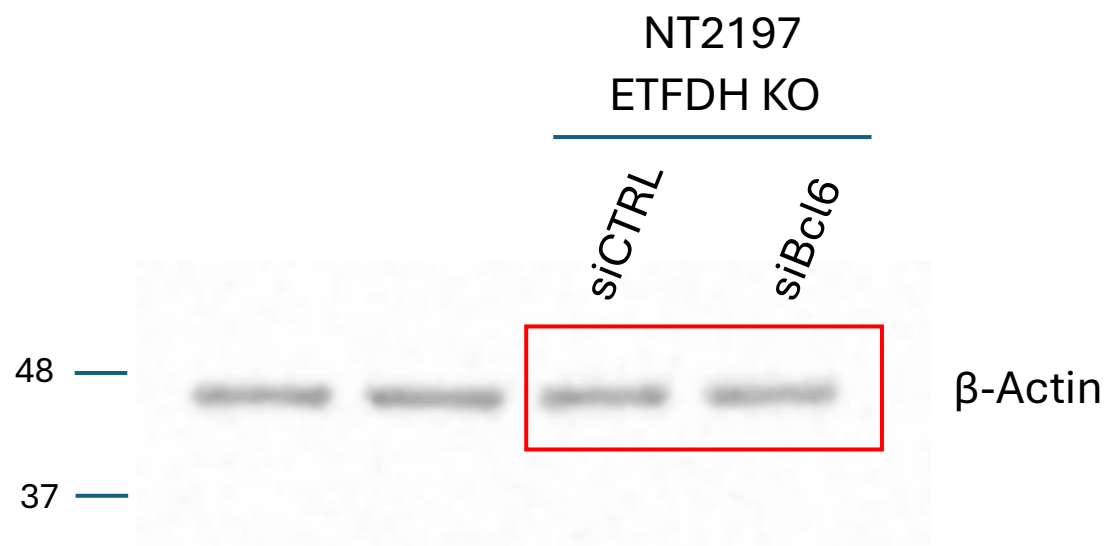

Supplement: Figure 5—source data 1. [file elife-106587-fig5-data1.zip › Figure 5 - source data 1/Figure 5G - source data 1/Figure 5G - source data 1.pdf]

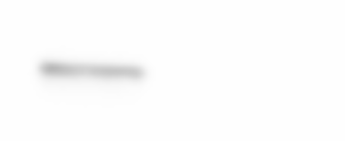

Supplement: Figure 5—source data 2. [file elife-106587-fig5-data2.zip › Figure 5 - source data 2/Figure 5A - source data 2/Figure 5A - NT2197 ETFDH.tif]

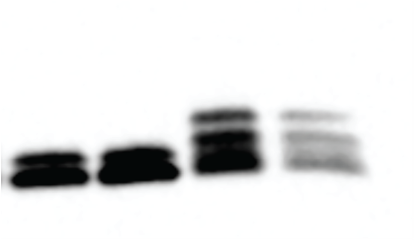

Supplement: Figure 5—source data 2. [file elife-106587-fig5-data2.zip › Figure 5 - source data 2/Figure 5A - source data 2/Figure 5A - HCT-116 4E-BP1.tif]

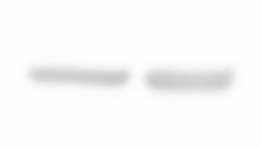

Supplement: Figure 5—source data 2. [file elife-106587-fig5-data2.zip › Figure 5 - source data 2/Figure 5A - source data 2/Figure 5A - NT2197 B_Actin.tif]

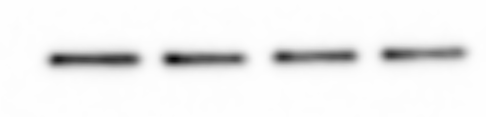

Supplement: Figure 5—source data 2. [file elife-106587-fig5-data2.zip › Figure 5 - source data 2/Figure 5A - source data 2/Figure 5A - HCT-116 B-Actin.tif]

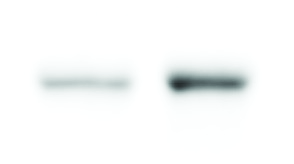

Supplement: Figure 5—source data 2. [file elife-106587-fig5-data2.zip › Figure 5 - source data 2/Figure 5A - source data 2/Figure 5A - NT2197 BCL-6.tiff]

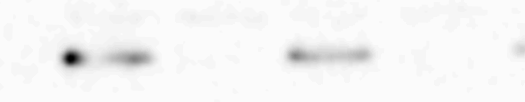

Supplement: Figure 5—source data 2. [file elife-106587-fig5-data2.zip › Figure 5 - source data 2/Figure 5A - source data 2/Figure 5A - HCT-116 ETFDH.tif]

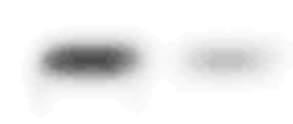

Supplement: Figure 5—source data 2. [file elife-106587-fig5-data2.zip › Figure 5 - source data 2/Figure 5A - source data 2/Figure 5A - NT2197 4E-BP1.tif]

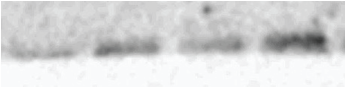

Supplement: Figure 5—source data 2. [file elife-106587-fig5-data2.zip › Figure 5 - source data 2/Figure 5A - source data 2/Figure 5A - HCT-116 BCL-6.tif]

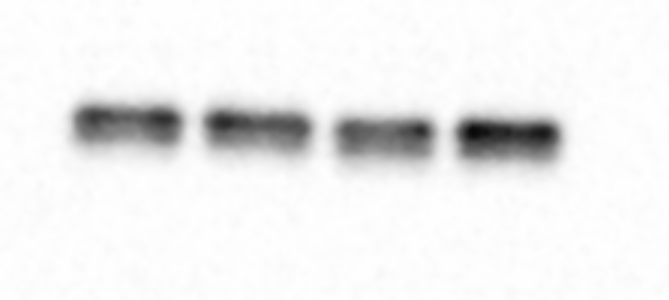

Supplement: Figure 5—source data 2. [file elife-106587-fig5-data2.zip › Figure 5 - source data 2/Figure 5G - source data 2/Figure 5G - 4E-BP1.tif]

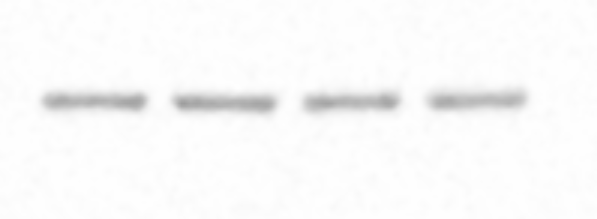

Supplement: Figure 5—source data 2. [file elife-106587-fig5-data2.zip › Figure 5 - source data 2/Figure 5G - source data 2/Figure 5G - B_Actin.tif]

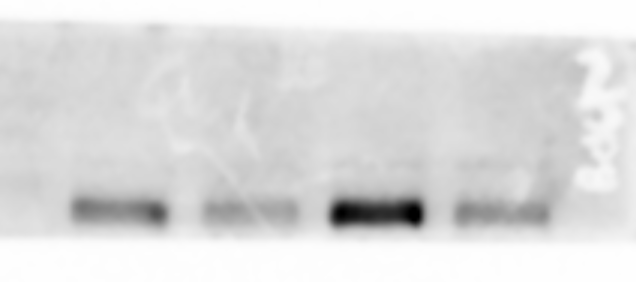

Supplement: Figure 5—source data 2. [file elife-106587-fig5-data2.zip › Figure 5 - source data 2/Figure 5G - source data 2/Figure 5G - BCL-6.tif]

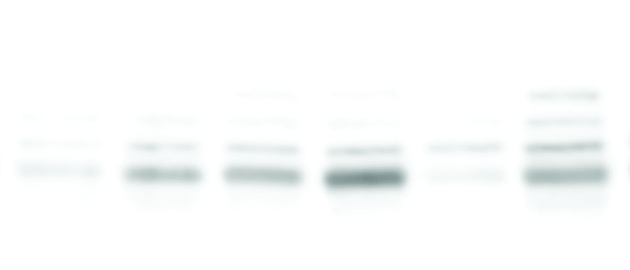

Supplement: Figure 5—source data 2. [file elife-106587-fig5-data2.zip › Figure 5 - source data 2/Figure 5H - source data 2/Figure 5H - BCL-6.tiff]

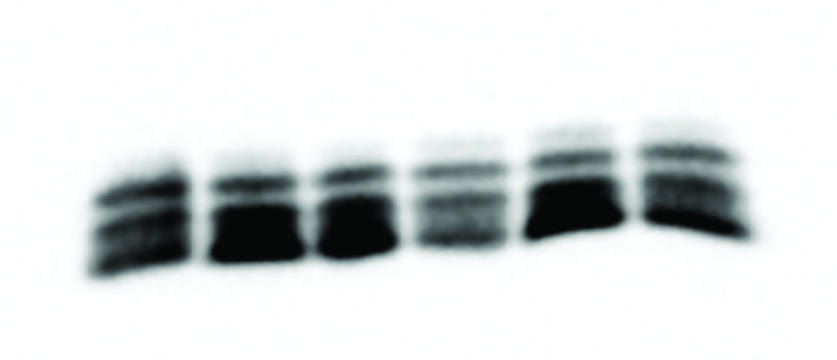

Supplement: Figure 5—source data 2. [file elife-106587-fig5-data2.zip › Figure 5 - source data 2/Figure 5H - source data 2/Figure 5H - 4E-BP1.tiff]

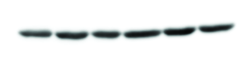

Supplement: Figure 5—source data 2. [file elife-106587-fig5-data2.zip › Figure 5 - source data 2/Figure 5H - source data 2/Figure 5H - B_Actin.tiff]

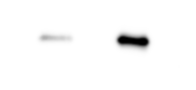

Supplement: Figure 5—source data 2. [file elife-106587-fig5-data2.zip › Figure 5 - source data 2/Figure 5B - source data 2/Figure 5B - p4E-BP1.tif]

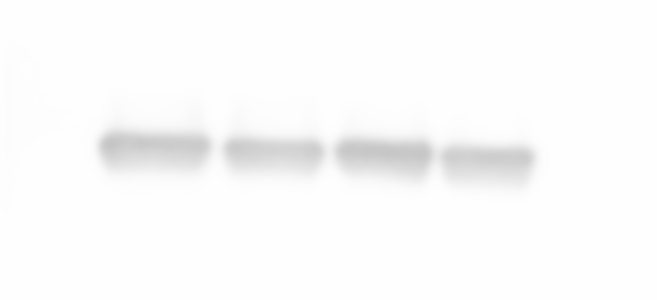

Supplement: Figure 5—source data 2. [file elife-106587-fig5-data2.zip › Figure 5 - source data 2/Figure 5B - source data 2/Figure 5B - S6.tif]

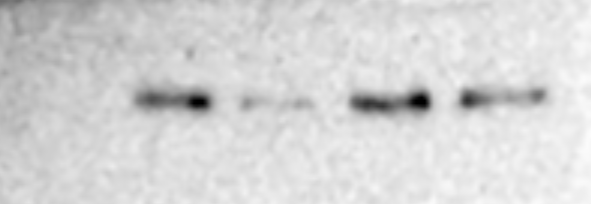

Supplement: Figure 5—source data 2. [file elife-106587-fig5-data2.zip › Figure 5 - source data 2/Figure 5B - source data 2/Figure 5B - BCL-6.tif]

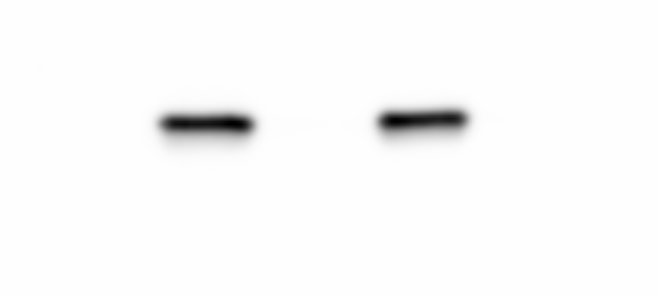

Supplement: Figure 5—source data 2. [file elife-106587-fig5-data2.zip › Figure 5 - source data 2/Figure 5B - source data 2/Figure 5B - pS6.tif]

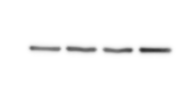

Supplement: Figure 5—source data 2. [file elife-106587-fig5-data2.zip › Figure 5 - source data 2/Figure 5B - source data 2/Figure 5B - B_Actin.tif]

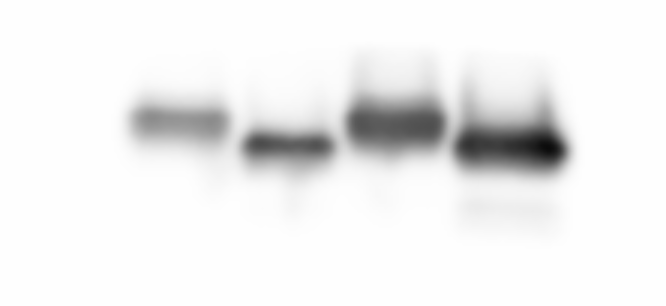

Supplement: Figure 5—source data 2. [file elife-106587-fig5-data2.zip › Figure 5 - source data 2/Figure 5B - source data 2/Figure 5B - 4E-BP1.tif]

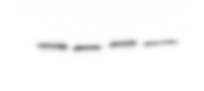

Supplement: Figure 5—source data 2. [file elife-106587-fig5-data2.zip › Figure 5 - source data 2/Figure 5B - source data 2/Figure 5B - 4E-BP2.tif]

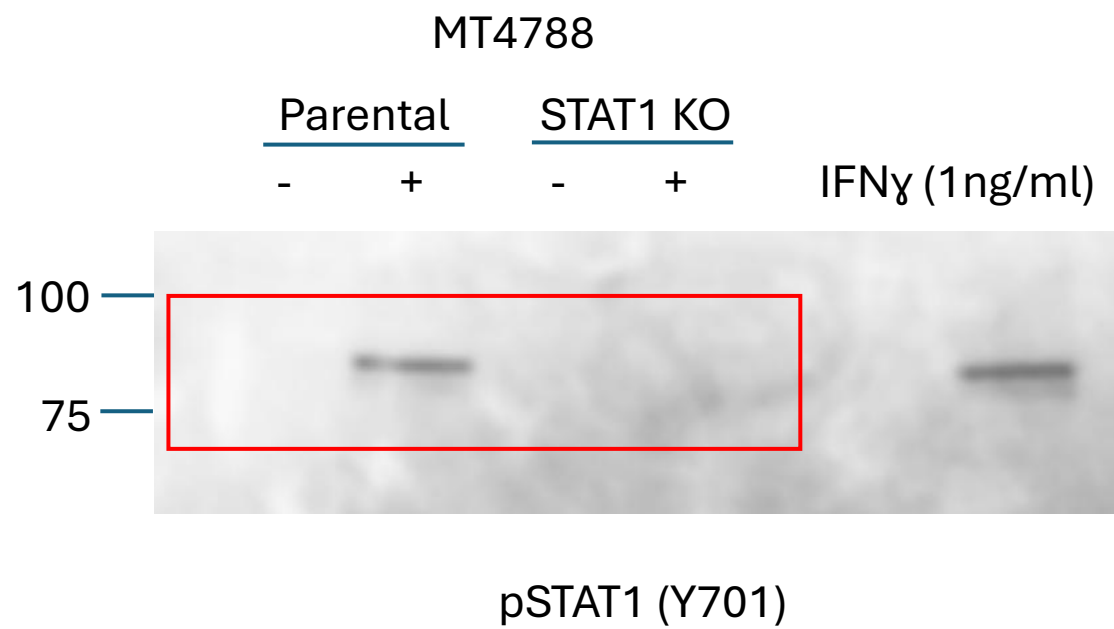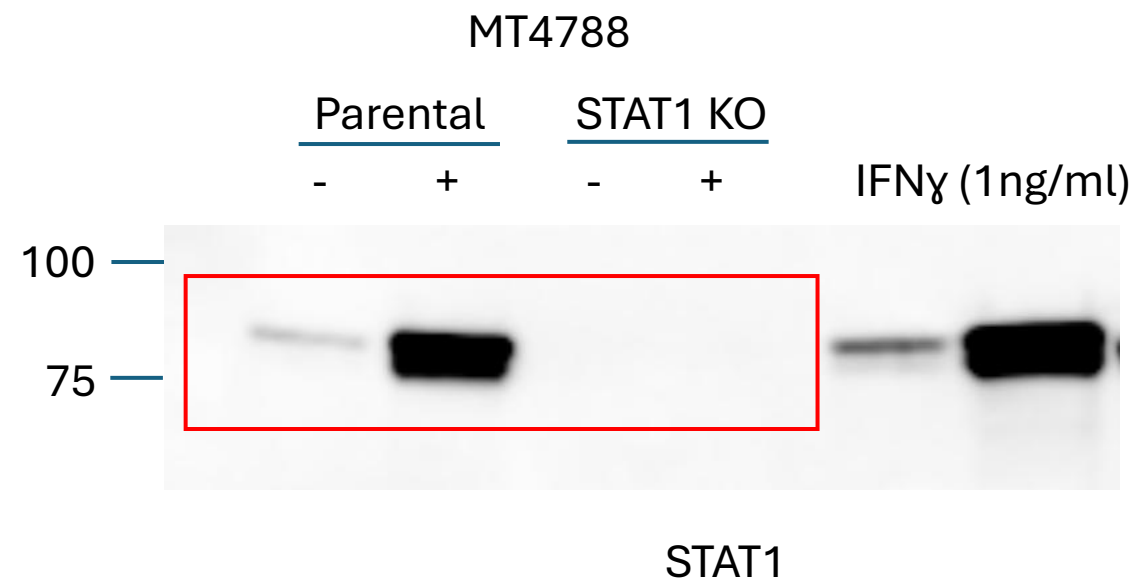

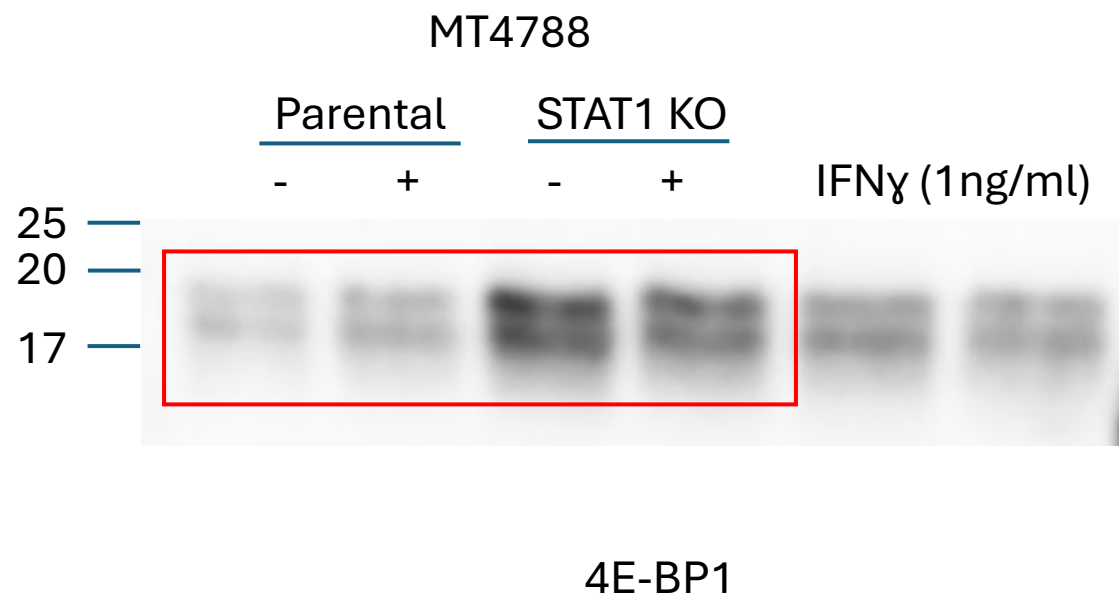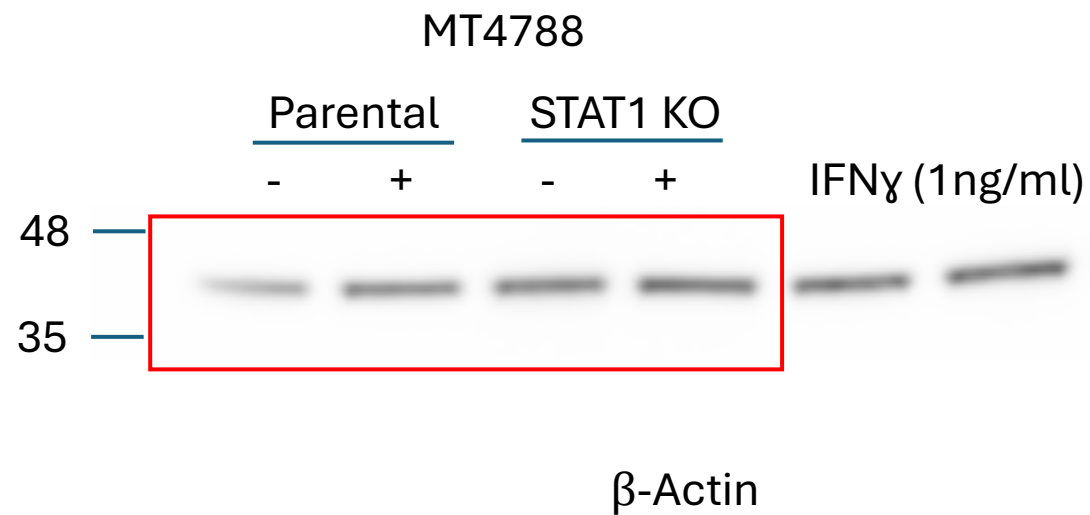

Supplement: Figure 5—figure supplement 1—source data 1. [file elife-106587-fig5-figsupp1-data1.zip › Figure 5-figure supplement 1 - source data 1/Figure 5-figure supplement 1B - source data 1/Figure 4-figure supplement 1B - source data 1.pdf]

HCT-116

WT    ETFDH KO

ETFDH

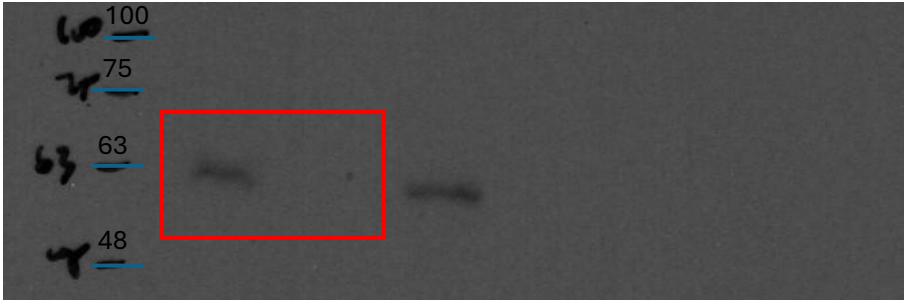

HCT-116

WT    ETFDH KO

STAT1

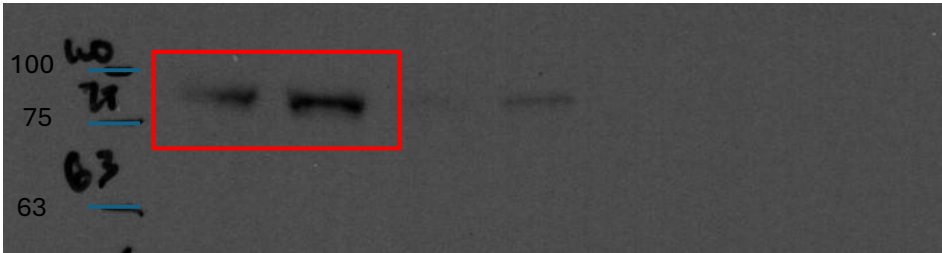

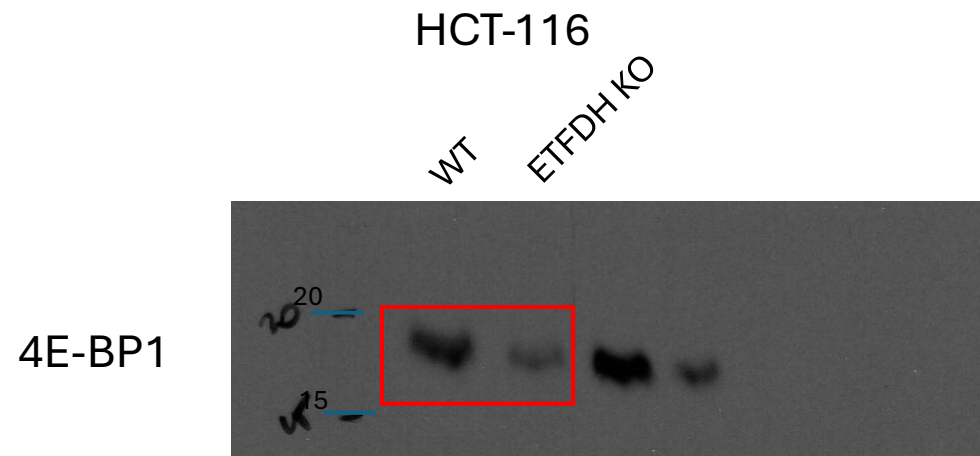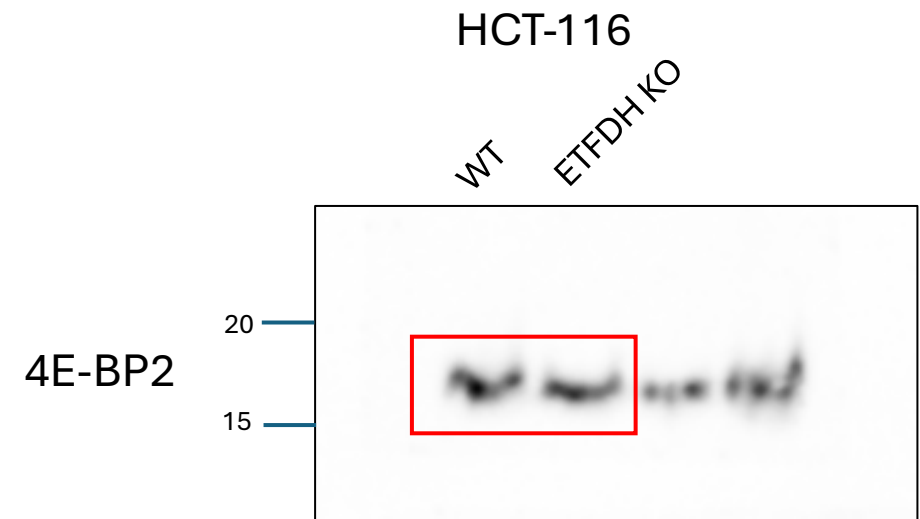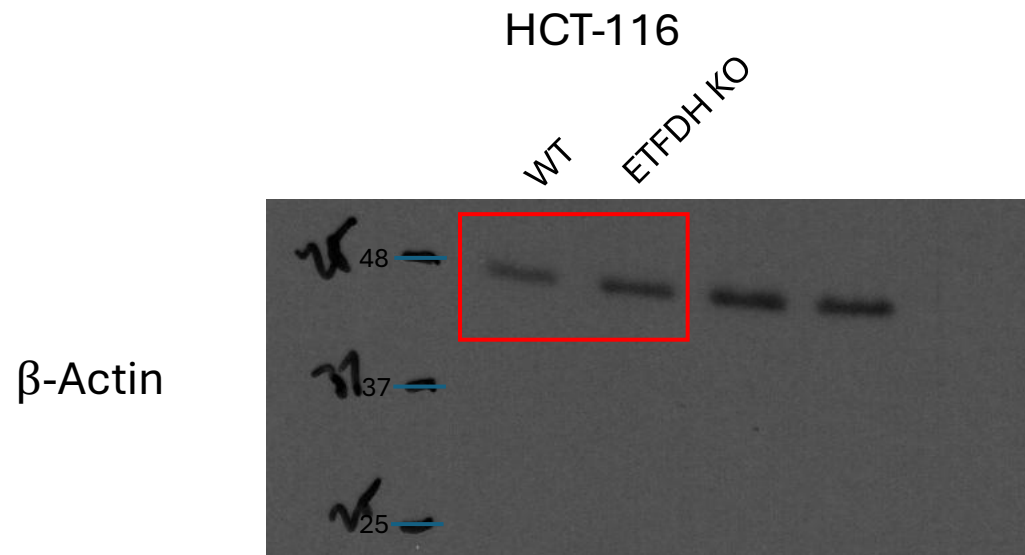

Supplement: Figure 5—figure supplement 1—source data 1. [file elife-106587-fig5-figsupp1-data1.zip › Figure 5-figure supplement 1 - source data 1/Figure 5-figure supplement 1C - source data 1/Figure 5-figure supplement 1C - source data 1.pdf]

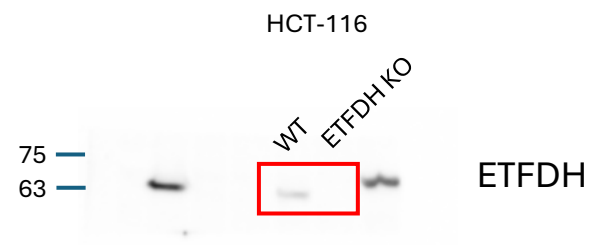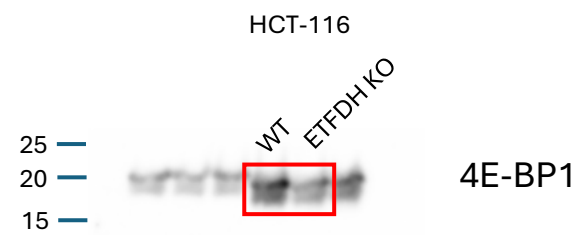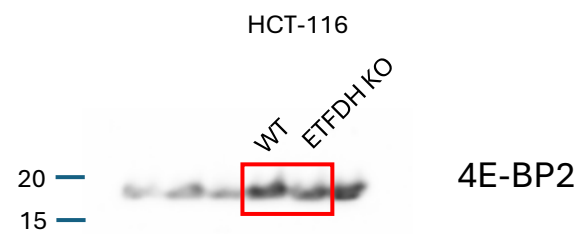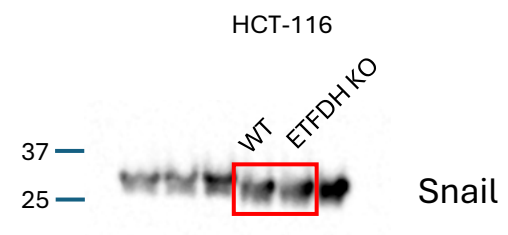

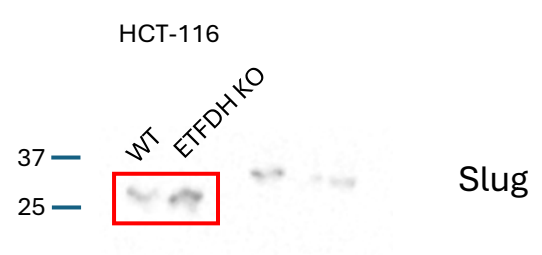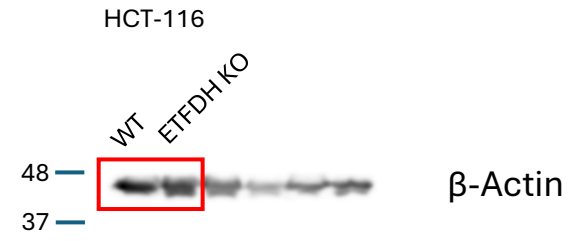

Supplement: Figure 5—figure supplement 1—source data 1. [file elife-106587-fig5-figsupp1-data1.zip › Figure 5-figure supplement 1 - source data 1/Figure 5-figure supplement 1A - source data 1/Figure 5-figure supplement 1A - source data 1.pdf]

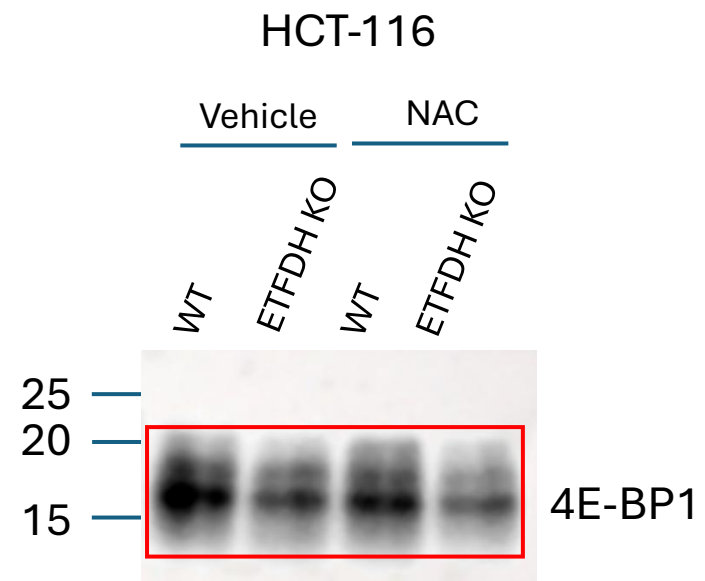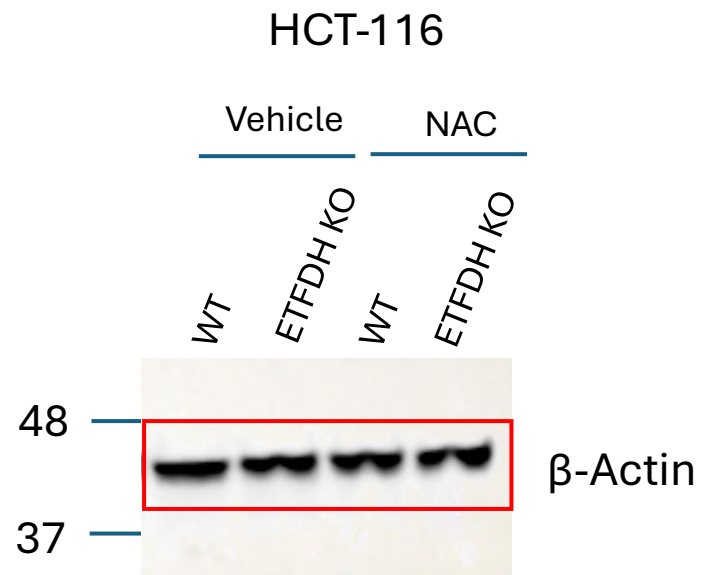

Supplement: Figure 5—figure supplement 1—source data 1. [file elife-106587-fig5-figsupp1-data1.zip › Figure 5-figure supplement 1 - source data 1/Figure 5-figure supplement 1G - source data 1/Figure 5-figure supplement 1G - source data 1.pdf]

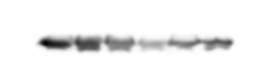

Supplement: Figure 5—figure supplement 1—source data 2. [file elife-106587-fig5-figsupp1-data2.zip › Figure 5-figure supplement 1 - source data 2/Figure 5-figure supplement 1A - source data 2/Figure 5-figure supplement 1A - B_Actin.tif]

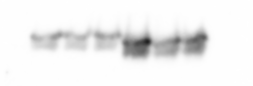

Supplement: Figure 5—figure supplement 1—source data 2. [file elife-106587-fig5-figsupp1-data2.zip › Figure 5-figure supplement 1 - source data 2/Figure 5-figure supplement 1A - source data 2/Figure 5-figure supplement 1A - 4E-BP1.tif]

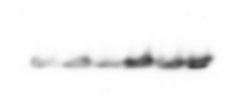

Supplement: Figure 5—figure supplement 1—source data 2. [file elife-106587-fig5-figsupp1-data2.zip › Figure 5-figure supplement 1 - source data 2/Figure 5-figure supplement 1A - source data 2/Figure 5-figure supplement 1A - 4E-BP2.tif]

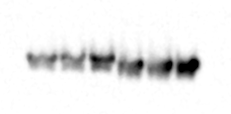

Supplement: Figure 5—figure supplement 1—source data 2. [file elife-106587-fig5-figsupp1-data2.zip › Figure 5-figure supplement 1 - source data 2/Figure 5-figure supplement 1A - source data 2/Figure 5-figure supplement 1 - Snail.tif]

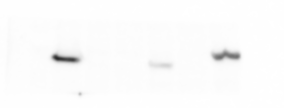

Supplement: Figure 5—figure supplement 1—source data 2. [file elife-106587-fig5-figsupp1-data2.zip › Figure 5-figure supplement 1 - source data 2/Figure 5-figure supplement 1A - source data 2/Figure 5-figure supplement 1A - ETFDH.tif]

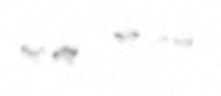

Supplement: Figure 5—figure supplement 1—source data 2. [file elife-106587-fig5-figsupp1-data2.zip › Figure 5-figure supplement 1 - source data 2/Figure 5-figure supplement 1A - source data 2/Figure 5-figure supplement 1A - Slug.tif]

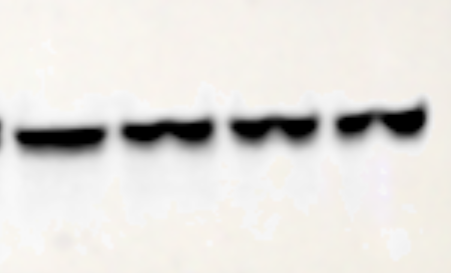

Supplement: Figure 5—figure supplement 1—source data 2. [file elife-106587-fig5-figsupp1-data2.zip › Figure 5-figure supplement 1 - source data 2/Figure 5-figure supplement 1G - source data 2/Figure 5-figure supplement 1G - B_Actin.tiff]

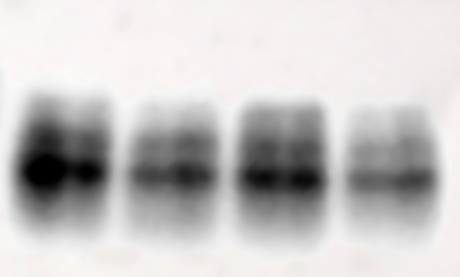

Supplement: Figure 5—figure supplement 1—source data 2. [file elife-106587-fig5-figsupp1-data2.zip › Figure 5-figure supplement 1 - source data 2/Figure 5-figure supplement 1G - source data 2/Figure 5-figure supplement 1G - 4E-BP1.tiff]

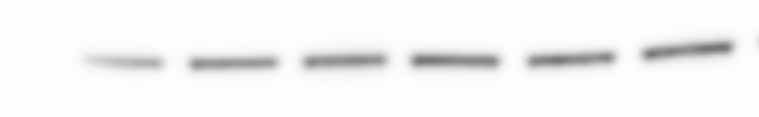

Supplement: Figure 5—figure supplement 1—source data 2. [file elife-106587-fig5-figsupp1-data2.zip › Figure 5-figure supplement 1 - source data 2/Figure 5-figure supplement 1B - source data 2/Figure 5-figure supplement 1B - B_Actin.tif]

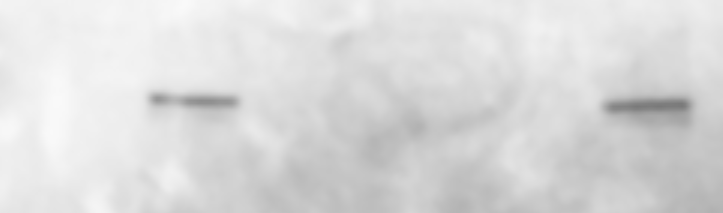

Supplement: Figure 5—figure supplement 1—source data 2. [file elife-106587-fig5-figsupp1-data2.zip › Figure 5-figure supplement 1 - source data 2/Figure 5-figure supplement 1B - source data 2/Figure 5-figure supplement 1B - pSTAT1.tif]

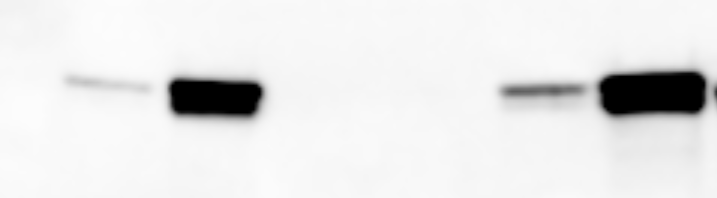

Supplement: Figure 5—figure supplement 1—source data 2. [file elife-106587-fig5-figsupp1-data2.zip › Figure 5-figure supplement 1 - source data 2/Figure 5-figure supplement 1B - source data 2/Figure 5-figure supplement 1B - STAT1.tif]

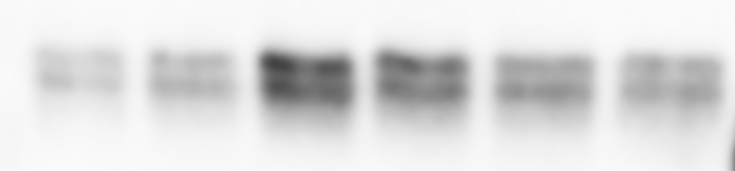

Supplement: Figure 5—figure supplement 1—source data 2. [file elife-106587-fig5-figsupp1-data2.zip › Figure 5-figure supplement 1 - source data 2/Figure 5-figure supplement 1B - source data 2/Figure 5-figure supplement 1B - 4E-BP1.tif]

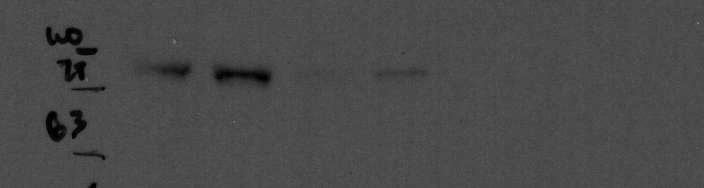

Supplement: Figure 5—figure supplement 1—source data 2. [file elife-106587-fig5-figsupp1-data2.zip › Figure 5-figure supplement 1 - source data 2/Figure 5-figure supplement 1C - source data 2/Figure 5-figure supplement 1C - STAT1.tif]

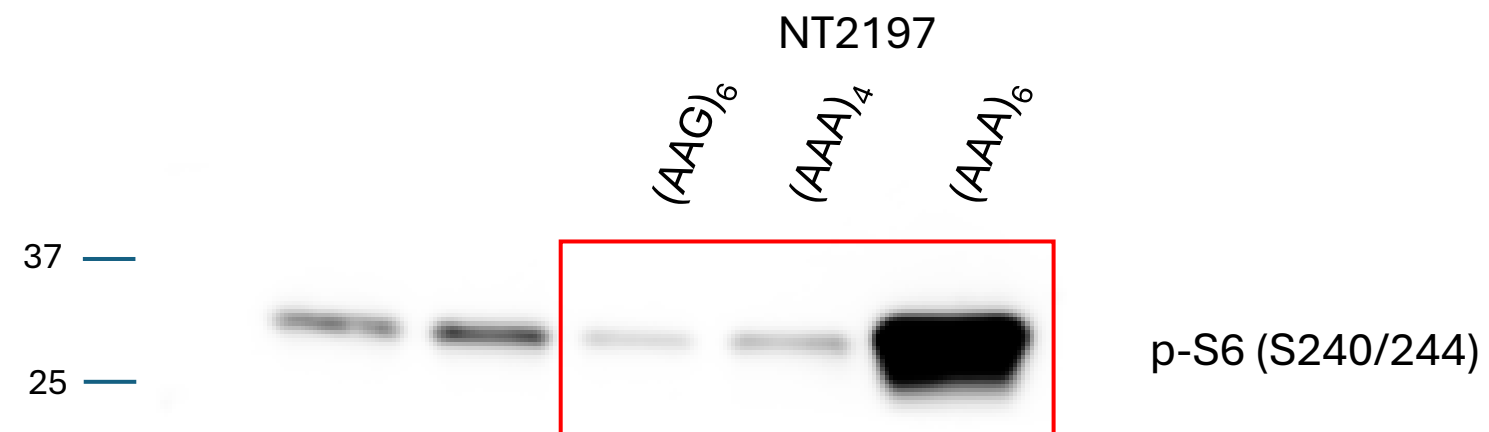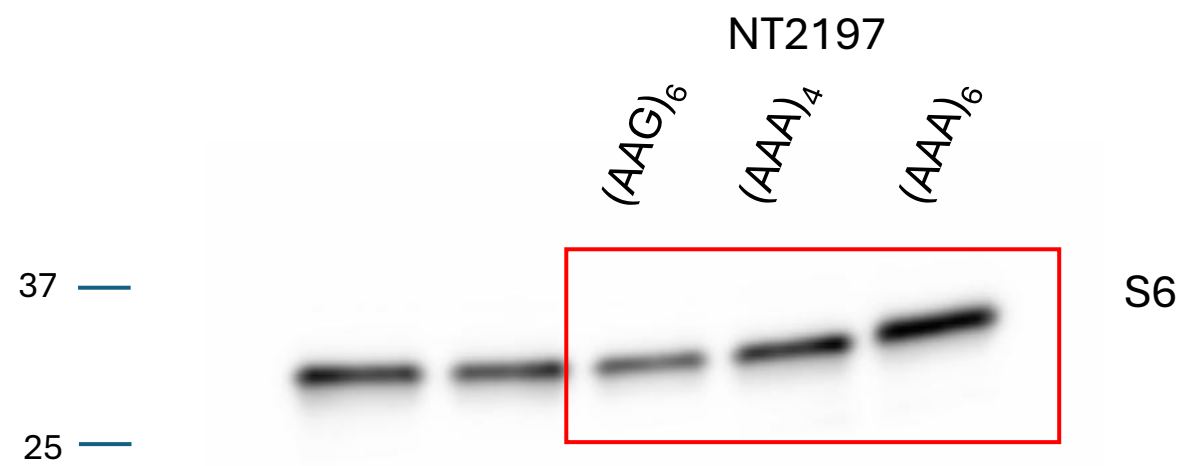

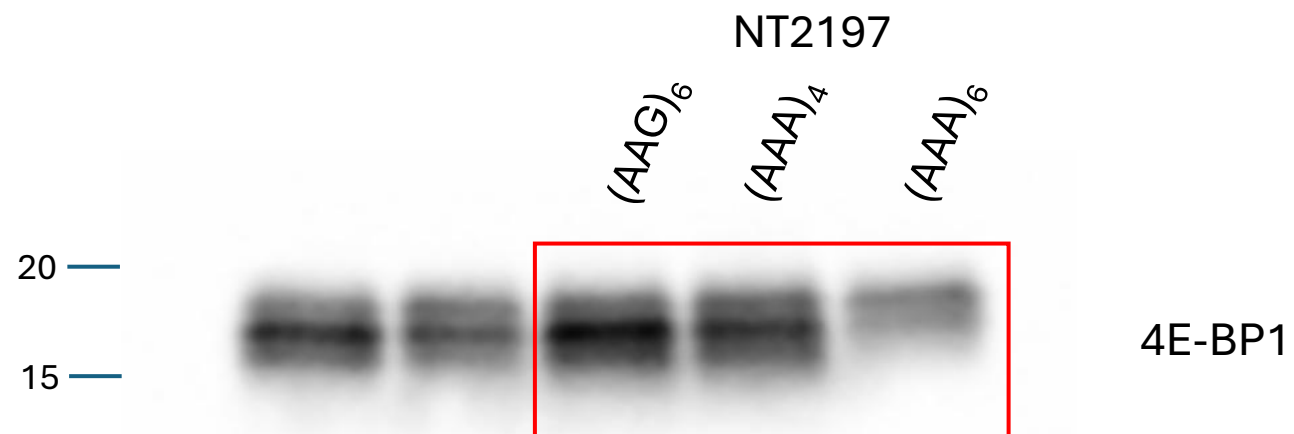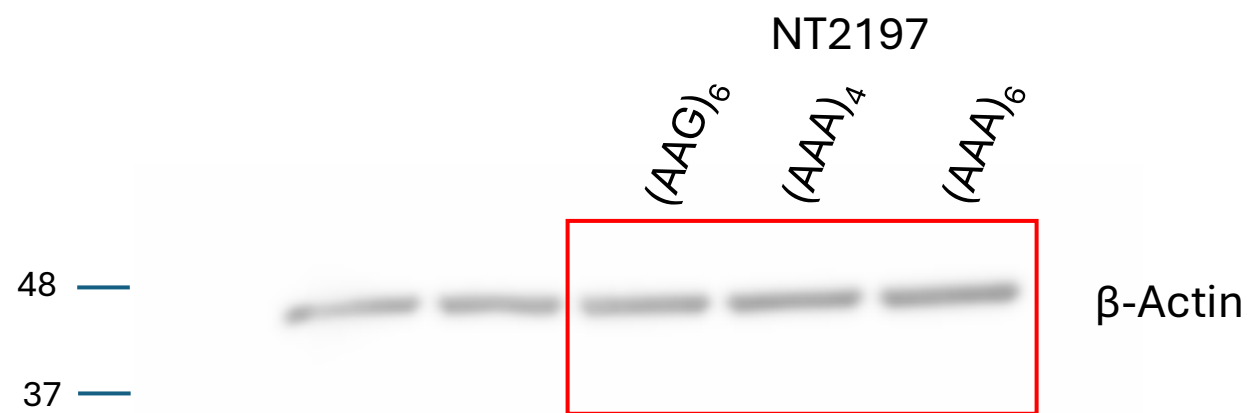

NT2197

$(AAG)_6$

$(AAA)_4$

$(AAA)_6$

20

15

4E-BP2

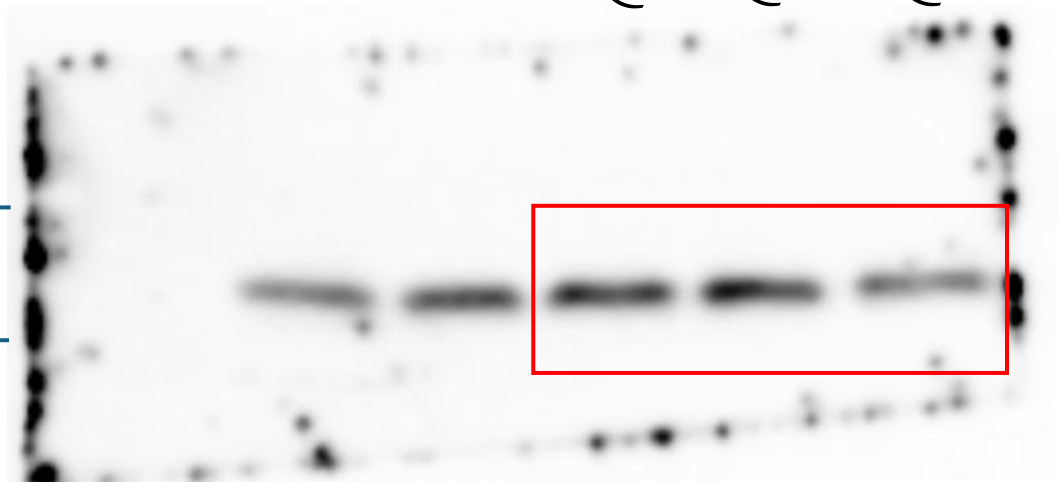

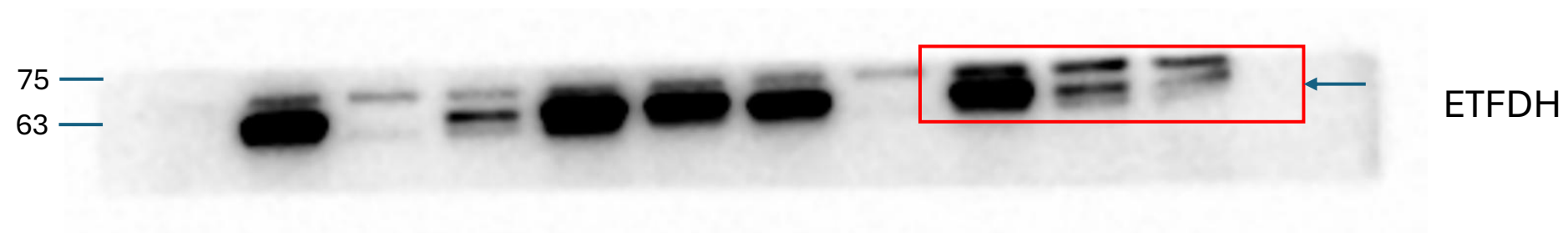

NT2197

(AAG)<sub>6</sub>

(AAA)<sub>4</sub>

(AAA)<sub>6</sub>

100 —  
75 —

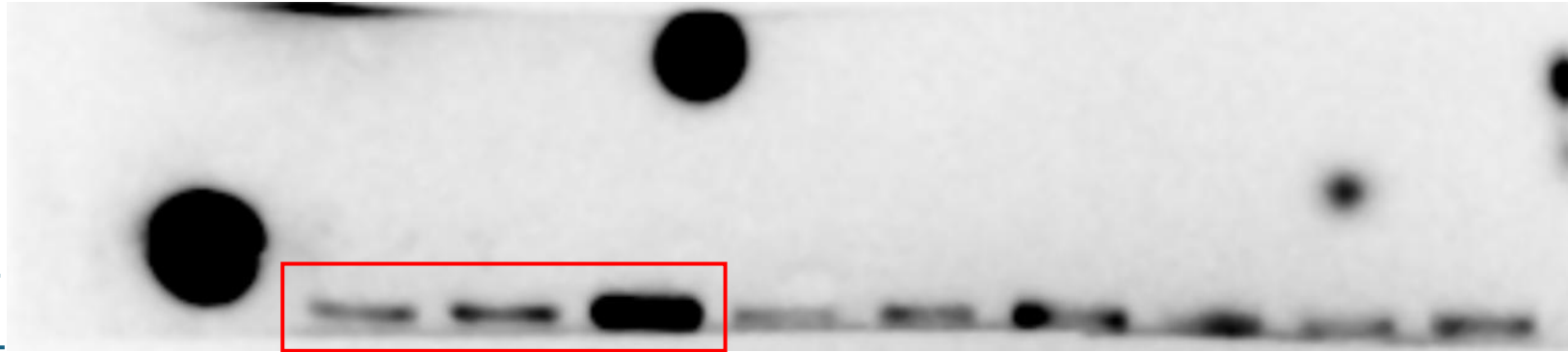

BCL-6

Supplement: Figure 6—source data 1. [file elife-106587-fig6-data1.zip › Figure 6 - source data 1/Figure 6C - source data 1/Figure 6C - source data 1.pdf]

## ETFDH

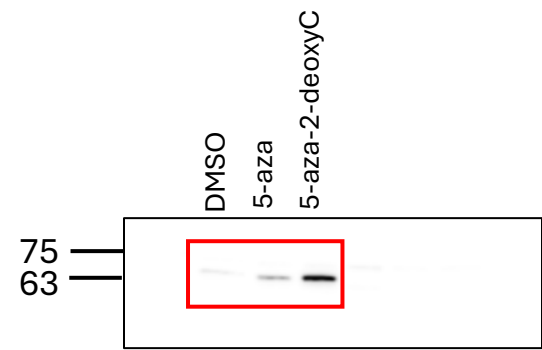

## $\beta$ -Actin

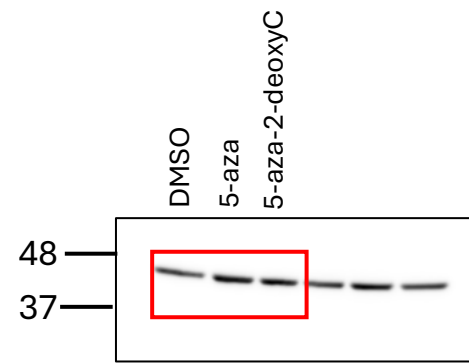

Supplement: Figure 6—source data 1. [file elife-106587-fig6-data1.zip › Figure 6 - source data 1/Figure 6B - source data 1/Figure 6B - source data 1.pdf]

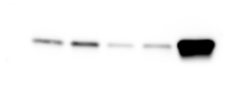

Supplement: Figure 6—source data 2. [file elife-106587-fig6-data2.zip › Figure 6 - source data 2/Figure 6C - source data 2/Figure 6C - pS6.tif]

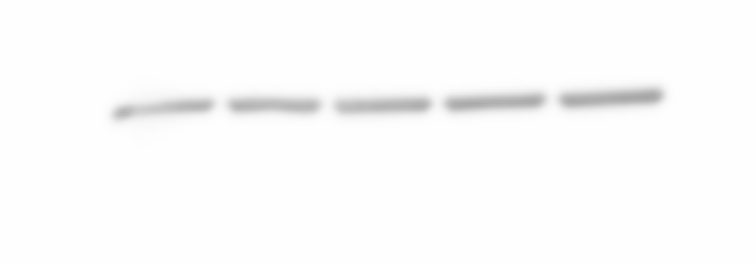

Supplement: Figure 6—source data 2. [file elife-106587-fig6-data2.zip › Figure 6 - source data 2/Figure 6C - source data 2/Figure 6C - B_Actin.tif]

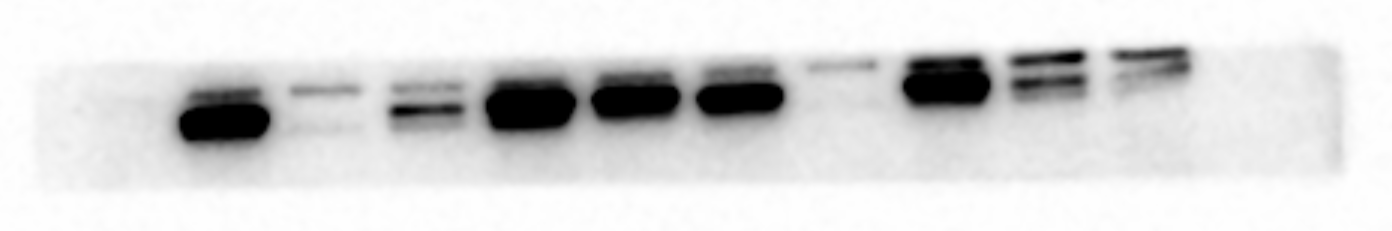

Supplement: Figure 6—source data 2. [file elife-106587-fig6-data2.zip › Figure 6 - source data 2/Figure 6C - source data 2/Figure 6C - ETFDH.tif]

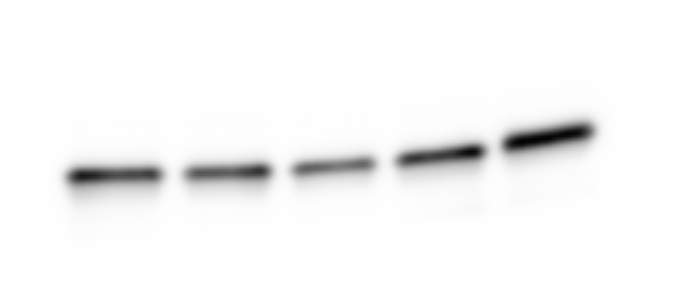

Supplement: Figure 6—source data 2. [file elife-106587-fig6-data2.zip › Figure 6 - source data 2/Figure 6C - source data 2/Figure 6C - S6.tif]

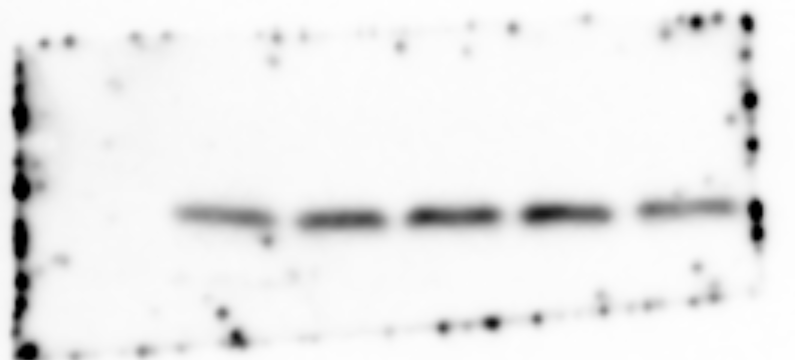

Supplement: Figure 6—source data 2. [file elife-106587-fig6-data2.zip › Figure 6 - source data 2/Figure 6C - source data 2/Figure 6C - 4E-BP2.tif]

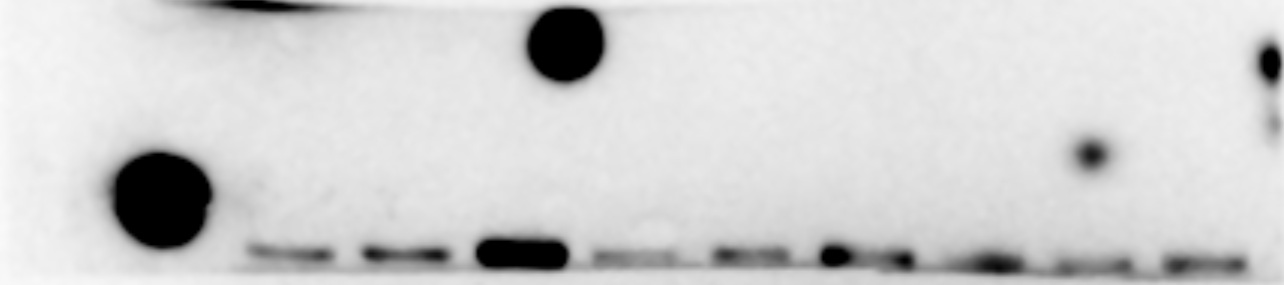

Supplement: Figure 6—source data 2. [file elife-106587-fig6-data2.zip › Figure 6 - source data 2/Figure 6C - source data 2/Figure 6C - BCL-6.tif]

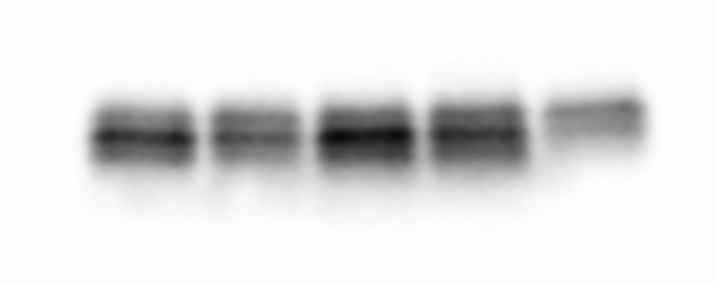

Supplement: Figure 6—source data 2. [file elife-106587-fig6-data2.zip › Figure 6 - source data 2/Figure 6C - source data 2/Figure 6C - 4E-BP1.tif]

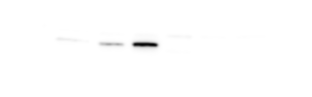

Supplement: Figure 6—source data 2. [file elife-106587-fig6-data2.zip › Figure 6 - source data 2/Figure 6B - source data 2/Figure 6B - ETFDH.tif]

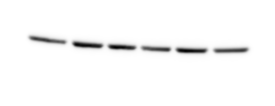

Supplement: Figure 6—source data 2. [file elife-106587-fig6-data2.zip › Figure 6 - source data 2/Figure 6B - source data 2/Figure 6B - B_Actin.tif]

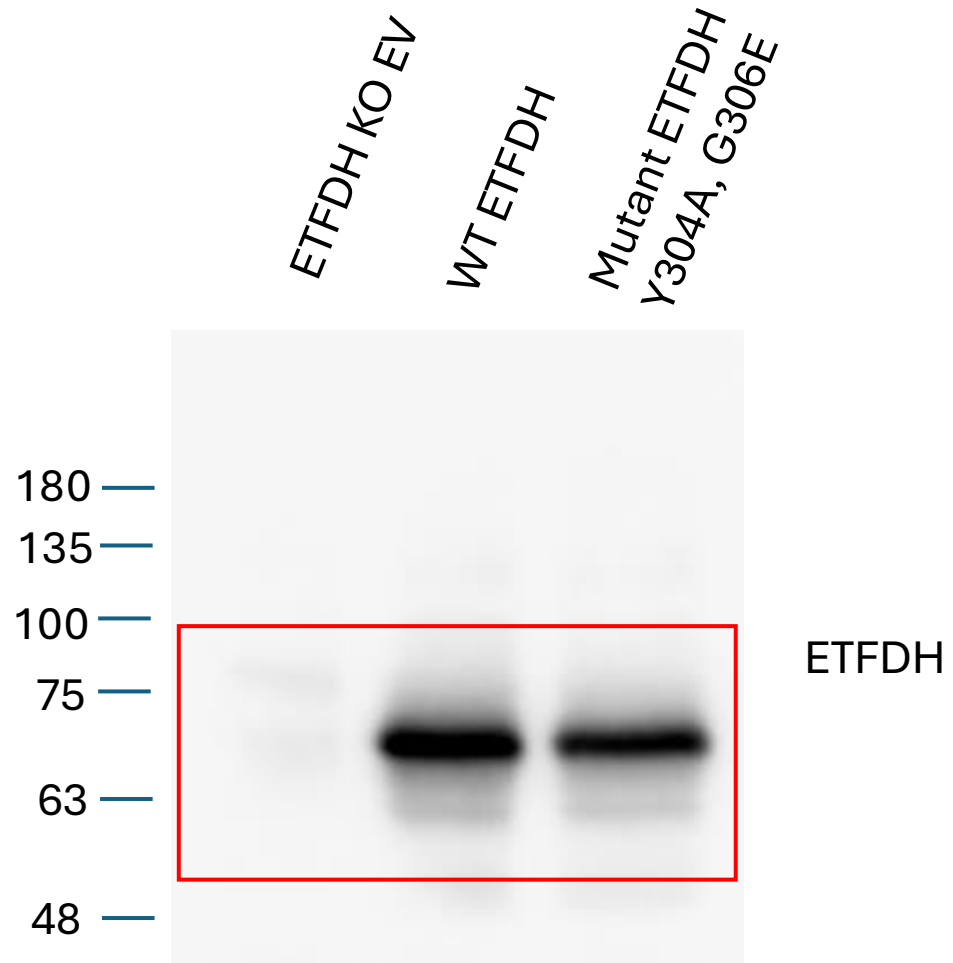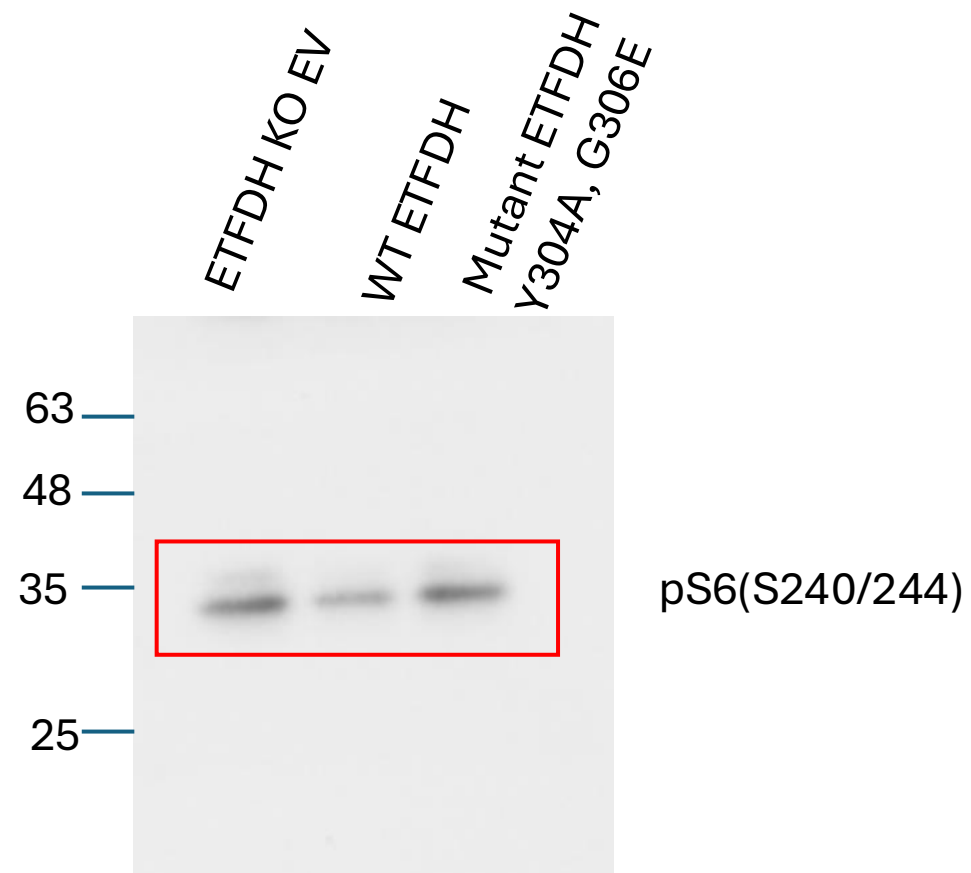

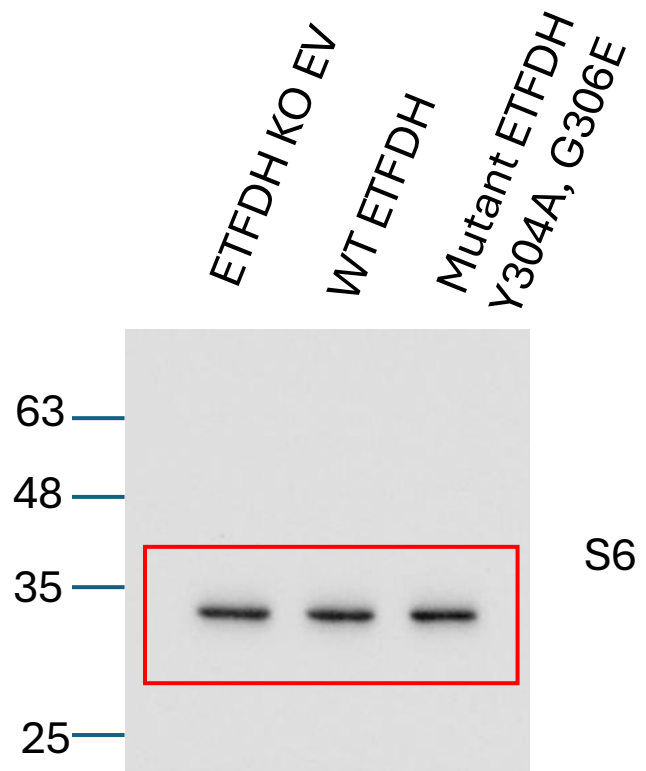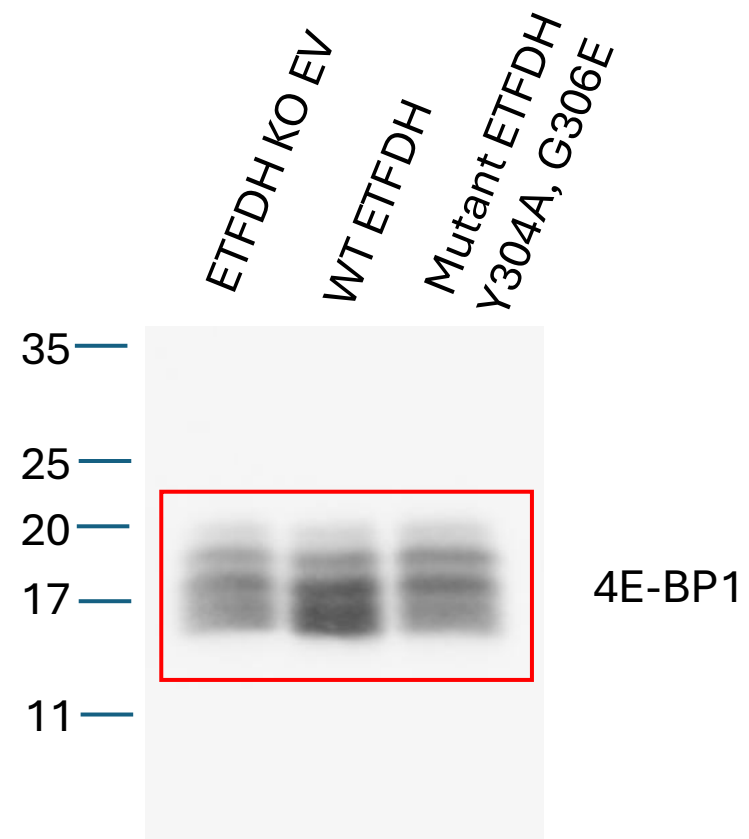

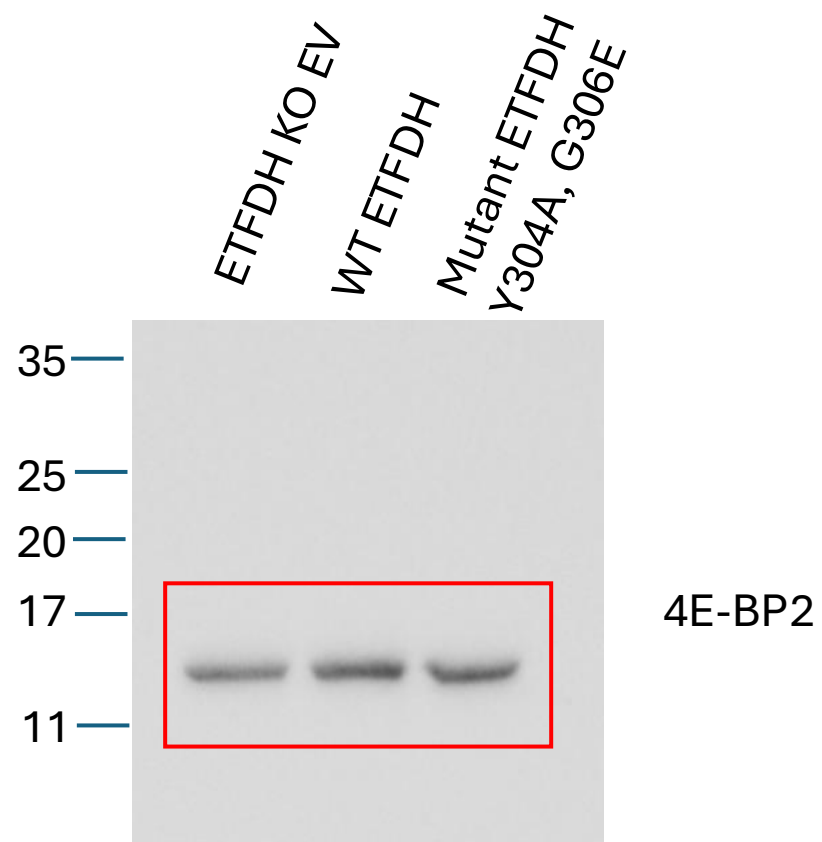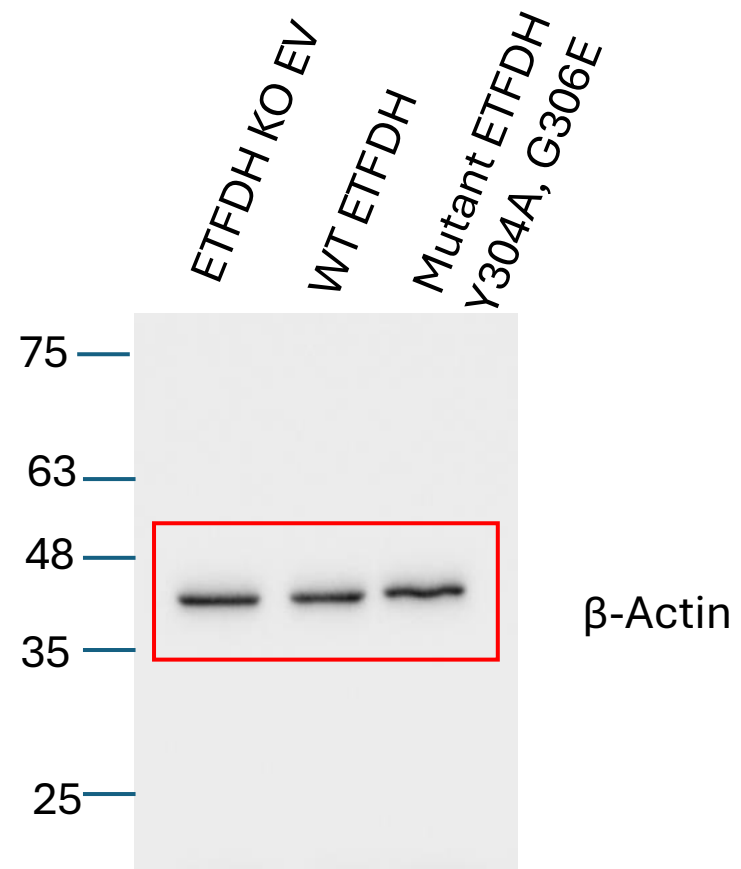

Supplement: Figure 6—figure supplement 1—source data 1. [file elife-106587-fig6-figsupp1-data1.zip › Figure 6-figure supplement 1 - source data 1/Figure 6-figure supplement 1C - source data 1/Figure6-figure supplement 1C - source data 1.pdf]

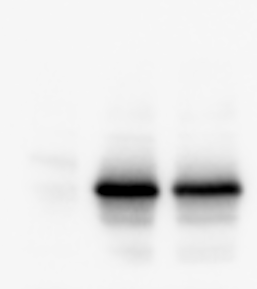

Supplement: Figure 6—figure supplement 1—source data 2. [file elife-106587-fig6-figsupp1-data2.zip › Figure 6-figure supplement 1 - source data 2/Figure 6-figure supplement 1C - source data 2/Figure 6-figure supplement 1C - ETFDH.tiff]

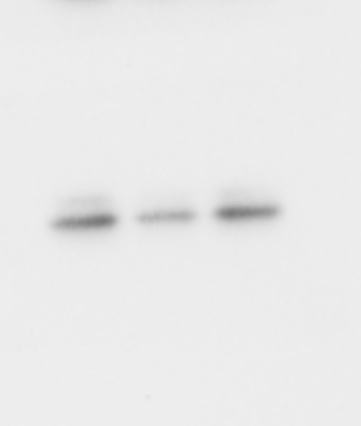

Supplement: Figure 6—figure supplement 1—source data 2. [file elife-106587-fig6-figsupp1-data2.zip › Figure 6-figure supplement 1 - source data 2/Figure 6-figure supplement 1C - source data 2/Figure 6-figure supplement 1C - pS6.tiff]

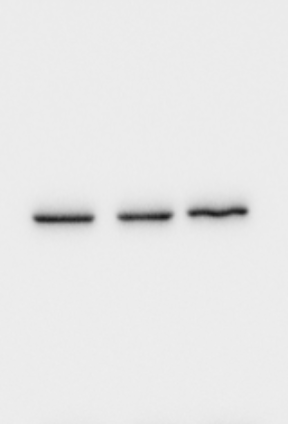

Supplement: Figure 6—figure supplement 1—source data 2. [file elife-106587-fig6-figsupp1-data2.zip › Figure 6-figure supplement 1 - source data 2/Figure 6-figure supplement 1C - source data 2/Figure 6-figure supplement 1C - B_Actin.tiff]

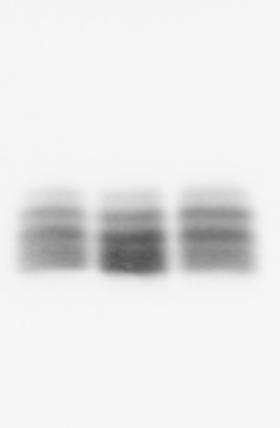

Supplement: Figure 6—figure supplement 1—source data 2. [file elife-106587-fig6-figsupp1-data2.zip › Figure 6-figure supplement 1 - source data 2/Figure 6-figure supplement 1C - source data 2/Figure 6-figure supplement 1C - 4E-BP1.tiff]

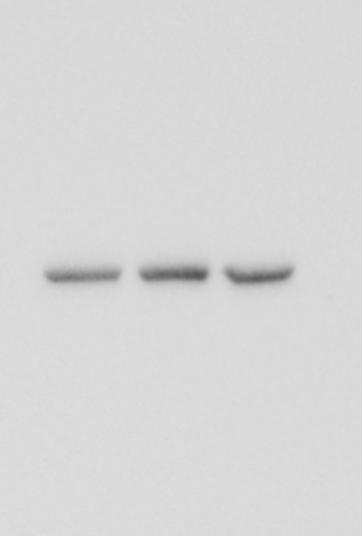

Supplement: Figure 6—figure supplement 1—source data 2. [file elife-106587-fig6-figsupp1-data2.zip › Figure 6-figure supplement 1 - source data 2/Figure 6-figure supplement 1C - source data 2/Figure 6-figure supplement 1C - 4E-BP2.tiff]

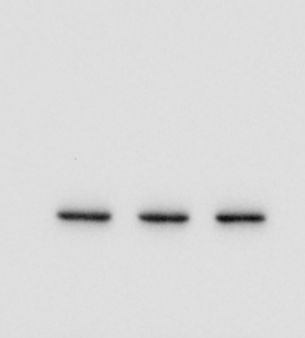

Supplement: Figure 6—figure supplement 1—source data 2. [file elife-106587-fig6-figsupp1-data2.zip › Figure 6-figure supplement 1 - source data 2/Figure 6-figure supplement 1C - source data 2/Figure 6-figure supplement 1C - S6.tiff]
