# Supplementary material for: Mitochondrial ETF insufficiency drives neoplastic growth by selectively optimizing cancer bioenergetics
Source: eLife. 2026 May 5;14:RP106587. doi: 10.7554/eLife.106587 (PMC13143275; doi:10.7554/eLife.106587)
Supplement: Figure 5—source data 1. [file elife-106587-fig5-data1.zip › Figure 5 - source data 1/Figure 5H - source data 1/Figure 5H - source data 1.pdf]

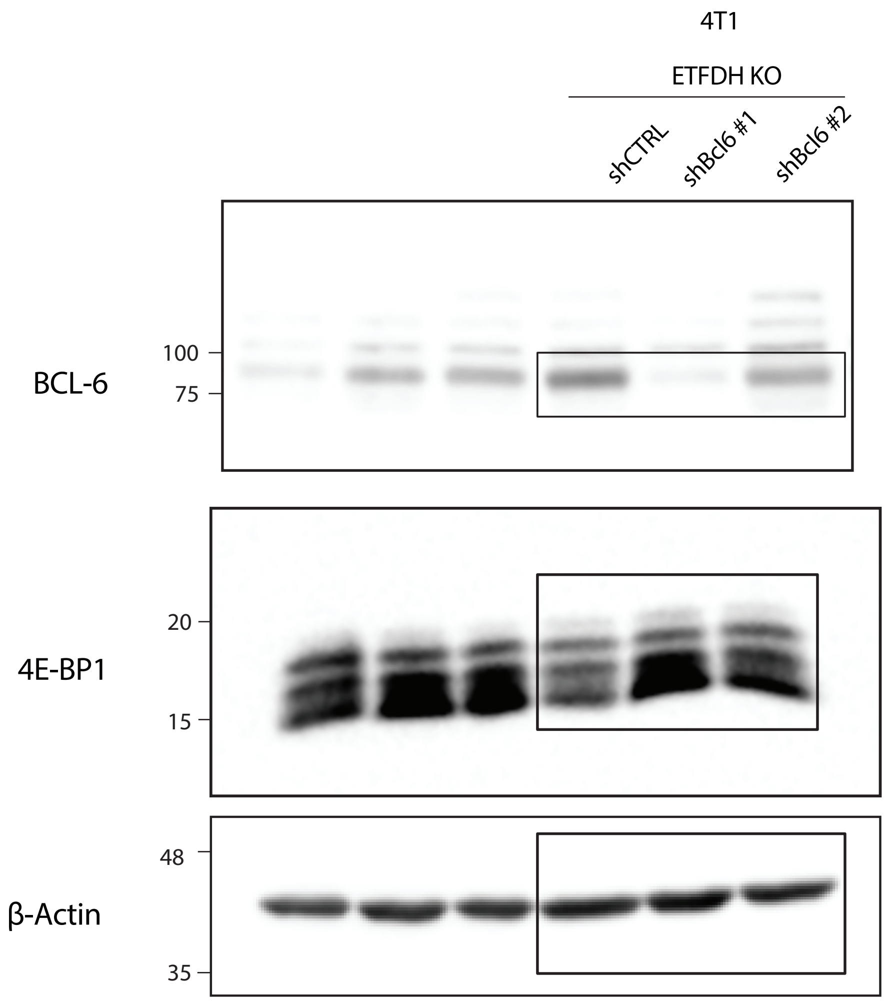

4T1
ETFDH KO
shCTRL
shBcl6 #1
shBcl6 #2
BCL-6
100
75
4E-BP1
20
15
β-Actin
48
35
