# Supplementary material for: Mitochondrial ETF insufficiency drives neoplastic growth by selectively optimizing cancer bioenergetics
Source: eLife. 2026 May 5;14:RP106587. doi: 10.7554/eLife.106587 (PMC13143275; doi:10.7554/eLife.106587)
Supplement: Supplementary file 1. [file elife-106587-supp1.docx]

Supplementary File 1 - List of Antibodies

| Antibody | Source | Identifier |
| --- | --- | --- |
|  |  |  |
| Mouse monoclonal β-actin antibody, 1:5000 dilution | MilliporeSigma | Cat#A5441; RRID: [AB_476744](http://antibodyregistry.org/AB_476744) |
| Normal Rabbit IgG | Cell Signaling Technologies | Cat#2729; RRID: [AB_1031062](http://antibodyregistry.org/AB_1031062) |
| Rabbit monoclonal 4E-BP1 (53H11) antibody, 1:1000 dilution | Cell Signaling Technologies | Cat#9644; RRID: [AB_2097841](http://antibodyregistry.org/AB_2097841) |
| Rabbit monoclonal Akt (pan) (C67E7) antibody, 1:1000 dilution | Cell Signaling Technologies | Cat#4691; RRID: [AB_915783](http://antibodyregistry.org/AB_915783) |
| Rabbit monoclonal phospho-4E-BP1 (S65) (174A9) antibody, 1:1000 dilution | Cell Signaling Technologies | Cat#9456; RRID: [AB_823413](http://antibodyregistry.org/AB_823413) |
| Rabbit monoclonal Phospho-Akt (Ser473) (D9E) XP® antibody, 1:1000 dilution | Cell Signaling Technologies | Cat#4060; RRID: [AB_2315049](http://antibodyregistry.org/AB_2315049) |
| Rabbit monoclonal Rictor (53A2) antibody, 1:1000 dilution | Cell Signaling Technologies | Cat#2114; RRID: [AB_2179963](http://antibodyregistry.org/AB_2179963) |
| Rabbit polyclonal phospho-S6 Ribosomal Protein (Ser240/244) antibody, 1:2000 dilution | Cell Signaling Technologies | Cat#2215; RRID: [AB_2630325](http://antibodyregistry.org/AB_2630325) |
| Rabbit polyclonal Raptor antibody, 1:1000 dilution | MilliporeSigma | Cat#09–217; RRID: [AB_612103](http://antibodyregistry.org/AB_612103) |
| Rabbit polyclonal 4E-BP2 antibody, 1:1000 dilution | Cell Signaling Technology | Cat#2845; RRID: [AB_10699019](http://antibodyregistry.org/AB_10699019) |
| Rabbit monoclonal phospho-S6K (T389) antibody, 1:500 dilution | Cell Signaling Technology | Cat#9234; RRID: [AB_2269803](http://antibodyregistry.org/AB_2269803) |
| Rabbit monoclonal S6K antibody, 1:1000 dilution | Cell Signaling Technology | Cat#2708; RRID: [AB_390722](http://antibodyregistry.org/AB_390722) |
| Mouse monoclonal S6 ribosomal protein antibody, 1:5000 dilution | Santa Cruz Biotechnology | Cat#sc-74459; RRID: [AB_1129205](http://antibodyregistry.org/AB_1129205) |
| Rabbit polyclonal ACC antibody, 1:1000 dilution | Cell Signaling Technology | Cat#3662; RRID: [AB_2219400](http://antibodyregistry.org/AB_2219400) |
| Rabbit polyclonal phospho-ACC (Ser79) antibody, 1:1000 dilution | Cell Signaling Technology | Cat#3661; RRID: [AB_330337](http://antibodyregistry.org/AB_330337) |
| Mouse monoclonal eIF2α (L57A5) antibody, 1:1000 dilution | Cell Signaling Technology | Cat#2103; RRID: [AB_836874](http://antibodyregistry.org/AB_836874) |
| Rabbit polyclonal eIF2α (Ser51) antibody, 1:1000 dilution | Cell Signaling Technology | Cat#9721; RRID: [AB_330951](http://antibodyregistry.org/AB_330951) |
| Rabbit polyclonal eIF4G1 antibody, 1:500 dilution | Cell Signaling Technology | Cat#2858; RRID: [AB_2095745](http://antibodyregistry.org/AB_2095745) |
| Rabbit polyclonal TFAM antibody, 1:1000 dilution | Cell Signaling Technology | Cat#7495; RRID: [AB_10841294](http://antibodyregistry.org/AB_10841294) |
| Mouse monoclonal eIF4E antibody, 1:1000 dilution | BD Biosciences | Cat#610269; RRID: [AB_397664](http://antibodyregistry.org/AB_397664) |
| Mouse monoclonal puromycin (12D10) antibody, 1:1000 dilution | Millipore | Cat#MABE343; RRID: [AB_2566826](http://antibodyregistry.org/AB_2566826) |
| Rabbit polyclonal AMPKα antibody, 1:500 dilution | Cell Signaling Technology | Cat#2532; RRI[D AB_330331](https://www.antibodyregistry.org/AB_330331) |
| Rabbit monoclonal phospho-AMPK-alpha (Thr172) antibody, 1:500 dilution | Cell Signaling Technology | Cat#2535; RRID[: AB_331250](https://www.antibodyregistry.org/AB_331250) |
| Rabbit polyclonal ETFDH antibody, 1:2000 dilution | ProteinTech | Cat#11109-1-AP; RRID[: AB_2231382](https://www.antibodyregistry.org/AB_2231382) |
| Rabbit monoclonal BCL-6 antibody, 1:1000 dilution | Cell Signaling Technology | Cat#14895; RRID[: AB_2798638](https://www.antibodyregistry.org/AB_2798638) |
| Rabbit monoclonal BCL-6 antibody, 1:1000 dilution | Cell Signaling Technology | Cat#5650; RRID[: AB_10949970](https://www.antibodyregistry.org/AB_10949970) |
| Rabbit monoclonal Snail antibody, 1:1000 dilution | Cell Signaling Technology | Cat#3879; RRID[: AB_2255011](https://www.antibodyregistry.org/AB_2255011) |
| Rabbit monoclonal Slug antibody, 1:1000 dilution | Cell Signaling Technology | Cat#9585; RRID[: AB_2239535](https://www.antibodyregistry.org/AB_2239535) |
| Rabbit polyclonal CREB-2 (ATF4) antibody, 1:1000 dilution | Santa Cruz | Cat#sc-200; RRID[: AB_2058752](https://www.antibodyregistry.org/AB_2058752) |
| Rabbit monoclonal STAT1 antibody, 1:1000 dilution | Cell Signaling Technology | Cat#14994; RRID[: AB_2737027](https://www.antibodyregistry.org/AB_2737027) |
| Rabbit monoclonal p-STAT1 (Tyr701) antibody, 1:1000 dilution | Cell Signaling Technology | Cat#9167; RRID[: AB_561284](https://www.antibodyregistry.org/AB_561284) |
| Rabbit monoclonal p27 antibody, 1:1000 dilution | Cell Signaling Technology | Cat#2947; RRID:AB_823586 |
