# Supplementary material for: Mitochondrial ETF insufficiency drives neoplastic growth by selectively optimizing cancer bioenergetics
Source: eLife. 2026 May 5;14:RP106587. doi: 10.7554/eLife.106587 (PMC13143275; doi:10.7554/eLife.106587)
Supplement: Supplementary file 2. [file elife-106587-supp2.docx]

Supplementary File 2: Primer sequences used for RT-qPCR

| Primer Name |  | Primer Sequence |
| --- | --- | --- |
| Human |  |  |
| *EIF4EBP1* forward | GGAACTCACCTGTGACCAAAA | |
| *EIF4EBP1* reverse | CCGCTTATCTTCTGGGCTATT | |
| *EIF4EBP2* forward | AGTCACTAGCCCTGGCACCTTA | |
| *EIF4EBP2* reverse | CTTGCAGGAGAGTCAGATGTCC | |
| *BCL6* forward | TGAGAAGCCCTATCCCTGTG | |
| *BCL6* reverse | TGTGACGGAAATGCAGGTTA | |
| *PP1A* forward | GTCAACCCCACCGTGTTCTT | |
| *PP1A* reverse | CTGCTGTCTTTGGGACCTTGT | |
| *GAPDH* forward | TGTTGCCATCAATGACCCCTT | |
| *GAPDH* reverse | CTCCACGACGTACTCAGCG | |
|  |  | |
| Mouse |  | |
| *Eif4ebp1* forward | GGTCACTAGCCCTACCAGCG | |
| *Eif4ebp1* reverse | TTGTGACTCTTCACCGCCTG | |
| *Eif4ebp2* forward | TACCTCAGGACTACTGCACCAC | |
| *Eif4ebp2* reverse | GGAGAATTGCGACGGTCCAACA | |
| *Bcl6* forward | GCGAACCTTGATCTCCAGTC | |
| *Bcl6* reverse | TGACTCTCACTGCTGCTTCG | |
| *Actb* forward | TTCCTTCTTGGGTATGGAA | |
| *Actb* reverse | CCACGATCCACACAGAGTA | |
