# Supplementary material for: Mitochondrial ETF insufficiency drives neoplastic growth by selectively optimizing cancer bioenergetics
Source: eLife. 2026 May 5;14:RP106587. doi: 10.7554/eLife.106587 (PMC13143275; doi:10.7554/eLife.106587)
Supplement: Supplementary file 3. [file elife-106587-supp3.docx]

Supplementary File 3: Primer sequences used for ChIP-qPCR

| Primer Name | Primer Sequence |
| --- | --- |
| *EIF4EBP1*_ChIP forward | TGACTGAGGTTCGGATCTGG |
| *EIF4EBP1*_ChIP reverse | CACTCGCGTAAATGGGGAAC |
| *EIF4EBP2*_ChIP forward | TTCAACAACTTCAGCCACGC |
| *EIF4EBP2*_ChIP reverse | GGAGTTGGGGAAGCTGGTC |
