# Supplementary material for: Mitochondrial ETF insufficiency drives neoplastic growth by selectively optimizing cancer bioenergetics
Source: eLife. 2026 May 5;14:RP106587. doi: 10.7554/eLife.106587 (PMC13143275; doi:10.7554/eLife.106587)
Supplement: Supplementary file 4. [file elife-106587-supp4.docx]

Supplementary File 4: gRNA sequences used for generation of ETFDH KO cell lines

| Primer Name | Primer Sequence |
| --- | --- |
| Human |  |
| *ETFDH* gRNA Target 1 Forward | CACCGAGGTTGGCCGAATGCTAGGA |
| *ETFDH* gRNA Target 1 Reverse | AAACTCCTAGCATTCGGCCAACCTC |
| *ETFDH* gRNA Target 2 Forward | CACCGATGTAGGGATACAAAAGGA |
| *ETFDH* gRNA Target 2 Reverse | AAACTCCTTTTGTATCCCTACATC |
| Mouse |  |
| *Etfdh* gRNA Target 1 Forward | CACCGGTGATACGCTGCATAAAAAT |
| *Etfdh* gRNA Target 1 Reverse | AAACATTTTTATGCAGCGTATCACC |
| *Etfdh* gRNA Target 2 Forward | CACCGGAACATCTTGGAGCACACAG |
| *Etfdh* gRNA Target 2 Reverse | AAACCTGTGTGCTCCAAGATGTTCC |
