# Supplementary material for: Mitochondrial ETF insufficiency drives neoplastic growth by selectively optimizing cancer bioenergetics
Source: eLife. 2026 May 5;14:RP106587. doi: 10.7554/eLife.106587 (PMC13143275; doi:10.7554/eLife.106587)
Supplement: Supplementary file 5. [file elife-106587-supp5.docx]

Supplementary File 5: Primer sequences used for sequencing

| Primer Name | Primer Sequence |
| --- | --- |
| Human |  |
| *ETFDH* validation Set 1 Forward | AGCGAGCATTTTCCTCCACA |
| *ETFDH* validation Set 1 Reverse | ACCATTGTTAGCTCAAAGTTGCT |
| *ETFDH* validation Set 2 Forward | TCATTTCCCATTGTGCGAGC |
| *ETFDH* validation Set 2 Reverse | ATGTACAGTTTGTAGCAAGACCT |
| Mouse |  |
| *ETFDH* validation Set 1 Forward | GTGTCTGAAGGCAGCAACAG |
| *ETFDH* validation Set 1 Reverse | GGATCAAGACAAGCCCCTGA |
| *ETFDH* validation Set 2 Forward | CCAGGTTCAATTCCCAGCAC |
| *ETFDH* validation Set 2 Reverse | CCGGGGATGAACAGTGTAGT |
